# Posttranslational Modification Cross‐Talk in Vascular Cells: Molecular Mechanisms and Therapeutic Implications for Atherosclerosis

**DOI:** 10.1002/cbf.70329

**Published:** 2026-11-01

**Authors:** Weiwei Jiang, Haiping Du

**Affiliations:** ^1^ Department of Cardiovascular Medicine The 970th Hospital of the Joint Logistic Support Force of the People's Liberation Army Yantai China; ^2^ Department of Cardiology Yantaishan Hospital Yantai Shandong China

**Keywords:** atherosclerosis, endothelial dysfunction, macrophage polarization, posttranslational modifications, vascular cell signaling

## Abstract

Atherosclerosis is driven by coordinated changes in endothelial cells (ECs), macrophages, and vascular smooth muscle cells (VSMCs). Post‐translational modifications (PTMs), phosphorylation, acetylation, ubiquitination, SUMOylation, O‐GlcNAcylation, palmitoylation, ISGylation, and ADP‐ribosylation, integrate metabolic, inflammatory, and mechanical inputs at a limited number of signaling nodes. This review argues that these modifications interact according to four definable relationships rather than merely co‐occurring. In competitive cross‐talk, two marks contest one acceptor residue, as when O‐GlcNAcylation displaces phosphorylation at the Akt site of endothelial nitric oxide synthase. In hierarchical cross‐talk, one modification licenses another, as when Ser536 phosphorylation of NF‐κB p65 recruits p300 and permits Lys310 acetylation. In enzyme‐directed cross‐talk, a modification acts on the writer or eraser of a different system, as HDAC9 does on IκB kinases. In cofactor‐coupled cross‐talk, sirtuin‐dependent deacylation and PARP1‐dependent ADP‐ribosylation draw on one limiting NAD^+^ pool. We rank nodes against four stated criteria, grade every finding on a five‐level evidence scheme, and apply the framework to phosphorylation–O‐GlcNAcylation, ubiquitination–SUMOylation, and acetylation–ubiquitination interactions across the three vascular cell types. Of the cross‐talk relationships invoked, five are supported at residue resolution by a substitution experiment, and two of these were obtained in vascular cells; the remainder are inferred from enzyme‐level regulation or from pathway convergence. Candidate intervention points include HDAC9, SENP3, OGT, UBA1, and the NAD^+^ pool, none of which has been evaluated in a cardiovascular outcome trial.

## Summary

- Atherosclerosis is the principal cause of ischaemic heart disease and stroke, and it involves coordinated changes in vascular cells that are regulated by posttranslational modifications (PTMs) such as phosphorylation and acetylation.
- This review explores how PTM interactions in endothelial cells, macrophages, and smooth muscle cells regulate inflammation, lipid buildup, and plaque stability.
- Key findings reveal that dysregulated PTMs converge on signaling hubs such as NF‐κB and Wnt/β‐catenin, with enzymes such as HDAC9 and SIRT1 as potential intervention points.
- By reframing PTMs as interconnected networks and grading the evidence behind each claim, this study identifies which mechanistic relationships are established and which are inferred and sets out the experiments that would allow PTM‐directed strategies to be tested alongside existing anti‐inflammatory and lipid‐lowering approaches.

**Abbreviations**
ABCA1: ATP‐binding cassette transporter A1
ABCG1: ATP‐binding cassette transporter G1
ADP: Adenosine diphosphate
ADP‐ribosylation/PARylation: Poly(ADP‐ribose)‐related protein modification
AKT: Protein kinase B
AKT/Mtor: Protein kinase B/mammalian target of rapamycin
AMPK: AMP‐activated protein kinase
AS: Atherosclerosis
ATF‐1: Activating transcription factor 1
CBP: CREB‐binding protein
CBX4: Chromobox homolog 4
CD36: Cluster of differentiation 36
CEL: Nε‐carboxyethyl‐lysine
CTRP9: C1q/TNF‐related protein 9
DKK1: Dickkopf‐related protein 1
DUB: Deubiquitinase
EC: Endothelial cell
EndMT: Endothelial‐to‐mesenchymal transition
eNOS: Endothelial nitric oxide synthase
ERBB4: Erb‐B2 receptor tyrosine kinase 4
ERK: Extracellular signal‐regulated kinase
ERK1/2: Extracellular signal‐regulated kinase 1/2
ERK5: Extracellular signal‐regulated kinase 5
FADD: Fas‐associated death domain
FBXL19: F‐box and leucine‐rich repeat protein 19
FBXO24: F‐box only protein 24
FoxM1: Forkhead box M1
FoxP1: Forkhead box P1
FTL: Ferritin light chain
GWAS: Genome‐wide association study
HDAC: Histone deacetylase
HDAC9: Histone deacetylase 9
HECT: Homologous to E6AP C‐terminus
HIF‐1α: Hypoxia‐inducible factor 1 alpha
HMGB1: High‐mobility group box 1
HO‐1: Heme oxygenase‐1
ICAM‐1: Intercellular adhesion molecule 1
IKK: IκB kinase
IL: Interleukin
IRF1: Interferon regulatory factor 1
ISG15: Interferon‐stimulated gene 15
ISGylation: ISG15 conjugation
JAK/STAT3: Janus kinase/signal transducer and activator of transcription 3
K48/K63: Lysine 48/lysine 63 ubiquitin linkage
KCa2.3: Small‐conductance calcium‐activated potassium channel 2.3
LDL: Low‐density lipoprotein
LOX‐1: Lectin‐like oxidized LDL receptor‐1
LRH‐1: Liver receptor homolog‐1
MAPK: Mitogen‐activated protein kinase
MCP‐1: Monocyte chemoattractant protein‐1
miR: microRNA
MLKL: Mixed lineage kinase domain‐like protein
mTOR: Mammalian target of rapamycin
mTORC1: Mammalian target of rapamycin complex 1
NAD^+^
: Nicotinamide adenine dinucleotide
NF‐κB: Nuclear factor kappa‐light‐chain‐enhancer of activated B cells
NLRP3: NOD‐, LRR‐, and pyrin domain‐containing protein 3
NO: Nitric oxide
NOX: NADPH oxidase
OGA: O‐GlcNAcase
OGT: O‐GlcNAc transferase
oxLDL: Oxidized low‐density lipoprotein
O‐GlcNAcylation: O‐linked β‐N‐acetylglucosamine modification
PA: Palmitic acid
PARP1: Poly(ADP‐ribose) polymerase 1
PDGF: Platelet‐derived growth factor
PGC‐1α: Peroxisome proliferator‐activated receptor gamma coactivator 1‐alpha
PI3K: Phosphoinositide 3‐kinase
PIEZO1: Piezo‐type mechanosensitive ion channel component 1
PINK1: PTEN‐induced kinase 1
PKA: Protein kinase A
PTM: Post‐translational modification
RAGE: Receptor for advanced glycation end products
RCT: Reverse cholesterol transport
RIPK1: Receptor‐interacting serine/threonine‐protein kinase 1
RIPK3: Receptor‐interacting serine/threonine‐protein kinase 3
ROCK1: Rho‐associated coiled‐coil containing protein kinase 1
ROS: Reactive oxygen species
SENP3: SUMO‐specific protease 3
SIRT: Sirtuin
SIRT1–SIRT7: Sirtuin 1 to sirtuin 7
SLC3A2: Solute carrier family 3 member 2
SMC: Smooth muscle cell
SRF: Serum response factor
SR‐A/SR‐AI: Scavenger receptor class A/class A type I
SR‐B1: Scavenger receptor class B type 1
SUMO: Small ubiquitin‐like modifier
SUMOylation: Small ubiquitin‐like modifier conjugation
TET1: Ten‐eleven translocation methylcytosine dioxygenase 1
TFEB: Transcription factor EB
TIMP3: Tissue inhibitor of metalloproteinases 3
TLR4: Toll‐like receptor 4
TRAF6: TNF receptor‐associated factor 6
TRIM: Tripartite motif‐containing protein
TRPV6: Transient receptor potential vanilloid 6
UBA1: Ubiquitin‐activating enzyme 1
UBC9: Ubiquitin‐conjugating enzyme E2 I
UCP2: Uncoupling protein 2
UPS: Ubiquitin‐proteasome system
USP: Ubiquitin‐specific protease
VCAM‐1: Vascular cell adhesion molecule 1
VEGFA: Vascular endothelial growth factor A
VSMC: Vascular smooth muscle cell
WEE1: WEE1 G2 checkpoint kinase
Wnt/β‐catenin: Wingless‐related integration site/beta‐catenin
WSS: Wall shear stress
XRCC1: X‐ray repair cross‐complementing protein 1
Zdhhc1: Zinc finger DHHC‐type palmitoyltransferase 1
ZKSCAN3: Zinc finger with KRAB and SCAN domains 3

## 1 Introduction

Atherosclerosis is a chronic, progressive disease of the arterial wall characterized by lipid deposition [1, 2, 3], inflammation [4, 5], and maladaptive tissue remodeling [6, 7]. Pathogenesis is an intercommunicative process of cellular interaction between ECs, macrophages, and VSMCs [8, 9, 10], triggered by a multifactorial combination of hemodynamic stresses and metabolic and immune stimuli [11, 12, 13]. Conventional models have focused on cytokine signaling [14, 15, 16] and lipid metabolism [17, 18], whereas recent evidence shows that PTMs are master molecular switches that coordinate upstream signals with downstream transcriptional and metabolic reprogramming [19, 20, 21, 22].

PTMs are covalent modifications of amino acid side chains, or of the protein backbone, that occur after translation and are, in most cases, enzymatically installed and removed [23, 24, 25, 26, 27]. These include phosphorylation, acetylation, ubiquitination, small ubiquitin‐like modifier conjugation (SUMOylation), O‐linked β‐N‐acetylglucosamine modification (O‐GlcNAcylation), palmitoylation, interferon‐stimulated gene 15 conjugation (ISGylation), and ADP‐ribosylation [28, 29]. They act in all cell types to regulate the activity, localization, stability, and interaction networks of crucial regulatory proteins [25, 30, 31].

Crucially, PTMs in atherosclerosis never occur in isolation. They form integrated regulatory networks that converge on a limited set of signaling nodes, including nuclear factor kappa‐light‐chain‐enhancer of activated B cells (NF‐κB) [32, 33], protein kinase B/mammalian target of rapamycin (AKT/mTOR) [34, 35], mitogen‐activated protein kinase (MAPK) [36, 37], Wingless‐related integration site/β‐catenin (Wnt/β‐catenin) [19], and the NOD‐, LRR‐, and pyrin domain‐containing protein 3 inflammasome (NLRP3 inflammasome) [38]. Convergence, however, is not the same as interaction, and the distinction is central to this review: cross‐talk between modifications is not a metaphor for shared pathway membership but a set of biochemical relationships that can be specified and, in principle, falsified, at the level of individual residues and enzymes [25, 26, 31]. Four such relationships account for the documented cases. In competitive cross‐talk, two modifications contest a single acceptor residue and are therefore mutually exclusive on a given protein molecule, as phosphorylation and O‐GlcNAcylation are on serine and threonine [39, 40, 41], which is the basis of their interplay in β‐catenin‐mediated vascular calcification [19], and as ubiquitination, SUMOylation, and acetylation are on the lysine ε‐amino group [24, 27, 42], where opposing SUMO and ubiquitin signals determine the fate of hypoxia‐inducible factor 1 alpha (HIF‐1α) [43]. In hierarchical cross‐talk, one modification creates or destroys the recognition determinant for the enzyme that installs another, as when poly(ADP‐ribose) chains direct ubiquitin conjugation to modified substrates [44], this example is drawn from nonvascular biochemistry and has not been examined in vascular cells (Section 7.5). In enzyme‐directed cross‐talk, a modification acts on the writer, eraser, or reader of a different pathway rather than on a shared substrate, as when histone deacetylase 9 (HDAC9) deacetylates the IκB kinases to sustain phosphorylation‐dependent NF‐κB signaling [21]. Finally, in cofactor‐coupled cross‐talk, two modifications that share neither substrate nor enzyme compete for one limiting metabolite, as sirtuin deacylases and PARP1 (Poly(ADP‐ribose) polymerase 1) do for NAD^+^, with the consequence that raising the cofactor can worsen rather than improve lesion burden [44, 45, 46, 47, 48].

Which of these relationships is operating, together with site specificity and cell type, determines whether a modification supports vascular protection through enhanced repair [49], autophagy [50], and restraint of plaque formation [51], or instead drives endothelial activation [52], foam cell formation [53], and calcification [19]. It also explains the mechanistic dualities reported at these nodes, such as phosphorylation of NF‐κB p65 driving macrophage inflammation [32] while ERK5 phosphorylation promotes endothelial resilience [54]. Section 7 applies this framework systematically, distinguishing cross‐talk demonstrated at residue resolution from cross‐talk currently inferred from pathway convergence.

In this review, we integrate current mechanistic knowledge across PTM classes, emphasize their interconnected and context‐dependent roles in vascular cells, and summarize emerging translational strategies for targeting complementary PTM pathways in atherosclerosis. By reframing PTMs as dynamic and interacting regulatory systems rather than isolated molecular events, we aim to provide a conceptual framework for more selective vascular interventions that may complement existing anti‐inflammatory and lipid‐lowering approaches.

## 2 Overview of Atherosclerosis Pathogenesis

AS, a chronic vasculopathy of large and medium‐sized arteries, is characterized by inflammatory reactions [55, 56, 57], deranged lipid metabolism [58, 59], and vascular plaque formation [60, 61]. Its pathogenesis reflects systemic interactions among endothelial dysfunction [62], maladaptive immune responses [63, 64], VSMC remodeling [65, 66], and extracellular matrix dysregulation [67], driven by lipid deposition and altered lipoproteins [68, 69] within the arterial wall. Of the three layers of the vessel, the innermost tunica intima comprises endothelial cells (ECs) that form a selectively permeable barrier, smooth muscle cells (SMCs), and pericyte‐like stellate cells [70, 71]. The endothelium senses circulating and haemodynamic signals and transduces them to the underlying vessel wall. The endothelium is exposed to mechanical stress (wall shear stress, WSS), elongation, and changes in the concentration of metabolic factors [72]. Atherosclerotic lesions develop primarily in areas of low WSS and flow separation, and most often at bifurcations and branch points [72].

AS is initiated by endothelial dysfunction with LDL retention and modification of the intima [71, 73]. Modified LDL and other atherogenic particles activate ECs and promote monocyte recruitment into the intima; the retained lipoproteins are then internalized by intimal macrophages and VSMCs, which become foam cells [74, 75]. Hemodynamic forces are key local drivers of endothelial dysfunction [76], particularly disturbed laminar flow generating low oscillatory shear stress at vulnerable sites [77, 78], which increases LDL retention and compromises endothelial integrity, permitting lipoprotein entry into the intima [79, 80, 81]. Mechanical stimulation also regulates endothelial gene expression [82, 83], inducing pro‐atherogenic genes such as MCP‐1 [84, 85] and PDGFs [86, 87], while suppressing protective genes such as eNOS [88, 89, 90].

Endothelial activation triggers regulated monocyte recruitment, rolling, adhesion, and chemokine/adhesion molecule‐mediated transmigration via MCP‐1/CCL2 [91, 92, 93, 94]. Monocytes differentiate into macrophages whose polarization state reflects the local inflammatory milieu [95, 96, 97], and which take up oxLDL through scavenger receptors including CD36, SR‐AI, and LOX‐1, driving cholesteryl ester deposition [98, 99, 100]. Impaired cholesterol efflux (ABCA1, ABCG1, SR‐B1) promotes foam cell formation and sustains inflammation [98, 101, 102], which further drives NF‐κB signaling and NLRP3 inflammasome activation, perpetuating monocyte recruitment, foam‐cell accumulation, and plaque growth [97, 103, 104, 105, 106]. This progression, from endothelial dysfunction through fibrous plaque formation to rupture, is depicted in Figure 1. Intimal VSMCs also internalize oxLDL through scavenger receptors, including SR‐A, CD36, and LOX‐1, in a manner that is not down‐regulated by intracellular cholesterol loading, and contribute substantially to the foam‐cell burden [107, 108, 109]; their lower ABCA1 expression relative to medial VSMCs impairs efflux and promotes lipid accumulation [110]. Notably, up to 50% of coronary intima foam cells are derived from VSMCs, underscoring their central role in AS [75, 110].

**Figure 1:**
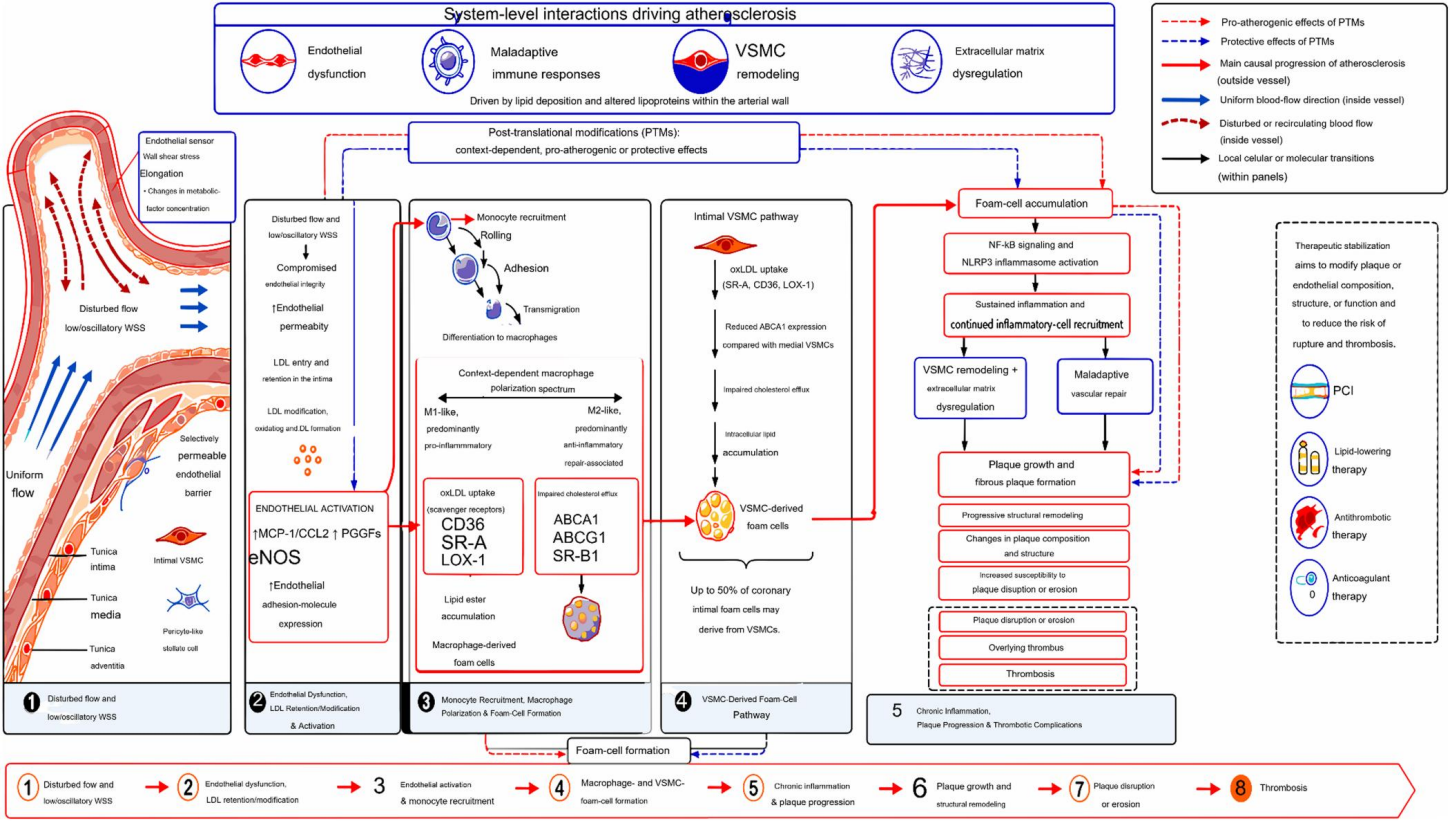
Overview of atherosclerosis pathogenesis. Disturbed flow and low, oscillatory wall shear stress promote endothelial dysfunction, LDL retention and modification, monocyte recruitment, and macrophage‐ and VSMC‐derived foam‐cell formation. Impaired cholesterol efflux and sustained NF‐κB/NLRP3‐mediated inflammation drive fibrous plaque progression, disruption or erosion, and thrombosis, while PTMs may exert context‐dependent pathogenic or protective effects and plaque stabilization may involve PCI and lipid‐lowering, antithrombotic, or anticoagulant therapies.

Plaque stabilization aims to alter plaque or endothelial composition, structure, or function so as to prevent or limit rupture [111]. Once plaque disruption or erosion with overlying thrombus has occurred, percutaneous coronary intervention combined with lipid‐lowering, antithrombotic, and anticoagulant therapy can achieve stabilization [112]. In summary, AS arises from endothelial dysfunction and lipoprotein retention, progresses through chronic inflammation and maladaptive repair, and culminates in fibrous plaque formation with the risk of rupture and thrombosis, an interplay of immune activation, lipid metabolism, and vascular remodeling. Increasingly, PTMs appear to exert major control over endothelial activation, foam cell formation, and plaque progression, though sometimes protectively, as reviewed in subsequent sections.

## 3 Molecular Switches in Vascular Disease: A Brief Overview of PTMs in Atherosclerosis

Each PTM class discussed here has a defined chemistry that constrains its interaction with the others, and this constraint is the basis of Section 7. Phosphorylation transfers a phosphate to the hydroxyl group of serine, threonine, or tyrosine [39, 40]; O‐GlcNAcylation transfers N‐acetylglucosamine to the same serine and threonine hydroxyls [41]; acetylation, ubiquitination, SUMOylation, and ISGylation all derivatize the lysine ε‐amino group [24, 27, 42, 113]; palmitoylation modifies cysteine thiols [114]; and ADP‐ribosylation transfers ADP‐ribose from NAD^+^, the same cofactor consumed by sirtuin deacylases [44]. Therefore, two chemically distinct groups of modifications compete for two chemical addresses, serine/threonine hydroxyls and lysine ε‐amines, and a third pair competes for a shared metabolite rather than a shared residue.

Functionally, each class behaves as a bidirectional switch rather than a unidirectional driver. Phosphorylation of NF‐κB p65 drives pro‐inflammatory transcription in macrophages [32], whereas ERK5 phosphorylation enhances endothelial antioxidant defense [54]; CBP‐dependent acetylation of NF‐κB increases adhesion molecule expression [52], whereas HDAC9‐dependent deacetylation of IKKα/β aggravates plaque vulnerability [21, 115, 116]; USP20 restrains NF‐κB signaling [117], whereas TRIM25 drives macrophage necroptosis [118]; SUMO2 conjugation of p66Shc promotes endothelial oxidative stress [119], whereas SENP3‐mediated deSUMOylation of NLRP3 suppresses inflammasome activation [38]; OGT‐dependent O‐GlcNAcylation of β‐catenin promotes VSMC calcification [19, 120]; Zdhhc1‐dependent palmitoylation of PI3K p110α promotes foam cell formation [121], whereas ciliary protein palmitoylation is protective [122]; ISG15 promotes macrophage cholesterol efflux [123], and ISGylation of p65 suppresses endothelial inflammation [124]; whereas ISG15 drives maladaptive smooth‐muscle remodeling [125], and PARP1‐dependent ADP‐ribosylation disrupts VSMC contractile programs [126, 127, 128]. Table 1 maps these events onto cell types and pathogenic axes. The direction of a modification is set by cell type, upstream stimulus, and acceptor site, and, as Section 7 argues, by which other modifications are simultaneously present. This bidirectionality is a general property of the modifications reviewed here rather than a feature of any one class; it is therefore stated once, here, and the sections that follow document the specific determinants of direction at each node rather than restating the principle.

**Table 1:** Posttranslational modifications mapped to their molecular targets and pathogenic axes in endothelial cells, macrophages and vascular smooth muscle cells.

PTM (writer/eraser)	Molecular target (residue where mapped)	Mechanism and functional outcome	Study system	Direction	Evidence	References
Endothelial cells						
Phosphorylation (PKN1)	Histone H3.3 Ser31	Disturbed flow activates PKN1 via integrin α5β1 and drives its nuclear translocation. H3.3S31 phosphorylation permits the rapid induction of FOS and FOSB. Endothelium‐specific PKN1 loss or an S31 phospho‐deficient H3.3 mutant reduces endothelial inflammation.	HUAECs; endothelial PKN1‐knockout and H3.3 S31A mice	Pro‐atherogenic	E3‡	[22]
Phosphorylation (GRK2)	Vinculin Ser721	VCL^S721p^ locks vinculin in an inactive, closed conformation, disrupting the VE‐cadherin/catenin complex and increasing permeability. Serum sVCL^S721p^ tracks atherosclerotic stage in patients	Porcine phosphoproteomics; transgenic mice; human coronary specimens	Pro‐atherogenic	E3‡ with E2 support	[129]
Phosphorylation (p38)	c‐REL program via TXNIP (site not mapped)	Disturbed flow drives TXNIP–p38 cross‐talk with noncanonical NF‐κB, sustaining adhesion molecule expression at low‐shear sites	Disturbed‐flow models; hyperlipidaemic mice	Pro‐atherogenic	E3	[33]
Phosphorylation (IKK activation)	IKK complex (site not mapped)	MS4A6A promotes IKK/NF‐κB activation, increasing monocyte adhesion after oxLDL exposure	Human ECs (oxLDL)	Pro‐atherogenic	E4	[130]
Phosphorylation (ERK1/2, ERK5, AMPK)	ERK1/2 → IRF1 → VCAM‐1 [131] ERK5 [54] K_Ca_2.3 channel [132, 133]	Three independent protective routes: IL‐33‐driven ERK1/2 suppresses VCAM‐1 and leukocyte adhesion; ERK5 activation strengthens antioxidant defence against oxLDL; AMPK acts on K_Ca_2.3 to restore hyperpolarisation and NO bioavailability	Apoe^−/−^ mice; oxLDL‐exposed ECs; angiotensin II and diet‐induced obesity models	Protective	E3/E4	[54, 131, 132, 133]
Acetylation (CBP)	NF‐κB p65	CBP‐dependent p65 acetylation is required for thrombin‐mediated endothelial activation; CBP knockdown blocks it	Human ECs (thrombin)	Pro‐atherogenic	E4	[52]
Deacetylation (HDAC9)	IKKα and IKKβ [21] Endothelial–mesenchymal transition program [116]	HDAC9 binds and deacetylates IKKα/β, activating them and driving inflammatory responses in endothelial cells as well as macrophages; HDAC9 also promotes EndMT and an unfavorable plaque phenotype	Hyperlipidaemic mice with bone‐marrow reconstitution; TMP195; endothelial Hdac9 deletion	Pro‐atherogenic	E3	[21, 116]
SUMOylation (SUMO2)	p66Shc	SUMO2 conjugation of p66Shc increases oxidative stress and reduces NO bioavailability	Endothelial cells	Pro‐atherogenic	E4†	[119]
SUMOylation (hypoxia‐induced)	FADD	Hypoxia‐driven FADD SUMOylation activates the RIPK1–RIPK3–MLKL axis, causing necroptotic endothelial injury	Endothelial cells under hypoxia	Pro‐atherogenic	E4	[134]
ISGylation (SCF^FBXL19^)	NF‐κB p65 (acceptor lysines not reported)	ISGylation of p65 stabilizes the p65–WIP1 interaction and reduces inflammatory serine phosphorylation, diminishing NF‐κB‐driven endothelial inflammation. Demonstrated in acute lung injury models rather than atherosclerosis.	Endothelial cells; murine acute lung injury models	Protective	E3/E4	[124]
Macrophages and myeloid cells						
Phosphorylation (WEE1)	NF‐κB p65 Ser536	WEE1 binds p65 directly and phosphorylates Ser536, inducing inflammatory transcription and driving lesion growth	Macrophages; Apoe^−/−^ mice	Pro‐atherogenic	E3	[32]
Phosphorylation (site disrupted by knock‐in)	LXRα Ser196	Myeloid S196A substitution reprogrammed the macrophage transcriptome, inducing FoxM1 and plaque‐resident cell proliferation, and increasing efferocytosis. Plaque burden rises, but necrotic cores are smaller and fibrous caps thinner	Myeloid LXRα S196A knock‐in on *Ldlr* ^−/−^ background, Western diet	Context‐dependent	E3‡	[135]
Phosphorylation (p38)	ATF‐1	Calycosin‐7‐glucoside activates ATF‐1 through p38/MAPK, reducing oxLDL‐induced foam‐cell formation and inflammatory mediators	THP‐1‐derived macrophages	Protective	E4	[136]
Deacetylation (HDAC9)	IKKα and IKKβ [21] NLRP3 NACHT and LRR domains [115]	HDAC9 deacetylation activates IKK and licenses inflammasome activation. Myeloid‐restricted deletion of the HDAC9 cis‐regulatory element raises HDAC9 and exacerbates disease; HDAC9 expression in human carotid endarterectomy tissue tracks plaque instability	Hyperlipidaemic mice; TMP195 and TMP195 nanoparticles; human carotid specimens	Pro‐atherogenic	E3 with E2 support	[21, 115]
Deacetylation (SIRT1, SIRT2)	PGC‐1α [45] TIMP3/ADAM17 axis [46] Macrophage polarization program [51]	SIRT1‐dependent PGC‐1α deacetylation restores mitochondrial function; SIRT1 restrains the inflammatory response via TIMP3/ADAM17; SIRT2 shifts macrophage polarization away from an inflammatory phenotype	Apoe^−/−^ and *Ldlr* ^−/−^ mice; macrophages	Protective	E3	[45, 46, 51]
Deacetylation (SIRT1)	ZKSCAN3 (acceptor lysines not mapped)	Nε‐carboxyethyl‐lysine dysregulates RAGE/LKB1/AMPK/SIRT1 signaling, altering ZKSCAN3 acetylation, impairing macrophage autophagy and promoting plaque instability in diabetic atherosclerosis	Macrophages; diabetic atherosclerosis models	Pro‐atherogenic	E3	[50]
SUMOylation (CBX4)	HIF‐1α	Macrophage CBX4 acts as a SUMO E3 ligase on HIF‐1α and *potentiates* atherosclerosis Whether SUMO and ubiquitin contest the same HIF‐1α lysines has not been tested in vascular cells	Macrophages; atherosclerosis models	Pro‐atherogenic	E3	[43]
deSUMOylation (SENP3)	NLRP3 (SUMO2/3 removal)	SENP3‐mediated deSUMOylation of NLRP3 attenuates foam‐cell formation; SENP3 loss of function increases inflammasome activation and lesion burden	Macrophages (oxLDL); mice	Protective	E3	[38]
Palmitoylation (ZDHHC1)	PI3K catalytic subunit p110α	ZDHHC1‐dependent palmitoylation retains p110α outside the nucleus and sustains PI3K–AKT–mTOR signaling; Zdhhc1 deficiency drives p110α nuclear translocation and reduces foam‐cell formation	Apoe^−/−^ mice; bone‐marrow‐derived macrophages	Pro‐atherogenic	E3	[121]
Deubiquitination (USP20)	TRAF6, acting with β‐arrestin2 (TLR4 → NF‐κB axis)	USP20 regulates the reciprocal functions of β‐arrestin2 in TLR4‐promoted NF‐κB activation, restraining inflammatory signaling	Cell‐based mechanistic studies	Protective	E4	[117]
Deubiquitination (USP18)	ABCG1 (*reported*)	USP18 deubiquitinates ABCG1 and promotes cholesterol efflux. Caveat: USP18 cleaves ISG15 conjugates and has undetectable activity toward ubiquitin conjugates in vitro, so an ISG15‐directed rather than ubiquitin‐directed mechanism cannot be excluded (see Section 7.6)	Macrophages; atherosclerosis models	Reported Protective	E4 (mechanism contested)	[137]
Vascular smooth muscle cells						
O‐GlcNAcylation (OGT)	β‐catenin (acceptor residues not mapped in VSMCs)	OGT‐dependent O‐GlcNAcylation increases β‐catenin stability and transcriptional activity, thereby promoting Wnt‐dependent osteogenic conversion. Smooth‐muscle‐restricted *Ogt* deletion attenuates mineralization and reduces lesion burden.	VSMCs; smooth‐muscle *Ogt*‐knockout and hyperglycaemic Apoe^−/−^ mice	Pro‐atherogenic	E3	[19, 120]
O‐GlcNAcylation (OGT)	AKT Thr430 and Thr479	O‐GlcNAcylation at these two residues promotes AKT Ser473 phosphorylation and mTORC2 binding. Substitution of both residues blocks Runx2 transactivity and VSMC calcification. Thiamet‐G‐induced O‐GlcNAc elevation accelerates calcification in diabetic mice	Primary mouse VSMCs; streptozotocin‐induced diabetic mice	Pro‐atherogenic	E3‡	[138]
ADP‐ribosylation (PARP1)	Stat1/Runx2 axis [126] TET1 [127] SRF and myocardin [128]	PARP1 activity drives osteogenic and synthetic reprogramming and extracellular matrix deposition; PARP1 loss or inhibition attenuates calcification and restores contractile gene expression	VSMCs; diabetic and atherosclerotic mouse models	Pro‐atherogenic	E3	[126, 127, 128]
SUMOylation (E3 ligase not identified)	Serum response factor (SRF; acceptor lysines not mapped) |	SRF SUMOylation modulates the VSMC phenotypic switch and vascular remodeling. SRF is also poly(ADP‐ribosyl)ated [128], making it a shared substrate of two modification systems with opposite consequences (Section 7.6)	VSMCs; vascular remodeling models	Pro‐atherogenic	E3	[139]
ISGylation (ISG15)	VSMC phenotypic program	ISG15 modulates VSMC phenotype and contributes to pathological vascular remodeling. Genetic ablation of ISG15 increased contractile‐program gene expression at the expense of osteogenic markers and calcification	VSMCs; vascular remodeling models; human aortic tissue	Pro‐atherogenic	E3	[125]

*Note:* Evidence; E3, in vivo perturbation of a defined enzyme in an animal model with a vascular outcome; E4, in vitro mechanistic evidence with a defined enzyme and substrate; E5, associative or computational evidence without functional validation. ‡, acceptor residue mapped and tested by substitution; may be appended to any grade. †, source has not completed peer review. Residue‐resolved entries. Four entries meet the ‡ standard: Histone H3.3 Ser31 [22], vinculin Ser721 [133], LXRα Ser196 [129], and AKT Thr430/Thr479 [140]. Across all three cell types these are the only modifications in this table for which an acceptor residue has been mapped and tested by substitution. Non‐peer‐reviewed source. One entry carries †: The SUMO2–p66Shc relationship [119] rests on a preprint, and the corresponding statement in Section 6.4.2 is worded accordingly. Contested entry. The USP18–ABCG1 entry [141] is retained with an explicit caveat rather than removed, because the reported ubiquitin‐directed mechanism is difficult to reconcile with the ISG15 specificity of USP18 [142]; Section 7.6 sets out the argument.

## 4 Prioritizing PTM‐Regulated Signaling Nodes in Atherosclerosis

Sections 6.1–6.9 examine PTM classes individually. Before doing so, it is necessary to state which signaling nodes justify that attention, because the vascular PTM literature is expanding considerably faster than it is being ranked, and a review that treats every reported pathway as equally informative reproduces that problem rather than resolving it. We therefore prioritize nodes against four criteria (Table 2), chosen because each can be applied to any candidate node and audited by the reader: (i) PTM‐class convergence, the number of chemically distinct modification classes documented to act on the node or its proximal regulators, since convergence is what distinguishes an integration point from a relay; (ii) cell‐type breadth, whether the node operates in endothelial cells, macrophages, and vascular smooth muscle cells, or in a single compartment; (iii) human anchoring, whether human genetic association or human plaque tissue data exist, as distinct from murine or cell‐culture evidence alone; and (iv) interventional evidence, whether targeting the node has been shown to alter lesion burden in vivo. Table 1 summarizes representative PTMs and their cellular targets; Table 2 applies the four criteria and assigns the three tiers that determine the emphasis of the sections that follow.

**Table 2:** Prioritization of post‐translational modification (PTM)‐regulated signaling nodes in atherosclerosis, assessed against four pre‐specified criteria.

Signaling node	(i) Converging PTM classes	(ii) Cell types	(iii) Human anchoring	(iv) Interventional evidence in vivo	Tier and basis
Tier 1—Highest informational value; unmet criteria stated explicitly^a,^ ^d^					
NF‐κB–IKK axis	Five. Phosphorylation: WEE1 → p65 Ser536 [32], disturbed flow→c‐REL via TXNIP–p38 [33], MS4A6A → IKK [130]. Acetylation: CBP→p65 [52]. Deacetylation: HDAC9 → IKKα/β [21]. ISGylation: SCF(FBXL19) → p65 [124]. ADP‐ribosylation: Inferred from PARP inhibition but not directly demonstrated [143].^e^	EC, Mφ, VSMC	HDAC9 rs2107595 is associated with advanced carotid atherosclerosis [144] and CAD susceptibility and lesion severity [145], and HDAC9 expression in carotid endarterectomy tissue [115]. No PTM‐directed agent has been tested in an outcome trial.	Class IIa HDAC inhibition (TMP195; TMP195 nanoparticles) reduces lesion growth and increases fibrous cap thickness [21, 115]. UBA1 inhibition (PYR‐41) reduces plaque burden without altering systemic lipids [146].	Tier 1: All four criteria are met. This is the most heavily co‐modified node identified, and the only one whose human genetic anchoring is supported by functional characterization.
NLRP3 inflammasome	Two plus a lesion‐derived stimulus. Deacetylation: HDAC9 → NLRP3 NACHT and LRR domains [115]. DeSUMOylation: SENP3→removal of SUMO2/3 [38]. Cholesterol crystals are activating inputs [105, 106].	Predominantly Mφ	HDAC9 expression and inflammasome readouts measured in human carotid endarterectomy specimens, with a reported association with plaque instability [115]. Observational only.	TMP195‐loaded nanoparticles stabilized plaques [115]. SENP3 loss of function increased NLRP3 SUMOylation, inflammasome activation, and lesion burden [38].	Tier 1 by exception^d^; criteria (i), (iii), and (iv) met, (ii) not met, the evidence is predominantly myeloid. In vivo data exist for both classes. Residue overlap between the two lysine marks was untested.
NAD^+^‐dependent deacylation–PARylation axis	Two, cofactor‐coupled. Sirtuin deacylation [45, 46] and PARP1 ADP‐ribosylation [44, 47] consume a shared NAD^+^ pool, with no shared substrate or enzyme.	Mφ, VSMC, EC	None identified	NAD^+^ supplementation increased plaque area and inflammation, with CD38 upregulation and NAD^+^ redirected toward PARP1 rather than sirtuins [48]. PARP1 inhibition replenished NAD^+^ and preserved VSMC viability in a progeria model [47].	Tier 1 by exception^c^, criteria (i), (ii), and (iv) met; (iii) not met. The only node with in vivo evidence that the interaction, rather than either enzyme alone, sets the phenotype.
Tier 2—Satisfies three of four criteria					
PI3K/AKT/mTOR	Three. Phosphorylation at several cascade steps [34, 35, 147, 148, 149, 150]. Palmitoylation: Zdhhc1 → PI3K p110α, which determines its localization [121]. O‐GlcNAcylation: AKT Thr430/Thr479 O‐GlcNAcylation promotes Ser473 phosphorylation and mTORC2 binding, with combined substitution of both residues blocking Runx2 transactivation and calcification [138]	EC, Mφ, VSMC	None identified	Largely agents of limited target selectivity: leucine [147], lactoferrin [148], notoginsenoside R1–saikosaponin B2 [149], and an miRNA mimic [150]. Defined genetic evidence is limited to Zdhhc1 deficiency [121].	Tier 2, (i) and (ii) met; (iii) not met; (iv) met only by agents of uncertain selectivity
Wnt/β‐catenin–Runx2 osteogenic axis	Two. O‐GlcNAcylation: O‐GlcNAcylation: OGT→β‐catenin, reported to increase stability and transcriptional activity[19]; O‐GlcNAcylation of AKT at Thr430/Thr479, which promotes Ser473 phosphorylation and Runx2 transactivation [138]. Phosphorylation is the second class, but no β‐catenin phosphorylation site has been mapped in VSMCs.	VSMC only	None identified	Smooth muscle‐restricted *Ogt* deletion attenuated phosphate‐induced mineralization, an effect reversed by pharmacological Wnt activation [19]. *Ogt* deletion reduces lesion burden and restores contractile markers in hyperglycemic *Apoe*‐deficient mice [120].	Tier 2, mechanistically the sharpest design (loss of signal plus pharmacological restoration), but one cell type, one disease process, and no human tissue data
Tier 3—Explicitly de‐prioritized					
MAPK cascades (ERK1/2, ERK5, p38)	One (phosphorylation). The direction of the effect is isoform‐inverting: ERK5 activation reported to be atheroprotective [54], IL‐33 → ERK1/2 suppresses IRF1‐dependent VCAM‐1 and leukocyte adhesion [131], and p38 is reported to be pro‐atherogenic [33, 37].	EC, Mφ, VSMC	None identified	Natural products of uncertain selectivity: Fucosterol [36], resveratrol [151], and icariin [152]. Calycosin, nominated by network pharmacology and in‐silico docking without experimental validation [153].	Tier 3, de‐prioritized, a downstream effector on which PTM signals are read out rather than an integration point. Opposing p38 and ERK5 results describe two distinct nodes, not one conflict.

*Note:* ^a^Tier assignment is a claim about informational value, not about biological importance, and is deliberately revisable: A Tier 2 node would move upward if human plaque data or a selective intervention became available. ^b^Acetylation and deacetylation are counted as one chemical class, acyl transfer to the lysine ε‐amino group, written by acetyltransferases and erased by deacetylases. Counting writer and eraser separately would inflate the convergence score. ^c^The NAD+‐dependent axis qualifies not because several modifications converge on one protein but because two modification systems compete for one limiting cofactor. Criterion (iii) is not met, and the exception is stated rather than concealed. ^d^Two nodes are placed at Tier 1 despite failing one criterion; in both cases the failure is stated in the row rather than absorbed into the tier label. The NAD+ axis fails (iii); the NLRP3 inflammasome fails (ii). A reader who disagrees with either exception can move the node to Tier 2 without disturbing the rest of the table. ^e^ **Evidence grades** (Section 5). E1, human causal evidence; E2, human observational evidence; E3, in vivo perturbation of a defined enzyme in an animal model with a vascular outcome; E4, in vitro mechanistic evidence with a defined enzyme and substrate; E5, associative or computational evidence without functional validation. ‡, acceptor residue mapped and tested by substitution; may be appended to any grade. †, source has not completed peer review.

Three nodes carry the analytical weight of this review. One satisfies all four criteria outright; two are admitted to Tier 1 by stated exception, with the unmet criterion named in Table 2 rather than absorbed into the tier label. The NF‐κB–IKK axis is the most heavily co‐modified structure in vascular biology: WEE1‐mediated phosphorylation of p65 at Ser536 [32], disturbed‐flow c‐REL activation through p38 [33], receptor‐driven IKK signaling [130], CBP‐mediated acetylation [52], HDAC9‐mediated deacetylation of IKKα/β [21], SCF^FBXL19^‐mediated ISGylation of p65 [124], and ADP‐ribosylation inferred from the effects of PARP inhibition [143] converge on one transcriptional output, in all three vascular cell types. It is anchored in humans by HDAC9 risk variants associated with carotid and coronary atherosclerosis [144, 145] and by HDAC9 expression in carotid endarterectomy tissue [115], and it is interventionally supported by class IIa HDAC inhibition [21, 115] and by UBA1 inhibition [146]. The NLRP3 inflammasome is the second, admitted by stated exception on criterion (ii): it integrates deacetylation [115] and deSUMOylation [38] with cholesterol‐crystal‐driven activation [105, 106], is measurable in human plaque [115], and responds to both pharmacological [115] and genetic [38] perturbation in vivo, but the vascular evidence is predominantly myeloid rather than distributed across all three compartments. The third qualifies by a different route. The NAD^+^‐dependent deacylation–PARylation axis is a node not because multiple modifications converge on one protein but because two modification systems compete for one cofactor: sirtuins [45, 46] and PARP1 [44, 47] draw on a shared NAD^+^ pool, and the demonstration that NAD^+^ supplementation *increases* plaque burden by redirecting NAD^+^ toward PARP1 and away from sirtuins [48] establishes this as an intervention point at which two PTM systems are non‐independent. We regard this as the most underrecognized‐ node in the field, and it is developed further in Section 7.1.

Two nodes satisfy three of the four criteria and are treated in proportion. **AKT/mTOR** shows genuine multiclass convergence, phosphorylation at several steps [34, 35, 147, 148, 149, 150], palmitoylation of the p110α catalytic subunit determining its subcellular distribution [121], and sustained AKT activity accompanying O‐GlcNAc‐driven Runx2 activation in calcifying smooth muscle [154], and operates across all three compartments, but it lacks human anchoring, and its interventional evidence rests substantially on agents of limited target selectivity. The **Wnt/β‐catenin–Runx2 osteogenic axis** is mechanistically the sharpest node considered here, since smooth‐muscle‐restricted *Ogt* deletion attenuates phosphate‐induced mineralization and pharmacological Wnt activation restores it [19]; it is nonetheless confined to one cell type and one disease process, and is likewise unanchored in human tissue.

One heavily represented family warrants explicit de‐prioritization. **MAPK cascades** occupy a substantial share of the vascular PTM literature; however, they rank lowest on these criteria. They are regulated by a single modification class; their direction of effect is not merely context‐dependent‐ but isoform‐inverting, with ERK5 activation atheroprotective [54], IL‐33‐driven ERK1/2 signaling suppressing VCAM‐1 and leukocyte adhesion [131], and p38 signaling pro‐atherogenic [33, 37]; and the interventional literature is dominated by natural products of uncertain target selectivity, in some cases without experimental validation [36, 151, 152, 153]. MAPK is better understood as a downstream effector on which PTM signals are read out than as an integration point at which they are combined. We therefore report MAPK findings where they bear on a Tier 1 or Tier 2 node and do not treat the cascade as a target in its own right. This distinction resolves much of the apparent contradiction in this literature: opposing results from p38 and ERK5 studies are not conflicting evidence about one node but concordant evidence about two.

This tiering is a claim about informational value rather than biological importance, and it is deliberately revisable: a Tier 2 node would move upward if human plaque data or a selective intervention became available. Stating the criteria explicitly allows such reassessment to be made openly rather than by the accumulation of citations, and it is the standard against which the sections that follow should be read. Table 1 maps representative PTMs to their cellular targets and pathogenic axes across endothelial cells, macrophages, and vascular smooth muscle cells, and should be read alongside Sections 6.1–6.9. Section 7 examines the cross‐talk architectures through which the Tier 1 nodes are co‐regulated.

## 5 Grading the Evidence Base

The prioritization above presumes that the studies underlying each node are comparable; however, they are frequently not. Research on vascular posttranslational modifications draws on cell culture, animal models, human genetic association, human tissue analysis, observational biomarker measurement, and computational inference. These designs license claims of markedly different strengths, and a review that reports them in a uniform declarative voice implies an equivalence that does not exist. We therefore assign every finding presented in Tables 1, 2, 3, 4, 5, 6, 7, 8 an evidence grade, and we state the grade in the text wherever it changes how a result should be read.

**Table 3:** Phosphorylation‐driven posttranslational modifications and their roles in the pathogenesis and progression of atherosclerosis.

Study system	Phosphorylation target (residue where mapped)	Upstream kinase/regulator	Downstream mechanism	Functional outcome and impact on atherosclerosis	Evidence	References
A. Phosphorylation‐driven inflammatory signaling						
Bone‐marrow‐derived and THP‐1 macrophages; ApoE^−/−^ mice	NF‐κB p65 Ser536	WEE1 (direct binding and phosphorylation)	p65 Ser536 phosphorylation → NF‐κB‐dependent inflammatory transcription	↑ Inflammatory gene expression; ↑ lesion growth (pro‐atherogenic)	E3	[32]
Human umbilical artery ECs under disturbed flow; endothelium‐specific PKN1‐knockout mice; endothelial H3.3 S31A mutant mice	Histone H3.3 Ser31	PKN1, activated by disturbed flow in an integrin α5β1‐dependent manner and translocated to the nucleus	H3.3S31 phosphorylation rapidly induces FOS and FOSB, which are required for inflammatory gene expression. PKN1 separately phosphorylates the AP‐1 subunit JUN	Endothelium‐specific PKN1 loss or S31 phospho‐deficient H3.3 reduced endothelial inflammation and disturbed‐flow‐induced vascular remodeling (pro‐atherogenic when intact)	E3‡	[22]
Endothelial disturbed‐flow models; hyperlipidaemic mice	p38 MAPK (site not mapped)	Disturbed flow; TXNIP	TXNIP–p38 cross‐talk with non‐canonical NF‐κB sustaining an endothelial c‐REL program	↑ Adhesion molecule expression; ↑ plaque burden at low‐shear sites (pro‐atherogenic)	E3	[33]
Human ECs exposed to oxLDL	IKK complex (site not mapped)	MS4A6A	IKK/NF‐κB activation	↑ Monocyte adhesion; endothelial dysfunction (pro‐atherogenic)	E4	[130]
Apoe^−/−^ mice; endothelial assays	ERK1/2 (increased phosphorylation; site not mapped)	IL‐33/ST2	ERK1/2 → IRF1 → suppression of VCAM‐1	↓ Leukocyte adhesion; ↓ lesion formation (protective)	E3	[131]
Macrophages in vitro; atherosclerosis‐prone mice	p38 MAPK and ERK (reduced phosphorylation)	Fucosterol (marine phytosterol)	Attenuation of NF‐κB and p38/ERK MAPK signaling	↓ Cytokine production; ↓ foam‐cell formation; ↓ plaque size (protective)	E3	[36]
THP‐1‐derived macrophages (oxLDL)	ATF‐1 (activation downstream of p38)	Calycosin‐7‐glucoside	p38/MAPK‐dependent ATF‐1 activation	↓ Foam‐cell formation; ↓ inflammatory mediators (protective)	E4	[136]
Vascular cells; ferroptosis models	MAPK1/ERK2 (modulated)	Resveratrol	MAPK1‐dependent control of ferroptosis and lipid peroxidation	↓ Lipid peroxidation; ↓ inflammatory injury (protective)	E4	[151]
Carotid injury model with mesenchymal stem cell therapy	ERK (increased phosphorylation)	miR‐126 (MSC‐associated)	MAPK/ERK pro‐reparative signaling	↑ Re‐endothelialisation and vascular repair (protective)	E3	[49]
Coronary endothelial models	PKA‐dependent signaling (substrate residues not identified)	TRPV6 overexpression → PKA	PKA/UCP2 cytoprotective axis	↓ Inflammatory response; ↓ apoptosis (protective)	E4	[155]
B. PI3K/AKT/mTOR and MAPK cascades						
Macrophages; atherosclerosis‐prone mice	mTORC1 (site not specified)	Leucine (dietary amino acid)	Dose‐dependent mTOR activation → autophagy suppression	↑ Lipid accumulation; accelerated plaque progression (pro‐atherogenic)	E3	[147]
Apoe^−/−^ mice; macrophages	mTOR (reduced phosphorylation)	Lactoferrin	AMPK/mTOR‐dependent autophagy	↑ Autophagic flux; ↓ foam‐cell formation (protective)	E3	[148]
Apoe^−/−^ mice; macrophages	AKT/mTOR (reduced phosphorylation)	Notoginsenoside R1 + saikosaponin B2	PI3K/AKT/mTOR pathway and macrophage autophagy	↑ Autophagy; ↓ lipid deposition (protective)	E3	[149]
Macrophages; atherosclerosis‐prone mice	mTOR/ULK1 (modulated phosphorylation)	miR‐125b‐1‐3p, acting via RRAGD	RRAGD/mTOR/ULK1 autophagy signaling	↑ Autophagy; ↓ plaque burden (protective)	E3	[150]
Apoe^−/−^ mice; macrophages	AKT–mTOR (reduced phosphorylation)	Zdhhc1 deficiency (loss of p110α palmitoylation)	Nuclear translocation of PI3K p110α → suppressed PI3K–AKT–mTOR signaling	↓ oxLDL uptake; ↓ foam‐cell formation; attenuated atherosclerosis (protective)	E3	[121]
VSMCs and ECs; atherosclerosis models	AKT, mTOR and ROCK1 (increased phosphorylation)	S1PR2 activation	Akt/mTOR and RhoA/ROCK1 signaling	↑ SMC proliferation; ↑ endothelial permeability (pro‐atherogenic)	E4	[35]
Endothelial cells; atherosclerosis models	AKT/mTOR (increased phosphorylation)	Erythropoietin	PI3K/AKT/mTOR signaling	↑ Intraplaque angiogenesis; potential plaque destabilization (pro‐atherogenic in this context)	E3	[34]
In silico only—Network pharmacology and molecular docking; no wet‐laboratory component	ERK signaling *nominated* as a candidate target	Calycosin	Predicted target–pathway network; docking scores	No experimental outcomes were measured. The compound is *nominated* for testing; no anti‐atherosclerotic effect is demonstrated	E5	[153]
Endothelial cells; atherosclerosis models	AKT (reduced phosphorylation)	Icariin, via miR‐205‐5p/ERBB4	miR‐205‐5p/ERBB4/AKT signaling	↓ Endothelial dysfunction; reduced lesion development (protective)	E3	[152]
C. Endothelial integrity and vascular remodeling						
Endothelial cells; angiotensin II‐induced dysfunction	K_Ca_2.3 channel (AMPK‐mediated phosphorylation)	AMPK	Endothelial hyperpolarization; restored NO bioavailability	Reversal of angiotensin II‐induced endothelial dysfunction (protective)	E3	[132]
Diet‐induced obese mice; endothelium	K_Ca_2.3 channel—*Upregulation of channel expression*, not phosphorylation	AMPK	Increased K_Ca_2.3 abundance; endothelium‐dependent hyperpolarization	Improved endothelial function in obesity (protective)	E3	[133]
Porcine aortic phosphoproteomics (disturbed vs. pulsatile flow regions); transgenic mice; human coronary biopsies and sera	Vinculin Ser721 (VCL^S721p^)	GRK2, itself phosphorylated at Ser29	VCL^S721p^ locks vinculin in an inactive closed conformation → disruption of the VE‐cadherin/catenin complex → increased endothelial permeability	↑ Permeability and atherogenesis. Serum sVCL^S721p^ correlated with atherosclerotic stage and was lower in statin‐treated patients (pro‐atherogenic)	E3‡ with E2 support	[129]
Endothelial cells exposed to oxLDL	ERK5 (increased phosphorylation)	Silymarin	Upregulation of ERK5‐dependent antioxidant defense	Increased resistance to oxLDL‐induced endothelial dysfunction (protective)	E4	[54]
Vascular endothelial cells exposed to oxLDL	p38 MAPK (reduced phosphorylation)	miR‐497‐5p, targeting VEGFA	VEGFA/p38/MAPK pathway	↓ Inflammatory angiogenesis and endothelial dysfunction (protective)	E4	[37]
Myeloid‐specific LXRα S196A knock‐in mice on an *Ldlr* ^−/−^ background, Western diet	LXRα Ser196 (phosphorylation disrupted by knock‐in substitution)	Kinase not identified in this study	Reprogrammed macrophage transcriptome; induction of FoxM1 and its targets; altered expression of phagocytic molecules	↑ Plaque burden with ↑ lesion‐resident cell proliferation, but *smaller necrotic cores and thinner fibrous caps* and enhanced efferocytosis, a mixed, not uniformly pro‐atherogenic, phenotype	E3‡	[135]
D. Phosphatase‐mediated control of lipid metabolism and inflammation						
Endothelial oxLDL stimulation; atherosclerosis‐related dyslipidaemia model	ERK/p38 (increased phosphorylation)	PPP2CB (PP2A catalytic subunit β); LOX‐1; ROS	LOX‐1/MAPK/ERK signaling	↑ Adhesion and inflammation; aggravated dyslipidemia and plaque development (pro‐atherogenic)	E3	[156]
Endothelial cells exposed to oxLDL	MAPK/ERK (attenuated by PP2A)	PP2A activation	LOX‐1/ROS/MAPK axis	Restored endothelial function; ↓ inflammatory tone (protective)	E4	[157]
Macrophage PP2A‐deficient mice	NF‐κB and MAPK (loss of phosphatase restraint)	PP2A deficiency	↓ ABCA1/ABCG1‐dependent cholesterol efflux; ↑ lipid storage	↑ Foam‐cell formation; accelerated lesion development (pro‐atherogenic)	E3	[158]
Myeloid‐specific PTP1B‐knockout mice	AMPK (increased activity)	PTP1B deletion	AMPK‐dependent cholesterol homeostasis	Improved cholesterol handling; ↓ inflammatory activation; protection against atherosclerosis (protective)	E3	[159]

*Note:* **Evidence grades** (Section 5). E1, human causal evidence; E2, human observational evidence; E3, in vivo perturbation of a defined enzyme in an animal model with a vascular outcome; E4, in vitro mechanistic evidence with a defined enzyme and substrate; E5, associative or computational evidence without functional validation. ‡, acceptor residue mapped and tested by substitution; may be appended to any grade. †, source has not completed peer review; no entry in this table carries†.

**Table 4:** Acetylation‐ and deacetylation‐dependent mechanisms in atherosclerosis.

Study system	Acetylation target (residue/domain where mapped)	Writer, eraser, or regulator	Mechanism and functional outcome	Direction	Evidence	References
A. Chromatin‐level acetylation and pharmacological HDAC inhibition						
Human carotid endarterectomy specimens graded by lesion severity	Global histone acetylation and methylation marks (H3K9ac, H3K27me3 and others), measured by immunohistochemistry	Not determined—The study measures marks, not enzymes	In human carotid endarterectomy specimens, H3K9ac and H3K27me3 levels varied with lesion severity (E2). These findings are correlative and do not establish causal enzymes, target genes, or downstream adhesion effects. Whether HDAC9‐dependent deacetylation overlaps functionally with H3K27me3 deposition by modifiers such as EZH2 remains untested in vascular tissue.	*Observational*	E2	[160]
Hypercholesterolemia ApoE^−/−^ mice	Global histone deacetylation (class‐nonselective pharmacological inhibition)	HDAC inhibitors	HDAC inhibition reduced NADPH oxidase expression and oxidative stress and slowed progression of atherosclerotic lesions	Protective	E3	[161]
ApoE^−/−^ mice; macrophages	PPARγ (acetylation status modulated indirectly)	HDAC inhibitors	HDAC inhibition enhanced PPARγ activity, reducing foam‐cell formation and atherosclerosis	Protective	E3	[162]
B. HDAC9 as a disease node						
Human cohorts, Slovenian [144] and Chinese Han [145]	Not applicable (genotype–phenotype association)	HDAC9 variant rs2107595	rs2107595 is associated with advanced carotid atherosclerosis [144] and coronary artery disease susceptibility and lesion severity [145]. These are association studies; no acetylation target was measured and no functional assay was performed.	*Observational*	E2	[144, 145]
HEK293 cells; myeloid‐restricted CRE‐deletion mice; human carotid endarterectomy tissue	NLRP3 NACHT and LRR domains	HDAC9, controlled by a conserved cis‐regulatory element containing rs2107595	Deletion of the element upregulates HDAC9 and exacerbates atherosclerosis in mice; HDAC9 deacetylates NLRP3 to activate the inflammasome. *The effect is myeloid‐restricted: rs2107595 raises HDAC9 expression in human PBMCs but not in vascular cells.*	Pro‐atherogenic	E1/E3 with E2 support	[115]
Hyperlipidemic mice with bone‐marrow reconstitution; TMP195; macrophages and endothelial cells	IKKα and IKKβ	HDAC9	HDAC9 binds to and deacetylates IKKα/β, activating them and driving inflammatory responses in both macrophages and endothelial cells. TMP195 reduces lesion growth and increases fibrous cap thickness. *No VSMC data are reported in this study.*	Pro‐atherogenic	E3	[21]
Endothelial Hdac9 deletion; atherosclerosis models	Endothelial–mesenchymal transition program (acetylation target not mapped)	HDAC9	HDAC9 promotes endothelial–mesenchymal transition and an unfavorable plaque phenotype	Pro‐atherogenic	E3	[116]
C. Nonhistone acetylation targets						
Thrombin‐stimulated human endothelial cells	NF‐κB p65	CREB‐binding protein (CBP), a histone acetyltransferase	CBP‐dependent p65 acetylation is required for thrombin‐mediated endothelial activation; CBP knockdown blocks it	Pro‐atherogenic	E4	[52]
Macrophages; diabetic atherosclerosis models	ZKSCAN3	Nε‐carboxyethyl‐lysine acting through RAGE/LKB1/AMPK/SIRT1	Altered ZKSCAN3 acetylation disrupts macrophage autophagy and influences plaque stability	Pro‐atherogenic	E3	[50]
D. Sirtuin‐dependent deacetylation						
ApoE^−/−^ mice; vascular cells	PGC‐1α	SIRT1, activated pharmacologically by SRT1720	SIRT1‐dependent PGC‐1α deacetylation enhances mitochondrial function and *rescues the progression* of atherosclerosis The reported effect is attenuation of progression, not regression of established plaque	Protective	E3	[45]
Macrophages; atherosclerosis models	Not mapped—Pathway‐level readout	SIRT1	SIRT1 restrains the macrophage inflammatory response and regulates the TIMP3/ADAM17 pathway	Protective	E3	[46]
Atherosclerosis models	Not mapped—Autophagy readout	SIRT1 inhibition	SIRT1 inhibition promotes atherosclerosis through impaired autophagy, providing the loss‐of‐function counterpart to the activation data above	Protective (when intact)	E3	[163]
Human peripheral blood mononuclear cells [164]; patients with chronic and acute ischemic coronary disease [165]	Not applicable, SIRT1 *expression level*, not acetylation, was measured	miR‐138‐5p (correlative)	SIRT1 expression is inversely correlated with miR‐138‐5p in blood mononuclear cells [164], and SIRT1 levels differ across ischemic coronary syndromes [165]. These are correlations; miRNA‐mediated repression of SIRT1 in vascular cells has not been demonstrated.	*Observational*	E5; E2† [165]	[164, 165]
*Ldlr* ^−/−^ mice; macrophages	Not mapped—Polarization readout	SIRT2	SIRT2 decreases plaque formation by modulating macrophage polarization away from an inflammatory phenotype	Protective	E3	[51]
Diabetic endothelium	Not mapped—Mitophagy and transcytosis readouts	SIRT3	SIRT3‐mediated mitophagy *ameliorates* atherosclerosis by attenuating endothelial LDL transcytosis. Source is a preprint that has not completed peer review.	Protective	E3†	[166]
Coronary endothelium in diabetes	Not mapped	SIRT3	SIRT3 attenuates coronary atherosclerosis in diabetes by regulating endothelial cell function	Protective	E3	[167]
Vascular smooth muscle cells; vascular remodeling models	Mitochondrial protein acetylation, via acetyl‐CoA availability	SIRT3	SIRT3 represses vascular remodeling by reducing mitochondrial acetyl‐CoA accumulation in VSMCs	Protective	E3	[168]
Atherosclerosis models treated with natural‐product agents	Not mapped	Upstream agents of uncertain target selectivity: *Valeriana officinalis* extract via PGC‐1α/Sirt3/Epac1 [169]; melatonin via SIRT3/FOXO3α/ROS [170]	Both agents raise SIRT3 activity and reduce atherogenic readouts, but neither establishes a SIRT3‐specific mechanism and neither compound is selective	Protective	E3	[169, 170]
Sirt4‐deficient mice	NF‐κB/IκB pathway activity *Acetylation of these substrates by SIRT4 was not demonstrated*	SIRT4	Sirt4 deficiency promotes atherosclerosis by activating the NF‐κB/IκB/CXCL2/3 pathway	Protective (when intact)	E3	[171]
Endothelial cells	Not mapped, oxLDL uptake readout	SIRT4, regulated by the mechanosensor PIEZO1	PIEZO1‐regulated SIRT4 suppresses oxLDL uptake in endothelial cells	Protective	E4	[172]
HUVECs exposed to oxLDL	Not mapped, PI3K/Akt/NF‐κB readout	SIRT4	SIRT4 suppresses PI3K/Akt/NF‐κB signaling and attenuates oxLDL‐induced endothelial injury	Protective	E4	[173]
Sirtuin 6‐deleted ApoE^−/−^ mice	Not mapped—Endothelial function and lesion readouts	SIRT6	Deletion of SIRT6 accelerates endothelial dysfunction and atherosclerosis in mice. *Histone acetylation was not measured in this study.*	Protective (when intact)	E3	[174]
Obesity‐related atherosclerosis models	Not mapped	IL‐38 acting upstream of SIRT6	IL‐38 alleviates atherogenic responses via SIRT6/HO‐1 signaling	Protective	E3	[175]
Vascular ageing models	Keap1 (deacetylation, promoting Keap1–Nrf2 dissociation)	SIRT7, activated by GLSP	SIRT7‐mediated Keap1 deacetylation dissociates Keap1 from Nrf2 and mitigates vascular aging. *The outcome measured is vascular ageing, not atherosclerotic lesion burden.*	Protective	E3	[176]
Endothelial cells under oxidative stress	Not mapped, SIRT7 expression readout	H_2_O_2_ acting through microRNA‐335‐5p	H_2_O_2_ downregulates SIRT7 via miR‐335‐5p, removing its protection against premature endothelial dysfunction	Protective (when intact)	E4	[177]

*Note:* **Evidence grades** (Section 5). E1, human causal evidence; E2, human observational evidence; E3, in vivo perturbation of a defined enzyme in an animal model with a vascular outcome; E4, in vitro mechanistic evidence with a defined enzyme and substrate; E5, associative or computational evidence without functional validation. ‡, acceptor residue mapped and tested by substitution; may be appended to any grade. †, source has not completed peer review. **No entry meets the ‡ standard.** The only substrate in this table for which a specific acceptor region is identified is NLRP3, where HDAC9 acts within the NACHT and LRR domains rather than at individual mapped lysines. This is a substantive gap: Acetylation is among the best‐characterized modifications biochemically, yet the vascular literature remains almost entirely pathway‐level. **One entry carries †:** The SIRT3–mitophagy relationship, which rests on a preprint. **SIRT5 is omitted** because sufficient vascular mechanistic evidence was not identified, consistent with the legend to Figure 4.

**Table 5:** Ubiquitin ligases, deubiquitinases, and ubiquitin‐activating enzymes in atherosclerosis, grouped by enzyme class and direction of effect.

Enzyme (class)	Substrate (linkage/residue where mapped)	Study system	Mechanism and functional outcome	Direction	Evidence	References
A. E3 ubiquitin ligases with pro‐atherogenic function						
TRIM25 (RING E3)	XRCC1 (linkage not specified)	Macrophages; atherosclerosis models	TRIM25‐mediated XRCC1 ubiquitination accelerates atherosclerosis by inducing macrophage M1 polarization and programmed cell death. *TRIM25 also acts as an ISG15 E3 ligase* [142], *making it a shared enzyme across two conjugation systems (Section 7.6)*	Pro‐atherogenic	E3	[118]
ITCH (HECT E3)	Ferritin light chain (FTL)	Endothelium; atherosclerosis models	ITCH‐mediated ubiquitination of ferritin light chain releases labile iron and contributes to endothelial ferroptosis	Pro‐atherogenic	E3	[178]
TRIM21 (RING E3)	SOCS3	Endothelial cells; atherosclerosis models	TRIM21 accelerates SOCS3 ubiquitination and degradation, relieving inhibition of JAK/STAT signaling and promoting endothelial cell activation	Pro‐atherogenic	E3	[179]
RNF128 (RING E3)	SR‐B1—Lys63‐linked polyubiquitination	Macrophages; ApoE^−/−^ mice	RNF128 stabilizes rather than degrades SR‐B1, increasing oxLDL internalization and foam cell formation. Illustrates that the consequence of Lys63 linkage is set by substrate fate, not by linkage type alone.	Pro‐atherogenic	E3	[180]
B. E3 ubiquitin ligases with protective function						
Peli1 (RING E3)	Not mapped—No substrate identified in this study	Bone‐marrow and immune compartments; atherosclerosis models	*Peli1 deficiency*, rather than Peli1 activity, promotes atherosclerosis progression. Peli1 is therefore protective.	Protective (when intact)	E3	[181]
Listerin (RING E3)	ABCA1—Lys63‐linked polyubiquitination at Lys1884 and Lys1957	Macrophage‐specific Listerin‐knockout mice; THP‐1 cells; human and rodent plaque	Listerin stabilizes ABCA1 by counteracting ESCRT‐mediated lysosomal degradation induced by oxLDL. Macrophage‐specific deletion suppressed cholesterol efflux and exacerbated plaque features; overexpression attenuated these features; the ABCA1 agonist erythritol rescued the deficient phenotype, and ABCA1 knockout abolished the effect. *Listerin expression rises with plaque progression in humans and rodents.*	Protective	E3 with E2 support	[182]
SMURF2 (HECT E3)	HMGB1	Endothelial cells; atherosclerosis models	SMURF2 promotes HMGB1 degradation and inhibits endothelial inflammation, protecting against atherosclerosis	Protective	E3	[141]
C. Deubiquitinases (Section 6.3.2)						
USP20 (DUB)	β‐arrestin2 (TLR4 → NF‐κB axis)	Cell‐based mechanistic studies	USP20 regulates the reciprocal functions of β‐arrestin2 in TLR4‐promoted NF‐κB activation. *This study provides the mechanism only; it contains no in vivo atherosclerosis data.*	Protective	E4	[117]
USP20 (DUB)	Not mapped—TNF‐triggered inflammatory signaling	Vascular smooth muscle cells; atherosclerosis models	USP20 inhibits TNF‐triggered smooth muscle cell inflammation and attenuates atherosclerosis. This study provides in vivo evidence for USP20.	Protective	E3	[183]
USP11 (DUB)	CD36	Macrophages; atherosclerosis models	USP11‐mediated CD36 deubiquitination acts with C1q/TNF‐related protein 9 (CTRP9) against atherosclerosis. *Because CD36 is classically a driver of oxLDL uptake, the protective direction is counterintuitive and warrants independent confirmation.*	Protective	E4	[184]
USP22 (DUB)	PPARγ	Macrophages; atherosclerosis models	USP22 stabilizes PPARγ, enhancing macrophage efferocytosis and plaque stability. *PPARγ is also acetylation‐regulated (*Table 4 *), making it a candidate site of acetylation–ubiquitination cross‐talk that has not been tested in vascular cells (Section 7.3).*	Protective	E3	[185]
USP18 (DUB)	ABCG1 (*reported*)	Macrophages; atherosclerosis models	USP18 deubiquitinates ABCG1 and promotes cholesterol efflux. Caveat: USP18 specifically removes ISG15 from conjugated proteins and its activity toward ubiquitin conjugates is undetectable in vitro [186], so an ISG15‐directed rather than ubiquitin‐directed mechanism cannot be excluded (Section 7.6)	Reported Protective	E4 (mechanism contested)	[137]
USP53 (DUB)	SR‐A	Smooth muscle cells; endothelial–VSMC co‐culture; atherosclerosis models	Endothelial Dickkopf‐1 promotes *smooth muscle cell‐derived* foam cell formation via USP53‐mediated deubiquitination and stabilization of SR‐A.	Pro‐atherogenic	E3	[53]
D. Ubiquitin activation and global flux						
UBA1 (E1 activating enzyme)	Global ubiquitin conjugation, no individual substrate	ApoE^−/−^ mice; PYR‐41	Small‐molecule UBA1 inhibition suppressed diet‐induced atherosclerosis without measurable changes in systemic lipid metabolism or immune cell composition. *Whether protective immune surveillance is preserved over longer exposures has not been tested.*	Protective (inhibition)	E3	[146]
E. Non‐anonical routes to proteasomal degradation						
circBTBD7‐420aa (circRNA‐encoded protein)	SLC3A2	VSMCs; ApoE^−/−^ mice; engineered exosomes	A protein encoded by hsa*circ*0000563 promotes ubiquitin–proteasome‐mediated degradation of SLC3A2, reducing VSMC proliferation and migration	Protective	E3	[187]
SCF(FBXO24) (cullin‐RING E3)	FoxP1	Non‐vascular cell systems	Reciprocal regulation between SCF(FBXO24) and FoxP1. *The work is a preprint work is a preprint and no vascular phenotype is reported; extrapolation to atherosclerotic remodeling is not supported.*	*Not established*	E4†	[188]

*Note:* **Evidence grades** (Section 5). E1, human causal evidence; E2, human observational evidence; E3, in vivo perturbation of a defined enzyme in an animal model with a vascular outcome; E4, in vitro mechanistic evidence with a defined enzyme and substrate; E5, associative or computational evidence without functional validation. ‡, acceptor residue mapped and tested by substitution; may be appended to any grade. †, source has not completed peer review. **One entry meets the ‡ standard:** Listerin‐mediated stabilization of ABCA1 at Lys1884 and Lys1957. **One entry carries †:** The SCF^FBXO24^–FoxP1 relationship, which rests on a preprint and reports no vascular phenotype. **Direction of effect** in this table is set by substrate identity and cellular context rather than by enzyme class or ubiquitin‐linkage type; RNF128 and Listerin both catalyse Lys63‐linked polyubiquitination with opposite consequences, and USP53 is a deubiquitinase with a pro‐atherogenic effect. **Contested entry.** The USP18–ABCG1 entry is retained with an explicit caveat because the reported ubiquitin‐directed mechanism is difficult to reconcile with the established ISG15 specificity of USP18 (Section 7.6).

**Table 6:** ADP‐ribosylation‐dependent mechanisms in atherosclerosis.

Enzyme or node	Target/axis	Study system	Mechanism and functional outcome	Direction	Evidence	References
A. PARP1‐driven smooth muscle phenotype and calcification						
PARP1 (PARylation)	STAT1 → Runx2	VSMCs; diabetic arteriosclerotic calcification models	Loss of PARP‐1 attenuates diabetic arteriosclerotic calcification via the Stat1/Runx2 axis PARP1 activity therefore supports osteogenic conversion at the expense of the contractile program	Pro‐atherogenic	E3	[126]
PARP1 (PARylation)	TET1 (5‐hydroxymethylcytosine)	Atherosclerotic VSMCs	Activated PARP1 upregulates TET1 and drives a contractile‐to‐synthetic phenotypic switch. The shift was reversible on PARP1 inhibition within the timeframe studied	Pro‐atherogenic	E4	[127]
PARP1 (PARylation)	Myocardin and serum response factor	VSMCs; arterial disease models	PARP1 orchestrates VSMC homeostasis: PARylation of myocardin and SRF disrupts the CArG‐box transcriptional complex, releasing the contractile program, and permitting proliferation and migration. SRF is also SUMOylated with opposite consequences, making it a shared substrate of two systems (Section 7.6).	Pro‐atherogenic	E3	[128]
PARP1 (PARylation)	NAD^+^ pool (no protein substrate identified)	Aortic VSMCs in a mouse model of Hutchinson–Gilford progeria	Excess PARP1 activity depletes NAD^+^ and drives VSMC loss; the PARP1 inhibitor trifluridine replenished NAD^+^, preserved VSMC viability and improved aortic disease	Pro‐atherogenic	E3	[47]
B. PARP1 in the endothelium and immune compartments						
PARP1 (upregulated by NRP2)	Endothelial apoptosis program	Endothelium at low‐shear regions; atherosclerosis models	NRP2 promotes atherosclerosis by upregulating PARP1 expression and enhancing low shear stress‐induced endothelial apoptosis	Pro‐atherogenic	E3	[189]
PARP1 (inhibited by INO‐1001)	Dendritic cell and T‐cell activation; autoantibody production	ApoE^−/−^ mice	PARP inhibition reduced dendritic cell and T‐cell infiltration and inflammatory mediator levels. Two cautions: The study also reports reduced anti‐oxLDL IgM, and natural IgM against oxLDL is generally considered atheroprotective, so the net immunological effect is not uniformly favorable; and this study does not demonstrate PARylation of any NF‐κB component, so its use as evidence for ADP‐ribosylation at that node (Table 2) is inferential	Protective (inhibition)	E3	[143]
C. PARP1 in lipid handling and bile‐acid metabolism						
PARP1 (inhibited by 3‐aminobenzamide)	FXR/BSEP; bile salt hydrolase‐expressing gut bacteria	Hepatic and gut compartments in atherosclerosis models	PARP‐1 inhibition alleviates hepatic lipid accumulation by modulating bile acid metabolism and the gut microbiota. The readout is hepatic and metabolic; a direct effect on plaque was not the primary endpoint.	Protective (inhibition)	E3	[190]
PARP1 (suppressed by geniposide)	PARP1/PI3K/AKT → lipophagy	Macrophages; atherosclerosis models	Geniposide ameliorates atherosclerosis by restoring lipophagy through suppression of PARP1/PI3K/AKT signaling; the effect was reversed by PARP1 overexpression, which is the epistatic control that makes the attribution to PARP1 credible	Protective (inhibition)	E3	[191]
D. Cofactor coupling with sirtuin deacylation						
NAD^+^ metabolism → PARP1/CD38	Shared NAD^+^ pool	ApoE^−/−^ mice given NAD^+^ precursors	NAD^+^ boosting increased atherosclerotic plaque area and inflammation, with CD38 upregulation and redirected NAD + consumption toward PARP1 rather than sirtuins. The only in vivo vascular evidence that the interaction between the two enzyme systems, rather than either alone, sets the phenotype.	Pro‐atherogenic	E3	[48]
E. Other ADP‐ribosyltransferases, limited evidence						
PARP14 (mono‐ADP‐ribosyltransferase)	Ferroptosis‐associated gene signature	Public transcriptomic data sets for diabetic retinopathy and atherosclerosis	The diverse pro‐ and anti‐atherogenic roles of ADP‐ribosylation across vascular, immune, and metabolic compartments, including PARP1‐driven remodeling, computational nomination of PARP14 as a ferroptosis‐associated candidate (E5, unvalidated), and context‐dependent modulation of lipid metabolism, are consolidated in Table 6.	Not established	E5	[192]
Unidentified ADP‐ribosyltransferase(s)	Apolipoproteins (APOA1, APOE)	Mouse model of atherosclerosis; lipoprotein fractions	ADP‐ribosylation of apolipoproteins is reported to increase in lesions and be associated with impaired cholesterol efflux. Conference abstract without full publication; the responsible enzyme is not identified and the quantitative claims cannot be verified from the abstract	Not established	E3†	[193]
F. ARF‐family GTPases, named for, but not catalysts of, ADP‐ribosylation						
ARL11 (ADP‐ribosylation factor‐like protein 11), a small GTPase, not an ADP‐ribosyltransferase	JAK2/STAT1	Macrophages; Apoe^−/−^ mice with ARL11 silencing	Silencing ARL11 relieved atherosclerotic inflammation and lipid deposition by restraining the JAK2/STAT1 signaling pathway. The mechanism does not involve ADP‐ribosylation.	Pro‐atherogenic	E3	[194]
ARF6 (ADP‐ribosylation factor 6), a small GTPase, not an ADP‐ribosyltransferase	Lipid uptake and inflammatory signaling	ARF6‐heterozygous atherosclerosis‐prone mice	Heterozygosity for ARF6 suppresses the burden and severity of atherosclerosis; therefore, ARF6 activity is pro‐atherogenic.	Pro‐atherogenic	E3	[195]

*Note:* ^a^ **ARL11 and ARF6 are not ADP‐ribosyltransferases.** The ARF family is named for the discovery of these proteins as cofactors for cholera‐toxin‐catalyzed ADP‐ribosylation of Gαs; the proteins themselves are small GTPases and neither catalyse nor accept ADP‐ribosylation in the studies cited. They are retained in a separately labeled block, rather than omitted, because the shared nomenclature has repeatedly led to their being grouped with this modification class; they should not be read as ADP‐ribosylation mechanisms. **Evidence grades** (Section 5). E1, human causal evidence; E2, human observational evidence; E3, in vivo perturbation of a defined enzyme in an animal model with a vascular outcome; E4, in vitro mechanistic evidence with a defined enzyme and substrate; E5, associative or computational evidence without functional validation. ‡, acceptor residue mapped and tested by substitution; may be appended to any grade. †, source has not completed peer review. **Human evidence is lacking in this table.** No entry reaches E1 or E2. Every mechanistic claim rests on rodent models, cell culture, or computational analysis, which is why the NAD^+^‐dependent deacylation–PARylation axis is admitted to Tier 1 in Table 2 by stated exception on the human‐anchoring criterion rather than by meeting it.

**Table 7:** Inventory of posttranslational modification cross‐talk relationships invoked in this review, classified by mechanistic category and graded for the type and strength of supporting evidence.

Cross‐talk relationship	Category	Substrate and acceptor residue	Experimental system and principal finding	Evidence grade	Vascular?	References
A. Cofactor‐coupled cross‐talk						
Sirtuin‐mediated deacylation ↔ PARP1‐mediated ADP‐ribosylation	Cofactor‐coupled	No shared substrate; competition for the NAD^+^ pool	*Apoe*‐knockout mice, in vivo. NAD + supplementation increased plaque area and inflammation, with CD38 upregulation and NAD + redirected toward PARP1 rather than sirtuins [48]. Sirtuin [45, 46] and PARP1 [44, 47] NAD + dependence was established separately.	E3	Yes	[44, 45, 46, 47, 48]
B. Competitive and hierarchical cross‐talk on serine and threonine						
O‐GlcNAcylation ↔ phosphorylation	Competitive — demonstrated	eNOS Ser1177 (Akt site)	Bovine aortic endothelial cells and aortae of diabetic rats were used. Hyperglycemia increased eNOS O‐GlcNAc modification and reciprocally decreased Ser1177 phosphorylation; both changes were reversed by GFAT antisense oligonucleotides or by UCP‐1 or MnSOD overexpression; glycosylated eNOS had lower activity; eNOS mutated at the Akt site showed no hyperglycemia‐induced change in O‐GlcNAc.	E4‡	Yes	[196]
O‐GlcNAcylation ↔ phosphorylation	Competitive, inferred (residues not mapped)	β‐catenin; acceptor residues not mapped in VSMCs	Smooth muscle‐restricted *Ogt* deletion in high‐phosphate and chronic kidney disease models [19] and in hyperglycemic *Apoe*‐deficient mice [120]. OGT has been reported to increase β‐catenin stability and Wnt‐driven osteogenic conversion; however, competition with phosphorylation was not tested, and no phosphorylation‐site mutant was examined	E3 for the phenotype; no residue‐level cross‐talk data	Yes (VSMC)	[19, 120]
O‐GlcNAcylation ↔ phosphorylation	Hierarchical—Proposed, untested	Runx2; AKT	Calcifying VSMCs with increased O‐GlcNAcylation induced by thiamet‐G or OGA knockdown. AKT O‐GlcNAcylation at Thr430/Thr479 enhanced Ser473 phosphorylation and Runx2 transactivity; combined mutation of both residues blocked Runx2 activation and calcification	E3†	Yes	[138]
C. Competitive and hierarchical cross‐talk on lysine and at NF‐κB						
SUMOylation ↔ ubiquitination	Competitive—Demonstrated	IκBα Lys21	Nonvascular cell systems in vitro. Ubc9 conjugated SUMO‐1 to IκBα primarily at Lys21, the residue also used for ubiquitination; SUMO‐modified IκBα could not be ubiquitinated and was resistant to proteasome‐mediated degradation, and SUMO‐1 overexpression inhibited signal‐induced NF‐κB‐dependent transcription	E4‡	No	[197]
Phosphorylation → ubiquitination; phosphorylation ⊣ SUMOylation	Hierarchical—Demonstrated	IκBα Ser32/Ser36	Nonvascular cell systems in vitro. Ubiquitination of IκBα requires Ser32/Ser36 phosphorylation, whereas SUMO‐1 modification of IκBα is inhibited by the same phosphorylation.	E4‡	No	[197]
Phosphorylation → acetylation	Hierarchical—Demonstrated	NF‐κB RelA/p65: Ser536 (and Ser276) → Lys310	RelA‐deficient mouse embryonic fibroblasts, transfected cells, and TNF‐α stimulation. Phosphorylation at Ser276 or Ser536 increased the assembly of p65 with p300 and enhanced Lys310 acetylation. Reconstitution with S536A reduced TNF‐α‐induced Lys310 acetylation and expression of the NF‐κB target gene E‐selectin.	E4‡	No	[198]
ISGylation ⊣ phosphorylation	Enzyme‐directed (phosphatase recruitment)	NF‐κB p65; ISGylation acceptor residues not reported	Endothelial cells and murine models of acute lung injury, *not* atherosclerosis. SCF(FBXL19)‐mediated ISGylation of p65 reported to stabilize the p65–WIP1 interaction and reduce inflammatory serine phosphorylation and nuclear translocation; FBXL19 knockdown increased and endothelial FBXL19 overexpression decreased inflammation in vivo [124]. WIP1 is a direct phosphatase of p65 Ser536 and reduces p65–p300 binding [199].	E3/E4	Endothelial, but non‐atherosclerotic model	[124, 199]
ISGylation ⊣ phosphorylation → acetylation	Hierarchical—Hypothesis generated in this review; not tested	NF‐κB p65 (Ser536 → Lys310)	No experimental system. The prediction follows from the two preceding rows combined; no study has measured p65 Lys310 acetylation after manipulation of FBXL19 or ISG15 in any cell type.	Not tested	Not applicable	[124, 198, 199]
SUMOylation ↔ ubiquitination	Competitive—Inferred; acceptor lysines not examined for ubiquitin occupancy	HIF‐1α [43]; NLRP3 [38]; TFEB [200]; SRF [139]; SERCA2a [201]	Macrophages and VSMCs, cultured cells, and mouse models. CBX4‐dependent SUMOylation of HIF‐1α reported to increase transcriptional activity and lesion burden [43]; SENP3‐dependent deSUMOylation of NLRP3 reported to restrain inflammasome activation [38]; TFEB SUMOylation reported to suppress lysosomal activity and promote foam cell formation [200]; SRF SUMOylation reported to promote phenotypic switching [139]; SUMO‐1 conjugation of SERCA2a reported to preserve Ca^2+^ handling under diabetic stress [201].	E3/E4 for each modification; no cross‐talk data	Yes	[38, 43, 139, 200, 201]
SUMOylation, residue‐resolved (competition with ubiquitin not tested)	Competitive—SUMO site mapped and tested	LRH‐1 Lys289 (K289R)	Hepatic LRH‐1 in hypercholesterolemic mice. K289R abolished the LRH‐1–PROX1 corepressor interaction, increased reverse cholesterol transport and reduced lesion burden. Whether Lys289 also accepts ubiquitin was not examined.	E3‡	Systemic/hepatic, not vascular‐cell‐autonomous	[20]
Deacetylation ↔ deSUMOylation	Competitive versus parallel—Undetermined	NLRP3: NACHT and LRR domains for deacetylation [115]; SUMO2/3 acceptor sites not mapped [38]	Macrophages, mouse models, and human carotid endarterectomy tissue [115]. HDAC9‐mediated deacetylation is reported to permit inflammasome activation and pyroptosis; SENP3‐mediated deSUMOylation is reported to restrain inflammasome activation and foam cell formation. The site overlap between the two lysine marks has not been determined.	E3 and E3	Yes	[38, 115]
Acetylation ↔ ubiquitination	Chemically obligate at shared lysines; not examined in the cited vascular studies	PPARγ; acetylation status not measured	Macrophages, cultured cells, and mouse models. USP22‐dependent deubiquitination has been reported to stabilize PPARγ and support efferocytosis and plaque stability [185]; NEDD4L‐dependent ubiquitination of IκBα and PPARγ has been reported to drive M1 polarization and foam cell formation [202]; NEDD4L knockdown suppressed PPARγ ubiquitination and increased ABCA1 and ABCG1 [203]. No cited study has measured PPARγ acetylation.	E3/E4 for ubiquitination only	Yes	[185, 202, 203]
D. Enzyme‐directed cross‐talk (modification of writers, erasers and readers)						
Deacetylation → phosphorylation, and deacetylation → inflammasome effector	Enzyme‐directed	IKKα/β (writer of the phosphorylation arm) [21]; NLRP3 [115]	Macrophages and endothelial cells; hyperlipidemic mice. HDAC9 deacetylates IKKα/β and activates IKK, increasing inflammation and reducing fibrous cap thickness [21]; HDAC9 also deacetylates NLRP3 [115]. Therefore, a single class IIa deacetylase acts on both an upstream kinase and a downstream effector of the same axis.	E3	Yes	[21, 115]
Phosphorylation ⊣ SUMO conjugating machinery	Enzyme‐directed	UBC9 (SUMO E2); downstream substrate GPR120	AMPKα2‐deficient mice and human arterial specimens. AMPKα2 activity repressed UBC9 and limited GPR120 SUMOylation, and was required for the anti‐atherosclerotic effect of ω−3 fatty acids. AMPKα2 ablation produced GPR120 hyper‐SUMOylation and abolished the benefit. The human data comprise inverse AMPKα2–UBC9 correlations and clinical subgroup analyses, and are correlative.	E3 (mouse); E2, correlative (human)	Yes	[204]
SUMOylation → phosphorylation	Enzyme‐directed	HSP90AB1; downstream ERK1/2	Human monocytes in vitro. Chemerin acted through β2‐integrin to trigger global SUMO2/3 conjugation, SUMOylated HSP90AB1, and was associated with ERK1/2 activation and a pro‐adhesive state; effects were reversed by the SUMO pathway inhibitor ML‐792.	E4	No—Circulating human monocytes, not vascular wall cells	[205]
Phosphorylation cascade → deacetylation	Enzyme‐directed	ZKSCAN3 acetylation, downstream of RAGE/LKB1/AMPK/SIRT1	Macrophages and models of diabetes atherosclerosis. Nε‐carboxyethyl‐lysine dysregulated RAGE/LKB1/AMPK/SIRT1 signaling was reported to inhibit ZKSCAN3 acetylation‐regulated macrophage autophagy and promote plaque instability.	E3	Yes	[50]
S‐palmitoylation → phosphorylation	Enzyme‐directed	PI3K p110α (Cys residues not specified); downstream AKT–mTOR	Zdhhc1‐deficient macrophages and *ApoE*‐deficient mice. Zdhhc1 deficiency reduced p110α palmitoylation, facilitated its nuclear translocation, suppressed AKT–mTOR signaling, and reduced oxLDL uptake, foam cell formation, and plaque burden.	E3	Yes	[121]
Ubiquitination → ADP‐ribosylation	Enzyme‐directed	XRCC1; downstream PARP1 activity	Macrophages and mice. TRIM25‐mediated ubiquitination of XRCC1 was reported to release PARP1 activity and induce M1 polarization and programmed cell death; TRIM25 loss reduced the lesion burden and fibrosis. This is the only documented ubiquitination–ADP‐ribosylation link in the vascular literature.	E3	Yes	[118]
E. Structural non‐independence: Shared enzymes and shared substrates						
Ubiquitin and ISG15 systems share an E3 ligase	Structural—Shared enzyme	TRIM25/EFP (ubiquitin E3 and ISG15 E3; ISGylates 14‐3‐3σ)	Enzymology of nonvascular cells in vitro [142]. The atheroprotective phenotype of TRIM25 loss in mice [118] was attributed to ubiquitination, but the contribution of ISGylation was not excluded.	E4 (enzymology); E3 (vascular phenotype)	Enzymology no; phenotype yes	[118, 142]
Ubiquitin and ISG15 systems share an E2 enzyme	Structural—Shared enzyme	UBE2L6/UbcH8	Porcine model of combined hyperglycemia and hyperlipidemia; UBE2L6 and MX1 identified as network centrality nodes by transcriptomic and protein–protein interaction analysis [206]. UbcH8 is a common component of the ubiquitin and ISG15 conjugation cascades [142]; therefore, network centrality cannot be assigned to a single modification.	E5 (computational/associative) plus E4 (enzymology)	Yes (associative)	[142, 206]
Ubiquitin‐ versus ISG15‐directed activity of USP18—Discrepancy unresolved	Structural—Shared enzyme	ABCG1	USP18 promotes cholesterol efflux and mitigates atherosclerosis by deubiquitinating ABCG1 in macrophages and mice [137]. In enzymological studies, USP18/UBP43 specifically removes ISG15 from conjugated proteins, with activity toward ubiquitin fusions undetectable in vitro [186]; USP18 also acts as an ISG15 E3‐pathway component alongside UbcH8 and EFP [142]. This discrepancy has not been resolved, and ISG15 independently promotes ABCA1‐dependent efflux [123].	E3 [137] versus E4 [186]	Vascular versus nonvascular	[123, 137, 142, 186]
Three modification systems converge on SRF	Structural—Shared substrate	SRF: PARylation [128] and SUMOylation [139]. *Ogt* deletion reduces SRF expression [120], O‐GlcNAcylation of SRF itself was not demonstrated	VSMCs and mice. PARP1 poly(ADP‐ribosylates) myocardin and SRF, dismantling the CArG‐box complex [128]; SRF SUMOylation modulates the contractile‐to‐synthetic switch and matrix deposition [139]; smooth muscle *Ogt* deletion reduced SRF and YY1 expression [120]. No study has manipulated more than one modification at a time.	E3 for each modification individually	Yes	[120, 128, 139]
Multiple systems converge on cholesterol‐efflux transporters	Structural—Shared substrate	ABCA1 [123, 182, 203]; ABCG1 [137, 203]	Macrophages, THP‐1‐derived foam cells, and mice. Listerin‐mediated Lys63‐linked ubiquitination reported to stabilize ABCA1 and support efflux [182]; USP18 reported to stabilize ABCG1 [137]; NEDD4L knockdown increased ABCA1 and ABCG1 [203]; ISG15 promoted ABCA1‐mediated efflux via miR‐17‐5p/Beclin‐1‐dependent autophagy [123]. Whether these constitute one regulatory module or independent mechanisms remains unresolved.	E3/E4	Yes	[123, 137, 182, 203]

*Note:* **Evidence grades** (Section 5). E1, human causal evidence; E2, human observational evidence; E3, in vivo perturbation of a defined enzyme in an animal model with a vascular outcome; E4, in vitro mechanistic evidence with a defined enzyme and substrate; E5, associative or computational evidence without functional validation. ‡, acceptor residue mapped and tested by substitution; may be appended to any grade. †, source has not completed peer review. **The “Vascular?” column** records whether the supporting evidence was obtained in vascular cells or tissues, independently of grade. **Residue resolution.** Of the 24 relationships inventoried, five are supported at residue resolution by a substitution experiment, occupying six rows because the two IκBα relationships share one substrate. Two of the five, the eNOS Akt site [191] and AKT Thr430/Thr479 [140], were obtained in vascular cells; the IκBα [196] and p65 [197] relationships were established in nonvascular systems, and the LRH‐1 result [20] is hepatic and systemic. **Categories.** Rows in blocks A–D are assigned to the four cross‐talk categories defined in Section 7. Block E records structural nonindependence, in which two modification systems share an enzyme or a substrate without any dependency having been demonstrated; this is not cross‐talk in the sense defined here and is listed separately. **One row states a prediction** generated in this review that has not been tested in any system, and is labeled accordingly.

**Table 8:** Interventions directed at PTM pathways in atherosclerosis, with the stage of development stated for each.

Target/node	Intervention	Mechanism and reported effect	Study system	Development stage	Evidence	References
A. Acetylation and deacetylation						
Class IIa HDACs, including HDAC9	TMP195, a class IIa HDAC inhibitor; also delivered in nanoparticle formulation	Deacetylation of IKKα/β is blocked, reducing IL‐1β and IL‐6 production and leukocyte recruitment, slowing lesion growth, and increasing fibrous cap thickness. TMP195 inhibits class IIa HDACs. No HDAC9 isoform‐selective inhibitor has been reported, and the atheroprotective effect cannot be attributed to HDAC9 alone.	Hyperlipidemic mice	Agent tested in vivo	E3	[21, 115]
SIRT1	SRT1720 (activator)	Restores PGC‐1α deacetylation and mitochondrial function; rescues progression of atherosclerosis	ApoE^−/−^ mice; vascular cells	Agent tested in vivo	E3	[45]
SIRT3	Valeriana officinalis extract; melatonin	Both increase SIRT3 activity and reduce atherogenic readouts through the PGC‐1α/Sirt3/Epac1 and SIRT3/FOXO3α/ROS axes. Neither compound is selective for SIRT3 and neither study establishes a SIRT3‐specific mechanism.	Rodent and cell‐based models	Agent tested in vivo (uncertain selectivity)	E3	[169, 170]
SIRT6	IL‐38 (recombinant cytokine)	SIRT6/HO‐1 signaling alleviates atherogenic endothelial responses	Endothelial cells; obesity‐related models	Agent tested in vitro only	E4	[175]
SIRT7	Ganoderma lucidum spore powder (GLSP)	GLSP up‐regulates SIRT7, which deacetylates Keap1 and promotes Keap1–Nrf2 dissociation and Nrf2 activation, mitigating vascular ageing and VSMC calcification. Atherosclerotic lesion burden was not the measured endpoint.	Aged C57BL/6 J mice; senescent VSMCs	Agent tested in vivo	E3	[176]
B. Phosphorylation						
NF‐κB and p38/ERK MAPK	Fucosterol	Suppresses NF‐κB and p38/ERK phosphorylation; reduces cytokine production and foam‐cell formation	Macrophages; atherosclerosis models	Agent tested in vivo (uncertain selectivity)	E3	[36]
p38 MAPK → ATF‐1	Calycosin‐7‐glucoside	Limits oxLDL‐induced foam cell formation via p38/MAPK‐dependent ATF‐1 activation. This strategy is pathway redirection rather than p38 inhibition.	THP‐1‐derived macrophages	Agent tested in vitro only	E4	[136]
MAPK1/ERK2	Resveratrol	Modulates MAPK1‐dependent ferroptosis, reducing lipid peroxidation and inflammatory injury	Vascular cells	Agent tested in vivo (uncertain selectivity)	E4	[151]
AMPK → K_Ca_2.3	AMPK activation (pharmacological and metabolic)	AMPK phosphorylates K_Ca_2.3, restoring endothelium‐dependent hyperpolarization and NO bioavailability in angiotensin II‐induced dysfunction [132]; in diet‐induced obesity AMPK upregulates channel expression rather than phosphorylating it [133]	Angiotensin II‐treated and diet‐induced obese mice	Agent tested in vivo	E3	[132, 133]
C. Ubiquitination						
UBA1 (E1 activating enzyme)	PYR‐41	It suppresses global ubiquitin activation, lowers adhesion molecules and macrophage cytokines, and reduces plaque development without altering systemic lipids or immune cell composition. Long‐term effects on protective immune surveillance have not been tested.	ApoE^−/−^ mice	Agent tested in vivo	E3	[146]
SLC3A2	circBTBD7‐420aa delivered in engineered exosomes	Promotes ubiquitin–proteasome‐mediated SLC3A2 degradation, suppressing VSMC proliferation and migration	VSMCs; ApoE^−/−^ mice	Agent tested in vivo	E3	[187]
ABCA1 stabilization via Listerin	None, target nomination	Listerin stabilizes ABCA1 by lys63‐linked polyubiquitination at Lys1884/Lys1957, thereby countering oxLDL‐induced lysosomal degradation. Macrophage‐specific deletion worsened and overexpression attenuated disease. An agent that promotes this stabilization has not been reported; the ABCA1 agonist erythritol was used as a tool compound.	Macrophage‐specific knockout mice; THP‐1 cells	Genetic validation only—No agent reported	E3	[182]
D. ADP‐ribosylation						
PARP1	Trifluridine	Replenishes NAD^+^, preserves VSMC viability and improves aortic disease	Progeroid mouse aorta	Agent tested in vivo	E3	[47]
PARP1	3‐Aminobenzamide	It upregulates FXR/BSEP and reduces bile salt hydrolase‐expressing gut bacteria, thereby lowering hepatic lipid accumulation. The endpoint is hepatic and metabolic rather than a direct plaque measurement.	Hepatic and gut compartments in atherosclerosis models	Agent tested in vivo	E3	[190]
PARP1/PI3K/AKT	Geniposide	Restores lipophagy and reduces foam‐cell formation; the effect was abolished by PARP1 overexpression, an epistatic control supporting the attribution	Apoe^−/−^ mice; macrophages	Agent tested in vivo (uncertain selectivity)	E3	[191]
PARP1	INO‐1001	It reduces dendritic cell and T‐cell infiltration and inflammatory mediators. The same study reports reduced anti‐oxLDL IgM; natural IgM against oxLDL is generally considered atheroprotective, so the net immunological effect is not uniformly favorable.	Apoe^−/−^ mice	Agent tested in vivo	E3	[143]
E. SUMOylation						
NLRP3 deSUMOylation	None, SENP3 gain‐of‐function used as genetic tool	SENP3 removes SUMO2/3 from NLRP3, restricting oxLDL‐induced inflammasome activation and foam‐cell formation. No SENP3‐activating agent has been reported.	Macrophages; mice	Genetic validation only, no agent reported	E3	[38]
CBX4 (SUMO E3 ligase)	None, target nomination for inhibition	Macrophage CBX4 SUMOylates HIF‐1α and potentiates atherosclerosis, nominating CBX4 as a potential therapeutic target. No CBX4 inhibitor has been reported.	Macrophages; atherosclerosis models	Genetic validation only, no agent reported	E3	[43]
F. O‐GlcNAcylation						
OGT in smooth muscle	None, smooth‐muscle‐restricted Ogt deletion used as genetic tool	Deletion attenuated phosphate‐induced mineralization, reversed by pharmacological Wnt activation [19], and reduced lesion burden while restoring contractile markers in hyperglycemic Apoe‐deficient mice [120]. No cell‐selective OGT‐directed agent exists; see the caution in Block H.	VSMCs; smooth‐muscle Ogt‐knockout mice	Genetic validation only, no agent reported	E3	[19, 120]
G. Palmitoylation						
ZDHHC1	None, Zdhhc1 deficiency used as genetic tool	Loss of ZDHHC1 drives nuclear translocation of PI3K p110α, suppressing PI3K–AKT–mTOR signaling and reducing oxLDL uptake, foam‐cell formation, and lesion burden. Allosteric ZDHHC1 inhibition is a proposal; no such inhibitor has been reported.	ApoE^−/−^ mice; macrophages	Genetic validation only—No agent reported	E3	[121]
H. Cautions — interventions in these pathways that worsened disease or gave discordant human data						
O‐GlcNAcase (OGA)	Thiamet‐G (OGA inhibitor)	Elevating O‐GlcNAc levels accelerated vascular calcification and worsened aortic compliance in diabetic mice. Mechanistically, AKT was O‐GlcNAcylated at Thr430 and Thr479, promoting Ser473 phosphorylation and Runx2 transactivity. Substitution of both residues blocked calcification. Pharmacological OGA inhibition is therefore expected to be harmful to the vessel wall.	Diabetic mice; primary VSMCs	Caution	E3‡	[138]
NAD^+^ metabolism	NAD^+^ precursor supplementation	NAD^+^ boosting increased plaque area and inflammation, with CD38 upregulation and NAD^+^ toward PARP1 rather than sirtuins. Untargeted NAD^+^ augmentation cannot be assumed to be atheroprotective	ApoE^−/−^ mice	Caution	E3	[48]
Systemic OGT activity	None, human observational data	In the China PEACE‐MPP cohort, low serum OGT activity was associated with more major adverse cardiovascular events, and higher activity was associated with a lower adjusted risk, which is the opposite of the murine smooth muscle data. Serum OGT activity and VSMC O‐GlcNAc cycling are not the same variable, and neither data set predicts the effect of a systemic OGT‐directed agent.	Prospective human cohort	Caution	E2	[207]
PI3K/AKT/mTOR (erythropoietin)	Erythropoietin	EPO‐driven PI3K/AKT/mTOR activation increases intraplaque angiogenesis, a feature associated with plaque destabilization. Relevant to risk–benefit assessment when erythropoiesis‐stimulating agents are used in patients with vascular disease.	Endothelial cells; atherosclerosis models	Caution	E3	[34]

*Note:* Evidence grades (Section 5). E1, human causal evidence; E2, human observational evidence; E3, in vivo perturbation of a defined enzyme in an animal model with a vascular outcome; E4, in vitro mechanistic evidence with a defined enzyme and substrate; E5, associative or computational evidence without functional validation. ‡, acceptor residue mapped and tested by substitution; may be appended to any grade. †, source has not completed peer review. Six of the 24 entries have no agent at all. Listerin–ABCA1, SENP3–NLRP3, CBX4, smooth‐muscle OGT, ZDHHC1, and systemic OGT activity rest on genetic manipulation or on human observation alone; the Listerin entry additionally uses a tool compound rather than a therapeutic candidate. Presenting these as available strategies would misrepresent the field: They are target nominations, and the molecules needed to test them do not yet exist. No entry is isoform‐selective for the enzyme named. TMP195 inhibits class IIa HDACs as a group; the natural products acting on SIRT3, SIRT7, MAPK1, and NF‐κB/MAPK have not been shown to be selective; PYR‐41 inhibits ubiquitin activation globally; and 3‐aminobenzamide and trifluridine are not PARP1‐selective. Selectivity, rather than target identification, is the principal unsolved problem in this field. PP2A is deliberately omitted. The opposing results in Table 3, in which PPP2CB activity aggravates LOX‐1/MAPK signaling while PP2A activation suppresses the same axis, mean that neither activation nor inhibition can be recommended without holoenzyme‐level resolution. No ISGylation entry is included, because no agent directed at ISG15 conjugation or deconjugation has been tested in a vascular model.

Five grades are used. **E1** denotes human causal evidence: an interventional trial of an agent directed at a PTM enzyme, or a human genetic association accompanied by functional characterization of the implicated variant. In this review, the standard is met only by the HDAC9 axis, where risk alleles associated with carotid and coronary atherosclerosis [144, 145] are supported by mechanistic dissection of the associated cis‐regulatory element in human tissue [115]. **E2** denotes human observational evidence, in which a modification or its enzyme is measured directly in human tissue or human primary cells, as in the comparison of histone acetylation and methylation across carotid plaques of differing severity [160]. **E3** denotes in vivo evidence in which a defined enzyme is genetically or pharmacologically perturbed in an animal model and a vascular outcome is measured, most often lesion burden but in some studies vascular calcification, aortic structure, or vascular ageing, as in smooth‐muscle‐restricted *Ogt* deletion [19, 120] or NAD^+^ supplementation in *Apoe*‐deficient mice [48]. Where the measured endpoint is not lesion burden, this is stated in the relevant table row. **E4** denotes in vitro mechanistic evidence with a defined enzyme and substrate. The suffix ‡ is applied to any grade, most often E3 and E4, when the acceptor residue has additionally been mapped and tested by substitution, since residue‐level evidence is what separates demonstrated from inferred cross‐talk (Section 7.7). **E5** denotes associative or computational evidence: differential expression, correlation with disease status, network pharmacology, or in‐silico prediction without functional validation (Figure 2).

**Figure 2:**
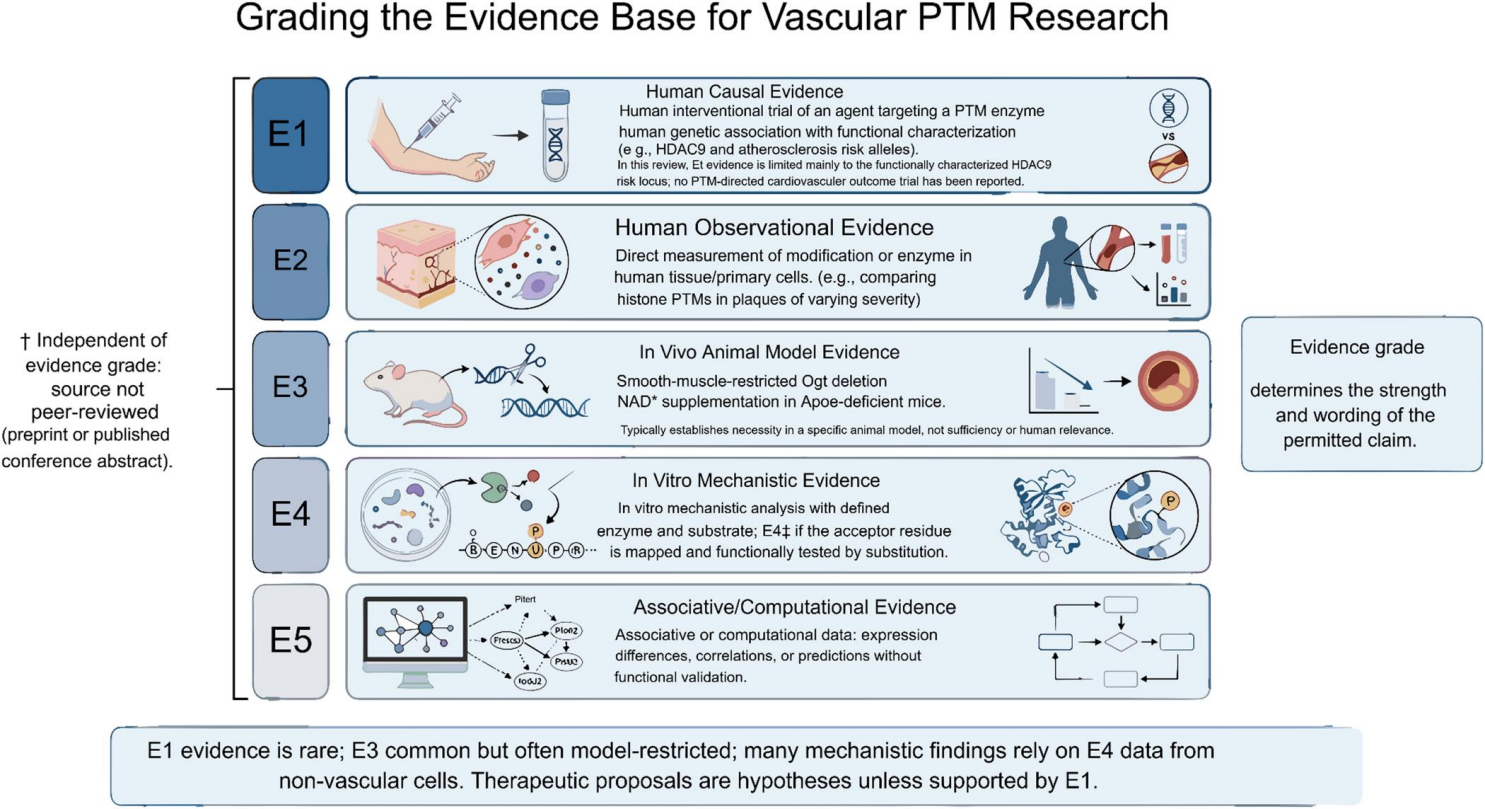
Evidence‐grading framework for vascular post‐translational modification research. Evidence is classified from E1, human causal evidence, to E5, associative or computational evidence, with the suffix ‡ appended to any grade to indicate that the acceptor residue was mapped and tested by substitution. The † symbol denotes non‐peer‐reviewed sources independently of grade. Evidence grade determines the strength and wording of claims, highlighting the scarcity of E1 evidence and the predominance of model‐restricted E3 and nonvascular E4 studies.

Two considerations apply independently of the grade. First, we mark with **†** any source that has not completed peer review, namely preprints and published conference abstracts. Such sources may prove entirely reliable, but their claims have not been externally assessed, and readers are entitled to know which statements depend on them. Second, the grade constrains the verb. A computational study nominates candidates but does not establish whether a compound prevents disease. An abstract reporting mass spectrometric detection supports the statement that a modification was detected; however, it does not support a quantitative comparison between tissue compartments. We have revised the manuscript so that its verbs correspond to the evidence.

Applying this scheme across the field yields three observations that shape the remainder of this review. The E1 category is nearly empty: no PTM‐directed agent has been evaluated in a cardiovascular outcome trial, and the human genetic evidence converges almost entirely on a single locus. The E3 category is well populated but weighted heavily toward loss‐of‐function models in hyperlipidemic mice, which establish necessity within one disease model rather than sufficiency or human relevance. A substantial share of the mechanistic relationships most often invoked in this literature, including several of the cross‐talk mechanisms examined in Section 7, rest on E4 evidence obtained in non‐vascular cells, a limitation stated explicitly in Section 7.7. Readers should accordingly treat the therapeutic proposals developed in Section 8 as hypotheses generated by E3 and E4 evidence rather than as conclusions supported by E1 evidence.

## 6 Role of Posttranslational Modifications in Atherosclerosis

### 6.1 Phosphorylation in Atherosclerosis

#### 6.1.1 Phosphorylation‐Driven Inflammatory Signaling Networks in Atherosclerosis

Phosphorylation is the most immediate molecular switch in the vasculature, converting mechanical, lipid‐derived, and immune inputs into discrete changes in protein function and transcriptional output. Its significance in atherosclerosis lies less in any single substrate than in the principle that the outcome is determined by cellular context, upstream stimulus, and site specificity. This is most clearly illustrated by endothelial cells. In disturbed‐flow regions, PKN1‐mediated phosphorylation of histone H3.3 at serine 31 links mechanotransduction to pro‐inflammatory chromatin remodeling, showing how hemodynamic cues are translated into durable transcriptional programs [22]. However, within the same compartment, IL‐33‐induced ERK1/2 phosphorylation restrains IRF1‐dependent VCAM‐1 expression, reducing leukocyte adhesion and lesion formation [131]. Therefore, MAPK activation is not intrinsically pathogenic; its consequences depend on the upstream trigger and the downstream transcription factor engaged.

Receptor‐initiated signaling reinforces the proatherogenic arm. MS4A6A activation promotes oxLDL‐induced endothelial dysfunction through IKK/NF‐κB, enhancing monocyte recruitment [130], whereas flow‐activated c‐REL sustains an inflammatory endothelial phenotype via p38 MAPK and non‐canonical NF‐κB cross‐talk, with pathway blockade lowering plaque burden [33]. Thus, a p38‐ and NF‐κB‐linked phosphorylation landscape operates in parallel with and in opposition to protective ERK‐driven programs.

Macrophages exhibit the same divergence at the interface of inflammation and lipid handling. WEE1‐mediated phosphorylation of NF‐κB p65 at Ser536 directly drives inflammatory transcription and lesion progression [32], and interventions that dampen this arm interrupt the feed‐forward loop, as fucosterol does by suppressing NF‐κB and p38/ERK phosphorylation [36] (E3). Not every beneficial intervention acts by suppression, however: calycosin‐7‐glucoside limits oxLDL‐induced foam‐cell formation by *activating* ATF‐1 downstream of p38 MAPK [136] E4), which redirects the cascade rather than inhibiting it and illustrates that p38 output is not uniformly pathogenic. Phosphorylation extends beyond canonical inflammatory cascades, with MAPK1 implicated in ferroptosis and targeted by resveratrol to constrain lipid peroxidation and inflammatory injury [151].

Reparative signaling completes the picture. miR‐126‐enhanced ERK phosphorylation in mesenchymal stem cells promotes endothelial repair after vascular injury, underscoring a reparative role for selective MAPK activation [49], and PKA‐dependent TRPV6 signaling with UCP2 activation limits inflammatory damage and apoptosis in the coronary endothelium [155].

Collectively, these findings define phosphorylation as a bidirectional regulator: identical modifications can sustain pro‐atherogenic feed‐forward loops or preserve vascular homeostasis. Therapeutically, selective modulation of these events, guided by high‐resolution phosphoproteomics, may suppress pathological inflammation without impairing protective signaling, a duality contrasted in Figure 3.

**Figure 3:**
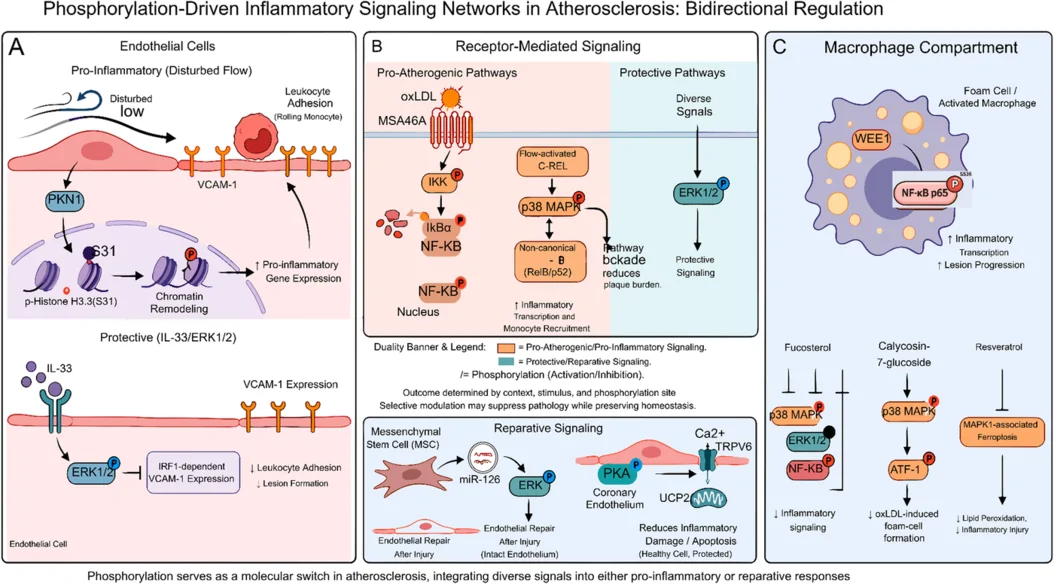
Phosphorylation‐driven inflammatory signaling networks in atherosclerosis. (A) In endothelial cells, disturbed flow promotes PKN1‐dependent phosphorylation of histone H3.3 at Ser31, driving chromatin remodeling and pro‐inflammatory gene expression, whereas IL‐33‐induced ERK1/2 signaling suppresses IRF1‐dependent VCAM‐1 expression and reduces leukocyte adhesion and lesion formation.(B) Receptor‐mediated oxLDL/MS4A6A–IKK–NF‐κB and flow‐activated c‐REL–p38 MAPK/non‐canonical NF‐κB pathways promote inflammatory transcription and monocyte recruitment, while ERK1/2‐ and PKA‐dependent reparative signaling supports endothelial protection and repair. (C) In macrophages, WEE1‐mediated phosphorylation of NF‐κB p65 at Ser536 enhances inflammatory transcription and lesion progression, whereas fucosterol, calycosin‐7‐glucoside, and resveratrol attenuate inflammatory signaling, foam‐cell formation, ferroptosis, lipid peroxidation, and inflammatory injury.

#### 6.1.2 Phosphorylation‐Mediated Regulation of PI3K/AKT/mTOR and MAPK Pathways in Atherosclerosis

Phosphorylation within the PI3K/AKT/mTOR axis governs macrophage autophagy and foam cell fate, positioning nutrient sensing as a determinant of plaque progression. Leucine‐driven mTORC1 phosphorylation promotes atherosclerosis by suppressing autophagy, illustrating how nutrient‐signaling cross‐talk operates during lesion development [147]. Reciprocal manipulation is protective: lactoferrin attenuates atherosclerosis through AMPK‐dependent suppression of mTOR signaling with restoration of macrophage autophagic flux [148]. and the notoginsenoside R1–saikosaponin B2 combination reduces PI3K/AKT/mTOR phosphorylation with the same effect on autophagy [149]. The two agents were tested separately and should not be described as a combination. Upstream regulators converge on the same node by different routes: miR‐125b‐1‐3p acts through RRAGD to modulate mTOR/ULK1‐dependent autophagy [150] and Zdhhc1 deficiency reduces p110α palmitoylation, promoting its nuclear translocation and suppressing AKT–mTOR signaling [121]; both limit foam‐cell formation.

The axis extends beyond the myeloid cells. S1PR2 activation elevates AKT/mTOR and ROCK1 phosphorylation, driving smooth muscle hypertrophy and endothelial barrier dysfunction [35], and erythropoietin promotes intraplaque angiogenesis through AKT/mTOR phosphorylation [34]. The strength of evidence varies across this network: network pharmacology and molecular docking nominate ERK signaling as a candidate calycosin target (E5, in‐silico), a prediction still awaiting experimental validation [153], whereas icariin confers protection through miR‐205‐5p/ERBB4‐dependent regulation of AKT phosphorylation [152].

Together, these findings establish the PI3K/AKT/mTOR and MAPK nodes as master phosphorylation‐dependent regulators of atherogenesis, and their defining feature is duality: the same cascades drive inflammation, proliferation, and plaque progression under some conditions, yet preserve autophagy and vascular protection under others. This ambivalence is a therapeutic constraint rather than a detail; global kinase inhibition is unlikely to succeed where pathway‐selective, cell‐type‐specific modulation is required. Translational priority therefore belongs to phosphoproteomic profiling of human atherosclerotic plaques, which could stratify patients by pathway activity and define intervention windows that suppress maladaptive signaling while preserving protective responses.

#### 6.1.3 Phosphorylation‐Dependent Modulation of Endothelial Integrity and Vascular Remodeling

Phosphorylation operates as a rapid, reversible toggle that couples extracellular stress signals to intracellular effectors, and in the endothelium, this coupling determines whether the vessel wall moves toward repair or disease. Lining the blood–tissue interface, ECs are continuously exposed to hemodynamic shear, metabolic disturbance, and inflammatory cytokines, and the phosphorylation of structural proteins, ion channels, and signaling intermediates translates these inputs into divergent vascular outcomes. One persistent protective axis links metabolic sensing to endothelial electrophysiology. AMPK activation enhances KCa2.3 channel phosphorylation, restoring endothelium‐dependent hyperpolarization and nitric oxide availability and reversing angiotensin II‐induced dysfunction [132]; in diet‐induced obese mice, AMPK improves endothelial function by increasing KCa2.3 channel abundance rather than by phosphorylating the channel [133]. AMPK thus acts as an energy sensor with therapeutic promise for early‐stage atheroprotection.

Phosphorylation of junctional and cytoskeletal targets exerts an opposite effect. Vinculin phosphorylation disrupts actin–junction anchoring, widening endothelial gaps and permitting leukocyte permeation [129]. A comparable barrier‐disrupting outcome follows S1PR2‐mediated phosphorylation of AKT, mTOR, and ROCK1, which drives smooth muscle growth alongside increased endothelial permeability [35]. Erythropoietin engages the same AKT/mTOR phosphorylation route to induce intraplaque neovascularization [34], indicating that proliferative phosphorylation programs are contextually useful in ischemic repair yet destabilizing when neovessels form within plaques.

MAPK‐associated phosphorylation is similarly bidirectional. Icariin inhibited ERBB4/AKT phosphorylation through miR‐205‐5p regulation to block endothelial activation [152]; silymarin activated ERK5 phosphorylation to strengthen antioxidant defense against oxLDL [54], and miR‐497‐5p attenuated VEGFA‐induced p38 phosphorylation, reducing inflammatory angiogenesis [37]. Fine‐grained pathway targeting, activating ERK5 while inhibiting p38 or AKT/mTOR, may therefore isolate protective phosphorylation signals. Transcription factor phosphorylation provides a further fulcrum: myeloid LXRα Ser196Ala substitution induced FoxM1 and increased lesion‐resident cell proliferation and plaque burden, while also producing smaller necrotic cores and enhanced efferocytosis [135], (E3‡). The phenotype is mixed rather than uniformly pro‐atherogenic, and it illustrates that a single nuclear phospho‐switch can move lesion size and lesion composition in opposite directions.

Collectively, phosphorylation emerges not as an indiscriminately pathogenic or beneficial process but as a context‐dependent molecular switch whose direction is set by the target and cellular setting. Mechanistically, this suggests the hypothesis that therapies preserving AMPK, ERK5, and PKA signaling while preferentially blunting AKT/mTOR, ROCK1, and p38 signaling would be favorable; the proposition rests on E3 and E4 evidence in rodent and cell models and has not been tested with a selective agent in any system.

#### 6.1.4 Phosphatase‐Mediated Control of Lipid Metabolism and Endothelial Inflammation in Atherosclerosis

In the endothelium, oxLDL engagement of LOX‐1 triggers a cascade through ERK/p38 signaling and ROS production, which together promote leukocyte adhesion, barrier disruption, and inflammation [156]. Against this background, the serine/threonine phosphatase PP2A yields apparently opposite results in the same axis. Increased activity of the catalytic subunit PPP2CB amplifies LOX‐1‐mediated MAPK/ERK activation, worsening dyslipidaemia and accelerating plaque development [156] (E3), yet PP2A activation suppresses the same LOX‐1/ROS/MAPK axis, restoring endothelial function and lowering inflammatory tone [157] (E4). This contradiction resolves once PP2A is treated not as a single enzyme, but as a multiform holoenzyme family whose regulatory B‐subunit composition dictates substrate specificity, subcellular compartmentalization, and ultimately biological outcome.

In the myeloid compartment, by contrast, the evidence points consistently in one direction. PP2A deficiency drives macrophage conversion to a lipid storage phenotype, increasing foam cell formation and lesion growth; here, PP2A appears to sustain ABCA1/ABCG1‐mediated cholesterol efflux while restraining NF‐κB/MAPK signaling, preserving a metabolic and inflammatory equilibrium that limits lesion formation [158]. Read together with the endothelial data, this establishes the governing principle: whether phosphatase activity is pathogenic or protective depends on context, specifically cell type and holoenzyme composition.

Tyrosine phosphatases add a parallel and mechanistically distinct layer of control. Myeloid‐specific PTP1B deletion improves cholesterol homeostasis through AMPK‐dependent mechanisms, favoring metabolic stress responses over inflammatory activation [159]. Whereas PP2A negatively regulates serine/threonine cascades, including MAPK and mTOR [157, 158], PTP1B reweights tyrosine kinase signaling towards macrophage lipid clearance and reduced inflammatory drive [159]. Selective phosphatase modulation is therefore an attractive therapeutic avenue, provided interventions engage the correct holoenzyme composition in the correct cell type and are tracked by pathway readouts that move in the intended direction. Table 3 consolidates the phosphorylation‐centered mechanisms spanning inflammatory signaling, PI3K/AKT/mTOR–MAPK cascades, endothelial remodeling, and phosphatase‐mediated regulation, integrating the dual pathogenic and protective roles identified in Sections 6.1.1–6.1.4.

### 6.2 Acetylation in Atherosclerosis

#### 6.2.1 Epigenetic Regulation of Atherosclerosis: HDAC9 Variants, Histone Modifications, and Plaque Progression

Epigenetic reprogramming, and histone deacetylation in particular, has been implicated in several mechanisms underlying atherosclerotic disease. Candidate‐gene association studies have linked the HDAC9 variant rs2107595 to advanced carotid atherosclerosis [144], and to coronary artery disease susceptibility and lesion severity [145] (E2). Enhanced activity of HDAC9 fosters a pro‐inflammatory, synthetic VSMC phenotype, enhances macrophage‐driven cytokine release, and accelerates plaque progression [21]. Histone modification patterns of decreased H3K9 acetylation and changed H3K27 trimethylation at areas controlling endothelial function play a role in leukocyte adhesion and endothelial activation [160]. Comparative analysis further reveals that the disease‐causing action of HDAC9 deacetylation is mechanistically different but functionally redundant with that of other histone modifiers, such as EZH2, which suppresses anti‐inflammatory transcription factors by deposition of H3K27me3 [160]. In preclinical models, class‐nonselective pharmacological HDAC inhibition reduced NADPH oxidase expression, oxidative stress, and lesion progression in hypercholesterolemic *Apoe*‐deficient mice [161], and enhanced PPARγ activity with reduced foam‐cell formation and lesion burden in the same background [162] (both E3). Neither study measured endothelial quiescence, and neither used an isoform‐selective agent.

#### 6.2.2 Histone Acetylation Dynamics in Atherosclerotic Vascular Inflammation

Histone acetylation contributes to the regulation of atherosclerosis by modulating vascular inflammation, EndMT, and plaque stability. It is an adaptive response that requires fine‐tuning between HATs, such as CREB‐binding protein (CBP), and HDACs, particularly HDAC9, that control EC and macrophage gene expression to modulate disease progression [21, 52, 115, 116]. CBP, a histone acetyltransferase, acetylates NF‐κB p65 and augments its transcriptional function in stimulated endothelial cells [52] (E4); no in vivo lesion data are reported for CBP, so its contribution to early atherogenesis is inferred rather than demonstrated. CBP increases adhesion molecules (VCAM‐1, ICAM‐1, and E‐selectin) in thrombin‐stimulated ECs, facilitating leukocyte‐endothelial adhesion. CBP knockdown reduces NF‐κB acetylation and adhesion molecule expression and lowers leukocyte–endothelial adhesion in vitro [52], indicating that CBP is required for thrombin‐induced endothelial activation. Conversely, HDAC9 promotes plaque vulnerability by deacetylation‐triggered activation of proinflammatory pathways. By deacetylating IKKα/β inhibitory κB kinases, HDAC9 increases macrophage and EC inflammation, enhancing lesional macrophage material and reducing fibrous cap thickness in hyperlipidemic mice [21]. Pharmacologic inhibition with the class IIa HDAC inhibitor TMP195 inhibits cytokine production (e.g., IL‐1β, IL‐6) and leukocyte recruitment stimulated by IKKβ, slowing lesion growth and stabilizing complex plaques [21]. HDAC9 also triggers EndMT, transdifferentiating EC into a mesenchymal phenotype, which destabilizes the endothelium. Endothelial *Hdac9* deletion in atherosclerosis‐prone mice preserves endothelial marker expression, reduces plaque area and lipid content, and increases fibrous cap thickness, indicating that endothelial HDAC9 activity promotes plaque instability [116]. An evolutionarily conserved cis‐regulatory element (CRE) also controls HDAC9 expression, connecting genetic risk variants with atherogenic inflammation. HDAC9 deacetylates NLRP3 within the NACHT and LRR domains to permit inflammasome activation and pyroptosis, and HDAC9 expression in human carotid endarterectomy specimens is associated with plaque instability [115]. Myeloid‐restricted deletion of the cis‐regulatory element up‐regulates *Hdac9* and exacerbates inflammation, whereas TMP195‐loaded nanoparticles directed to inflamed lesions stabilize plaques and reduce features associated with rupture [115].

CBP and HDAC9, mechanistically different, acetylation and deacetylation, have convergent activities, such as adhesion molecule expression and cytokine secretion [21, 52, 115, 116]. CBP functions mainly in the initial EC activation [52], whereas HDAC9 maintains chronic inflammation and EndMT in macrophages and ECs and influences early as well as late disease phases [21, 115]. Class IIa HDAC inhibition is atheroprotective across several models [21, 115] although TMP195 acts on class IIa HDACs as a group and the effect cannot be attributed to HDAC9 alone. CBP, by contrast, has a broad transcriptional role that makes selective pharmacological targeting correspondingly harder.

#### 6.2.3 Sirtuin‐Orchestrated Regulation of Atherosclerosis: Mechanistic Convergence and Divergence Across Isoforms

Cumulative evidence positions sirtuin family members (SIRT1–SIRT7) at the center of atherosclerosis through their capacity to integrate nutrient sensing, mitochondrial homeostasis, autophagy, and inflammatory signaling. Although these NAD^+^‐dependent deacylases share structural homology, individual isoforms exert distinct, and occasionally opposing, influences on vascular cells, shaping plaque development, composition, and stability. The studies summarized in Table 4 delineate a complex but coherent mechanistic landscape in which sirtuins function as both guardians and modulators of the atherogenic process. Elevated Nε‐carboxyethyl‐lysine (CEL), a major advanced glycation end product, dysregulates RAGE/LKB1/AMPK/SIRT1 signaling, impairing ZKSCAN3 acetylation‐dependent macrophage autophagy and promoting plaque instability in diabetic atherosclerosis [50] (E3). Pharmacological SIRT1 activation by SRT1720 restores PGC‐1α deacetylation, mitochondrial oxidative phosphorylation, and reverses VSMC senescence [45]. At the same time, SIRT1 loss of function shifts macrophage polarization toward a pro‐inflammatory phenotype through the TIMP3/ADAM17 axis [46] and promotes atherosclerosis through impaired autophagy [163]. SIRT1 levels differ across ischaemic coronary syndromes in patients with chronic and acute coronary artery disease [165] (E2) nd SIRT1 expression is inversely correlated with miR‐138‐5p in blood mononuclear cells from patients with atherosclerosis [164] (E5). Both findings are correlative; miRNA‐mediated repression of SIRT1 in vascular cells has not been demonstrated.

SIRT2, which is less characterized, also suppresses M1 macrophage polarization and therefore decreases plaque load and improves stability in LDLr^−^/^−^ mice [51]. SIRT3 acts as a mitochondrial sentinel that **attenuates** diabetic atherosclerosis, reportedly by suppressing endothelial LDL transcytosis through PINK1/Parkin‐dependent mitophagy (E3†, preprint) [166], by enhancing PPAR‐α/eNOS signaling [167], and by limiting vascular remodeling through mitochondrial acetyl‐CoA regulation and KLF4 suppression [168]. Pharmacological and nutraceutical stimulators, such as *Valeriana officinalis* extract [169] and melatonin [170], also have SIRT3‐mediated antioxidant, anti‐apoptotic, and antipyroptotic pathways, which further stabilize plaques.

SIRT4 is protective in two compartments by distinct mechanisms. In macrophages, SIRT4 deficiency produces lipid‐rich, inflammatory plaques through NF‐κB/IκB‐mediated CXCL2/3 upregulation [171]. In endothelial cells, SIRT4 is a mechanosensitive target of the PIEZO1 channel, induced by atheroprotective shear stress, and suppresses oxLDL uptake by downregulating LOX‐1 [172]. SIRT4 overexpression also inhibits oxLDL‐induced endothelial apoptosis and disrupts PI3K/Akt/NF‐κB signaling to attenuate pro‐inflammatory cytokine secretion [173].

SIRT5 is not discussed here because sufficient vascular mechanistic evidence was not identified. As a predominantly nuclear, chromatin‐associated deacylase, SIRT6 supports genomic stability and restrains vascular inflammation. SIRT6 deficiency in the endothelium induces lesion acceleration, enhances ICAM‐1 expression, and increases monocyte adhesion [174]. IL‐38‐induced increase in SIRT6 promotes HO‐1‐dependent autophagy and suppresses palmitate‐induced endothelial inflammation [175], hinting at an anti‐inflammatory cytokine–sirtuin pathway with therapeutic implications. SIRT7 protects against vascular aging and supports resistance to oxidative stress. Ganoderma lucidum spore powder (GLSP) increases SIRT7 activity, promoting Keap1 deacetylation, Keap1–Nrf2 dissociation, and Nrf2‐dependent antioxidant gene expression, and mitigating VSMC senescence and calcification in a vascular ageing model [176] (E3). The measured endpoint is vascular ageing rather than atherosclerotic lesion burden. Oxidative stress‐induced miR‐335‐5p, on the other hand, inhibits SIRT7, inducing endothelial dysfunction; inhibition of the miRNA or overexpression of SIRT7 restores endothelial function [177].

Together, these studies indicate three, partly overlapping, mechanistic platforms through which sirtuins restrain atherosclerosis: maintenance of mitochondrial homeostasis, principally by SIRT1 and SIRT3; restraint of inflammatory signaling and macrophage polarization by SIRT1, SIRT2, SIRT4, and SIRT6; and preservation of endothelial mechanosensitivity, redox balance, and genomic stability by SIRT4, SIRT6, and SIRT7. Several isoforms contribute to more than one platform, and the groupings are descriptive rather than mutually exclusive. Isoform‐specific distinctions, for example, SIRT3 predominance in diabetic atherosclerosis and SIRT7 in vascular aging, underline the necessity of precision targeting. These mechanisms are summarized in Figure 4, which shows how SIRT1–SIRT4, SIRT6, and SIRT7 converge on foam‐cell biology, NF‐κB signaling, mitochondrial homeostasis, and macrophage inflammatory responses. Table 4 provides a comparative synthesis of the HDAC9, CBP, and sirtuin axes across Sections 6.2.1–6.2.3.

**Figure 4:**
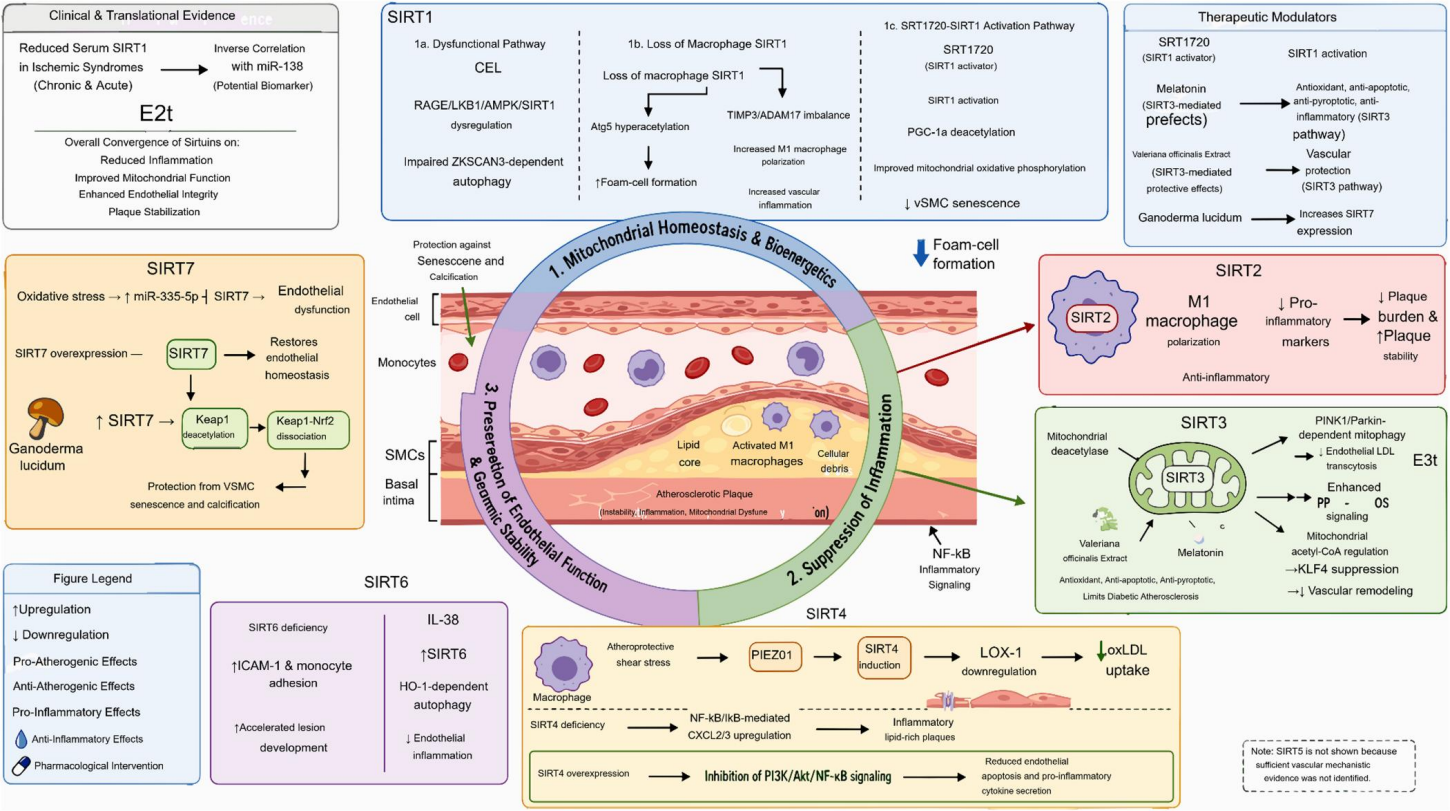
Sirtuin‐orchestrated regulation of atherosclerosis across vascular and immune compartments. SIRT1–SIRT4, SIRT6, and SIRT7 converge on mitochondrial homeostasis, inflammatory signaling, endothelial integrity, macrophage polarization, foam‐cell formation, and plaque stability through isoform‐ and context‐specific mechanisms. Human SIRT1 observations are graded E2 and E5 and are correlative; the SIRT3–PINK1/Parkin mitophagy pathway is graded E3† because it rests on a preprint that has not completed peer review; SIRT5 is omitted because sufficient vascular mechanistic evidence was not identified.

### 6.3 Ubiquitination in Atherosclerosis: A PTM Driving Pathogenesis and Protection

#### 6.3.1 Pro‐Atherogenic Roles of E3 Ubiquitin Ligases

Given the rapidly expanding evidence base, we provide an integrated summary of ubiquitination enzymes, their targets, mechanistic pathways, and translational relevance across vascular and immune compartments in atherosclerosis (Table 5). E3 ubiquitin ligases regulate vascular inflammation, lipid metabolism, and regulated cell death in atherosclerosis, and several of those characterized to date act pro‐atherogenically through substrate‐specific ubiquitination; others, considered in Section 6.3.2, are protective. These enzymes reorganize signaling to increase endothelial activation, macrophage polarization, and plaque destabilization in several vascular and immune cell compartments. One entry in this literature runs in the opposite direction: Peli1 deficiency, rather than Peli1 activity, promotes a destabilizing phenotype with increased foam‐cell accumulation, an enlarged necrotic core, compromised fibrous cap integrity, and a skewed adaptive immune profile [181] (E3), so Peli1 is protective when intact. No Peli1 substrate was identified in that study, and the entry is listed with the protective ligases in Table 5. This contrasts with TRIM25, which ubiquitinates XRCC1 in macrophages, releasing PARP1 activity and driving M1 polarization and programmed cell death; TRIM25 loss reduces lesion burden and fibrosis [118] (E3).

Endothelial damage is also a target, as ITCH induces ferroptosis by ubiquitin‐reliant protein degradation of ferritin light chain (FTL), making ECs vulnerable to oxLDL‐induced lipid peroxidation [178]. Likewise, TRIM21 triggers endothelial pyroptosis by promoting SOCS3 degradation, unleashing JAK/STAT3‐dependent NLRP3 activation [179]. In both cases an E3 ligase degrades a protein that restrains a death program, ferritin light chain in the case of ferroptosis and SOCS3 in the case of pyroptosis, with endothelial dysfunction as the common outcome. RNF128 impairs macrophage lipid handling by stabilizing rather than degrading SR‐B1 through Lys63‐linked polyubiquitination; the stabilized receptor increases oxLDL internalization and promotes foam‐cell formation rather than supporting cholesterol efflux [180] (E3). A closely analogous mechanism operates at the endothelial–smooth muscle interface. Endothelial‐derived Dickkopf‐1 transcriptionally induces USP53 in smooth muscle cells, and USP53 removes K48‐linked ubiquitin from SR‐A, stabilizing the scavenger receptor and promoting smooth‐muscle‐derived foam cell formation [53]. As with RNF128, a deubiquitinating activity is pro‐atherogenic because the substrate it protects is a lipid‐uptake receptor. These convergent roles of E3 ligases in promoting ferroptosis, pyroptosis, necroptosis, and impaired cholesterol handling across endothelial cells and macrophages are summarized in Figure 5. In contrast, Listerin, which also catalyzes Lys63‐linked polyubiquitination, acts in a protective mechanism by stabilizing ABCA1 and facilitating cholesterol efflux; its deficiency promotes plaque progression [182]. This difference indicates that the functional effect of polyubiquitination is determined by substrate identity and trafficking fate rather than by linkage type.

**Figure 5:**
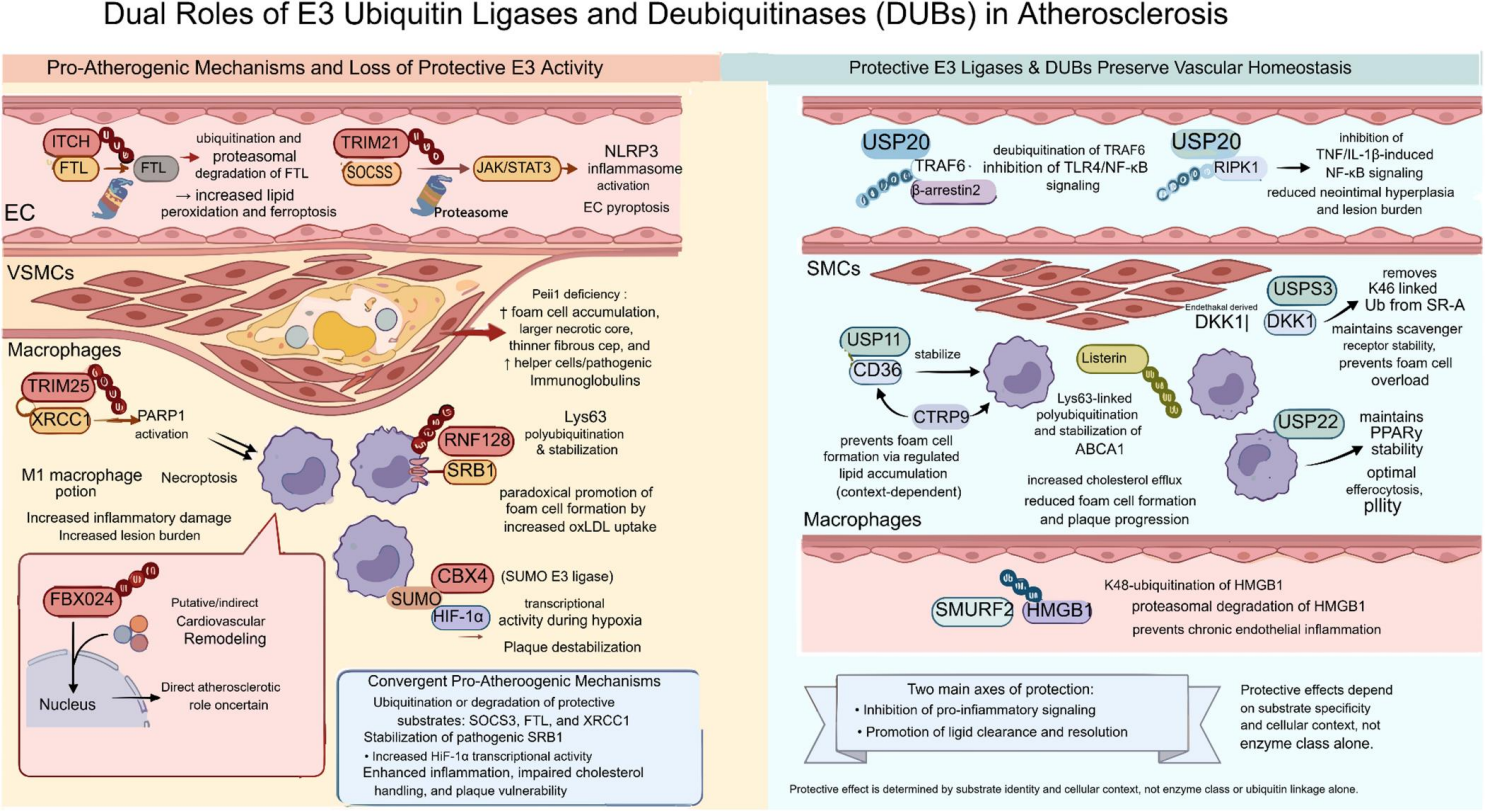
Dual roles of E3 ubiquitin ligases and deubiquitinases in atherosclerosis. Pro‐atherogenic mechanisms include ITCH‐, TRIM21‐, TRIM25‐, RNF128‐, and CBX4‐dependent regulation of endothelial cell death, macrophage inflammation, lipid uptake, and plaque instability, together with the destabilizing effects of Peli1 deficiency and the putative cardiovascular role of FBXO24. Protective pathways involve USP20‐mediated suppression of inflammatory signaling, USP11‐, Listerin‐, USP18‐, USP22‐, and USP53‐dependent regulation of lipid handling and resolution, and SMURF2‐mediated proteasomal degradation of HMGB1. These contrasting effects demonstrate that vascular outcomes are determined primarily by substrate specificity and cellular context rather than by enzyme class or ubiquitin‐linkage type alone.

Novel regulatory complexity is seen in FBXO24, which regulates FoxP1 stability through K48 and K63 ubiquitination and cross‐regulates transcription with its target. Its cardiovascular role is not established: the only available account of SCF^FBXO24^–FoxP1 reciprocal regulation is a preprint that reports no vascular phenotype [188] (E4†), and extrapolation to atherosclerotic remodeling is not supported by it. Finally, CBX4 belongs to a different conjugation system but is discussed here because the vascular literature routinely presents it as evidence of ubiquitin–SUMO cross‐talk. CBX4, a SUMO E3 ligase, promotes macrophage‐driven atherogenesis by SUMOylating HIF‐1α and increasing its transcriptional activity in the hypoxic plaque [43]. Whether SUMO and ubiquitin compete for the same HIF‐1α lysines has not been tested in vascular cells, and the relationship is therefore inferred from chemistry rather than demonstrated (Section 7.3).

#### 6.3.2 Protective Roles of E3 Ligases and Deubiquitinases (DUBs) in Atherosclerosis

E3 ubiquitin ligases and DUBs are key sentry functions for vascular homeostasis, suppressing pro‐atherogenic signaling through stabilizing anti‐inflammatory mediators, promoting cholesterol efflux, and maintaining endothelial integrity. USP20 is an anti‐inflammatory regulator in SMCs through dual mechanisms: in conjunction with β‐arrestin2, to reverse TRAF6 autoubiquitination to inhibit TLR4/NF‐κB signaling [117], and further by direct deubiquitination of RIPK1 to inhibit TNF‐ and IL‐1β‐induced NF‐κB activation [183]. Together, these activities reduce neointimal hyperplasia and lesion burden in vivo, (E3), indicating that restraining inflammatory signaling at the level of ubiquitin turnover is sufficient to alter lesion development in the models tested [183]. In macrophages, USP11‐mediated deubiquitination of CD36 acts together with C1q/TNF‐related protein 9 (CTRP9) and was reported to protect against atherosclerosis [184] (E4). Because CD36 is classically a driver of oxLDL uptake, the protective direction is counterintuitive and warrants independent confirmation.

Protective functions are also present in E3 ligases. SMURF2, an HECT‐domain ligase, protects ECs by inducing K48‐linked ubiquitination and proteasomal targeting of HMGB1, a powerful alarmin that otherwise enhances vascular inflammation [141]. By binding HMGB1 for proteasomal targeting, SMURF2 prevents chronic endothelial activation, a prime facilitator of early lesion development. Deubiquitinases also support cholesterol homeostasis. USP18 has been reported to stabilize ABCG1 in macrophages, increasing cholesterol efflux and limiting foam‐cell load [137], however, USP18 is enzymologically an ISG15‐specific isopeptidase with undetectable activity toward ubiquitin conjugates [186], so this mechanism is contested and is discussed in Section 7.6. USP22 stabilizes PPARγ, supporting efferocytosis and plaque stability [185] (E3).

A comparative examination of these mechanisms indicates two prevailing protection strategies: (i) inhibition of pro‐inflammatory cascades through degradation or inactivation of the upstream activators TRAF6, RIPK1, and HMGB1, and (ii) preservation of lipid‐clearance and resolution processes by stabilizing ABCG1 and PPARγ. Notably, the beneficial outcomes are dictated less by the enzymatic class (E3 ligase vs. DUB) than by substrate specificity and the cellular context in which the enzyme operates. From a translational perspective, these findings argue for substrate‐selective modulation as a therapeutic principle—enhancing protective E3/DUB–substrate interactions while sparing or even inhibiting pathogenic ones. This targeted accuracy would take advantage of the ubiquitin system's own ability to resolve inflammation and modulate vascular homeostasis with minimal disruption of ubiquitin dynamics elsewhere, reducing off‐target effects.

#### 6.3.3 Therapeutic Implications and Novel Approaches

Two preclinical strategies illustrate compartment‐selective targeting of the ubiquitin–proteasome system. Pharmacological inhibition of UBA1, the initiating E1 enzyme, with PYR‐41 reduced plaque development in *Apoe* ^−/−^ mice without altering systemic lipid metabolism or immune cell composition and lowered VCAM‐1, ICAM‐1, and MCP‐1 expression, NADPH oxidase isoforms, and macrophage IL‐1β and IL‐6 [146]. Separately, *circBTBD7‐420aa*, a protein encoded by hsa_circ_0000563, promotes ubiquitin‐proteasome‐mediated degradation of SLC3A2 and suppresses VSMC proliferation and migration; osteopontin‐targeted engineered exosomes were used to deliver it with cell‐type selectivity [187]. The two approaches address different compartments, macrophage inflammatory amplification and VSMC remodeling, respectively, and both depend on avoiding global disruption of proteostasis, a constraint discussed for all PTM classes in Section 8. Ubiquitin‐directed strategies are listed alongside those for the other modification classes in Table 8.

### 6.4 SUMOylation and Atherosclerosis

#### 6.4.1 Macrophage SUMOylation: Drivers and Resolvers of Foam Cell Formation and Inflammation

Macrophages are pivotal cellular mediators of atherogenesis, orchestrating lipid uptake, foam cell formation, and cytokine release. SUMOylation, a reversible post‐translational modification, has emerged as a critical regulatory layer modulating the inflammatory tone and lipid metabolism of macrophages in an isoform‐ and substrate‐specific manner. Recent mechanistic studies have converged on the notion that SUMOylation within macrophages can either accelerate or restrain atherosclerosis, depending on the E3 ligase, SUMO protease, and downstream substrate targeted. Proatherogenic activity is represented by CBX4, a SUMO E3 ligase, whose overexpression in macrophages increases lesion development in ApoE^−^/^−^ mice by SUMOylating HIF‐1α and enhancing its transcriptional activity, thereby amplifying the hypoxic pro‐inflammatory program of lesional macrophages [43]. Concurrently, TFEB SUMOylation, a master regulator of lysosomal biogenesis transcription, inhibits lysosomal function, lipid efflux suppression, and foam cell maturation [200]. These results indicate a feed‐forward loop in which SUMOylation is inhibitory and maintains macrophages in an inflammatory lipid‐storing state.

DeSUMOylation mechanisms can be, however, favorable. SENP3, a SUMO protease, was found to inhibit foam cell formation through the deSUMOylation of SUMO2/3 from NLRP3, which in turn restricts oxLDL‐induced inflammasome activation in macrophages [38]. Of interest, loss‐of‐function models validate that SENP3 deficiency increases NLRP3 SUMOylation, inflammasome activation, and lesion burden, placing SENP3 as an intrinsic regulator of athero‐inflammatory signaling [38]. Collectively, these studies define a bidirectional axis in which macrophage SUMOylation promotes pro‐inflammatory, foam cell–producing programs or maintains them in equipoise by substrate‐specific deconjugation.

#### 6.4.2 Endothelial and Vascular Wall SUMOylation in Stress Response and Dysfunction

ECs and VSMCs form the structural and functional core of the arterial wall, and their pathologic responses to hemodynamic, metabolic, and oxidative stress are the focus of atherogenesis. SUMOylation in endo‐ and VSMCs is an emerging pivotal posttranslational mechanism that coordinates phenotypic remodeling, inflammatory activation, and redox homeostasis with both beneficial and adverse functions. Hypoxia‐mediated SUMOylation of Fas‐associated death domain (FADD) protein in ECs initiates the RIPK1–RIPK3–MLKL necroptotic pathway, thus promoting cell death and vascular injury [134]. Similarly, SUMO2ylation of redox‐sensitive adaptor protein p66Shc initiates endothelial oxidative stress and reduction in nitric oxide (NO) bioavailability, ultimately leading to endothelial dysfunction [119]. In a second model, widespread overexpression of SUMO2 globally perturbed vascular tone and accelerated oxidative damage, placing SUMO2 on the radar as a putative pro‐atherogenic modifier in the endothelial compartment [208]. In the vascular media, SUMOylation of SRF induces phenotypic switching of VSMCs from contractile to synthetic phenotype, the underlying mechanism of vascular remodeling in atherogenesis [139]. SUMOylated SRF exhibits abnormal transcriptional activity towards smooth muscle contractile genes, induction of extracellular matrix deposition, and proliferative phenotype [139]. Remodeling, although adaptive during repair of injury, is maladaptive within the chronic inflammatory context of atherosclerosis.

In summary, SUMOylation within the blood vessel wall is a molecular switch that consolidates environmental inputs (e.g., hypoxia, non‐laminar flow, and oxidative stress) into transcriptional and signaling outputs that reconstruct cellular phenotype and function. However, the net impact on disease advancement depends on which SUMO paralog is involved, substrate specificity, and the initiating stressor.

#### 6.4.3 SUMOylation as a Metabolic–Inflammatory Switch in Atherosclerosis

SUMOylation tunes lipid handling and vascular inflammation in hepatic, vascular, and immune spaces and thus modulates atherogenesis. At the hepatic interface, LRH‐1 SUMOylation facilitates its corepressor interaction with PROX1, suppresses reverse cholesterol transport (RCT), and increases plaque formation; in contrast, ablation of LRH‐1 SUMOylation at K289 (K289R) increases RCT and lowers lesion burden in hypercholesterolemic mice, which demonstrates a causal, site‐specific link between a nuclear receptor SUMO mark and systemic cholesterol flux [20]. Interaction with nutrient sensing, AMPKα2, is a cardiometabolic advantage gatekeeper for fish oils: AMPKα2 activity represses GPR120 SUMOylation (by repressing UBC9 and evoking SUMO2/3), maintains β‐arrestin coupling/internalization, and is essential for ω−3 fatty acid anti‐inflammatory, anti‐atherosclerotic effects; ablation of AMPKα2 facilitates hyper‐SUMOylation of GPR120 and abolishes the benefit; the relationship is supported in human arteries by inverse AMPKα2–UBC9 correlations and by clinical subgroup analyses, both of which are correlativ [204].

In vascular smooth muscle, SUMO‐1‐dependent modification of SERCA2a safeguards Ca^2+^ homeostasis under diabetic stress: restoring SUMO1–SERCA2a conjugation reverses high glucose/palmitate–driven contractile‐to‐synthetic shift, dampens proliferation/migration, normalizes Ca^2+^ handling, and alleviates aortic injury and atherosclerosis in ApoE^−^/^−^ mice [201]. In the myeloid compartment, endocrine–immune crosstalk can conversely exploit SUMOylation to amplify inflammation: chemerin, via β_2_‐integrin signaling, triggers global SUMO2/3 conjugation in monocytes, SUMOylates HSP90AB1, and drives ERK1/2 activation and pro‐adhesive activation states, effects reversed by SUMO pathway inhibition (ML‐792) [205]. Human multi‐omics reinforce translational relevance: a CAD meta‐analysis across bulk and single‐cell data sets identifies differentially expressed SUMO‐related genes, nominating SUMO1 and PPARG as biomarker candidates enriched in endothelial populations and aligned with immune infiltration signatures; drug–gene network mapping further outlines actionable nodes in SUMO signaling [209].

Together, these studies indicate that SUMOylation acts in opposite directions on different substrates: pathogenic events (LRH‐1/PROX1, HSP90AB1) block cholesterol clearance or enhance leukocyte activation, whereas protective events (SERCA2a; AMPKα2‐restricted GPR120 de‐SUMOylation) maintain vascular tone and suppress inflammation. Therapeutically, precision is key: selectively inhibit LRH‐1 or HSP90AB1 SUMOylation while maintaining/enhancing SERCA2a and AMPKα2‐concordant signaling, and use SUMO‐axis biomarkers to stratify patients most likely to respond.

### 6.5 NEDD4L and Atherosclerosis

#### 6.5.1 Regulation of Macrophage Polarization and Lipid Metabolism by NEDD4L in Atherosclerosis

Macrophage polarization and lipid metabolism are key controllers of plaque phenotype and disease development in atherosclerosis, and recent research has revealed a bidirectional role for the ubiquitin E3 ligase NEDD4L in both processes. In an ApoE^−^/^−^ mouse model, miR‐30a‐5p was identified as a potent atheroprotective factor with a direct target on NEDD4L, thereby reducing the M1/M2 macrophage ratio and lowering oxidized LDL (oxLDL) uptake [203]. Mechanistically, NEDD4L knockdown reduced PPARγ ubiquitination, altered Smad phosphorylation, and increased expression of the cholesterol efflux transporters ABCA1 and ABCG1, supporting an anti‐inflammatory macrophage phenotype and improved lipid handling [203] (E3). These findings identify NEDD4L as the pro‐atherogenic effector that miR‐30a‐5p suppresses, so that reduced NEDD4L activity is the proximate cause of the protective phenotype. However, work in a diabetic atherosclerosis model defines a pro‐atherogenic role for NEDD4L delivered in exosomes released by endothelial cells exposed to high glucose and oxLDL [202]. Such NEDD4L‐rich exosomes were easily taken up by THP‐1 monocytes and induced strong M1 polarization, increased oxLDL uptake, and foam cell formation. The mechanism was NEDD4L‐dependent ubiquitination of IκBα and PPARγ, triggering the NF‐κB pathway and degrading lipid regulatory programs [202]. Most interestingly, pharmacological exosome secretion inhibition (GW4869) abolished the effects, emphasizing the significance of intercellular vesicular communication in maintaining inflammatory macrophage phenotypes within the diabetic environment [202]. Together, these results define a Janus‐faced role for NEDD4L in macrophage physiology: intracellular repression of NEDD4L by miR‐30a‐5p promotes atheroprotection through lipid efflux and M2 polarization, while exosomal NEDD4L from metabolically stressed endothelium promotes M1‐mediated inflammation and lipid accumulation.

#### 6.5.2 Regulation of EC Function, Angiogenesis, and Vascular Homeostasis by NEDD4 Family Members

EC integrity and angiogenic potential are critical for vascular homeostasis, and dysregulation is a central driver of atherogenesis. NEDD4 and NEDD4L act in opposite directions on regulators of EC biology, with dual protective and disease‐promoting functions based on upstream stimuli and interacting proteins. NEDD4 expression is downregulated by accelerated degradation in oxLDL‐stressed HUVECs, coinciding with increased oxidative stress, adhesion molecule expression, and endothelial dysfunction [210]. Gain‐of‐function experiments revealed that overexpression of NEDD4 reverses the aforementioned pathological changes through APEX1 DNA repair protein induction, restoration of nitric oxide synthase homeostasis, and inhibition of VCAM‐1/ICAM‐1 expression [210]. These observations point to a cytoprotective function of NEDD4 in blunting oxidative and inflammatory damage in the atheroprone endothelium [210]. NEDD4L, on the other hand, is a highly effective pro‐angiogenic factor under normal conditions. In HUVECs, NEDD4L overexpression increases tube formation, migration, and proliferation through coordinated activation of Akt, Erk1/2, and eNOS pathways and VEGFR2 and cyclins D1/D3 upregulation [211]. Pharmacologic inhibition of these kinases inhibits NEDD4L‐induced angiogenesis, establishing their mechanistic importance. This angiogenic program, while potentially reparative in ischemic settings, will most likely prove maladaptive in atherosclerosis by inducing intraplaque neovascularization and instability [211]. A third study identifies a further mechanism of diabetic endothelial injury, in which NEDD4L destabilizes RASGRP2 by ubiquitination in human cardiac microvascular endothelial cells exposed to combined high‐glucose and oxLDL stress. RASGRP2 overexpression, achieved through direct overexpression or adipose tissue‐derived mesenchymal stem cell exosome overexpression, enhances endothelial viability, migration, and angiogenesis, while also inhibiting permeability, apoptosis, and ROS production by activating Rap1/R‐Ras [212]. NEDD4L knockdown stabilizes RASGRP2 and recapitulates these protective effects, while silencing of RASGRP2 abrogates the benefit, establishing the NEDD4L–RASGRP2 axis as a key regulator of diabetic atherogenesis [212]. Together, these results demonstrate the pleiotropic function of NEDD4 family ligases on endothelial biology. NEDD4 is protective against oxLDL‐induced endothelial injury in the systems tested, whereas NEDD4L is pro‐angiogenic through AKT, ERK1/2, and eNOS signaling and also aggravates diabetic stress‐induced endothelial damage by degrading RASGRP2.

#### 6.5.3 Related E3 Ligases in Vascular and Systemic Lipid Handling

Two further ligase‐substrate pairs are involved in atherogenesis. Hepatic lipocalin‐2 overexpression reduced plaque burden in *Ldlr* ^−^/^−^ mice by inhibiting Nedd4‐1‐mediated ubiquitination of SR‐BI at Lys500/Lys508; the protection was lost in hepatocyte‐specific *Nedd4‐1* or *SR‐BI* knockouts and in SR‐BI K500A/K508A mutants, establishing residue‐level specificity [213]. This is a systemic rather than vascular‐cell‐autonomous mechanism and is one of the few examples in this review in which acceptor lysines were mapped and tested. In the endothelium, disturbed shear stress enhanced TRIM21‐mediated degradation of MAPK6, relieving MAPK6‐dependent restraint of EGR1/CXCL12 signaling; endothelial MAPK6 overexpression reduced lesion formation in *ApoE* ^−^/^−^ mice and endothelial deletion increased it, with reversal by CXCL12 neutralization [140].

### 6.6 O‐Glcnacylation and Atherosclerosis

#### 6.6.1 Clinical and In Vivo Evidence Linking O‐GlcNAcylation to Atherosclerosis Progression

Emerging evidence positions O‐GlcNAc transferase (OGT)–mediated protein O‐GlcNAcylation as a pivotal modifier of atherosclerotic disease trajectory in both high‐risk human populations and experimental models. The China PEACE‐MPP cohort (*n* = 1947) provides one of the largest population‐based data sets to interrogate this association [207]. Counterintuitively, participants with low serum OGT activity experienced a higher incidence of major adverse cardiovascular events (MACE), comprising cardiovascular death, nonfatal myocardial infarction, and ischaemic stroke, over a median 5.56 years of follow‐up than those in the high‐activity group (10.4% vs. 7.6%; *p* = 0.032). After multivariable adjustment, higher OGT activity was associated with a lower hazard of MACE (HR 0.738, 95% CI: 0.547–0.997; *p* = 0.048) [207] (E2). The confidence interval approaches unity, so the adjusted association is statistically fragile and should not be read as a precise 26% risk reduction.

In contrast, mechanistic in vivo data from genetically modified murine models highlight a pro‐atherogenic role for OGT in VSMCs [120]. In hyperglycemic ApoE‐deficient mice fed a Western diet, tamoxifen‐inducible deletion of smooth muscle OGT (smOGTKO) markedly reduced aortic plaque lipid burden, intimal lesion area, and infiltration by leukocytes and α‐smooth muscle actin–positive cells [120]. This was accompanied by increased expression of contractile markers and by reduced YY1 and SRF expression, two transcription factors implicated in VSMC de‐differentiation [120]. These data support a causal role for smooth‐muscle OGT in VSMC phenotypic switching under hyperglycaemic stress (E3), although the O‐GlcNAc acceptor residues responsible were not identified.

Together, these data indicate that smooth‐muscle OGT activity is pathogenic in experimental hyperglycaemia, whereas serum OGT activity in humans is associated with lower cardiovascular risk. The two measurements are not equivalent, and the human association may reflect broader metabolic and stress‐response processes rather than vascular O‐GlcNAc cycling. Candidate explanations include tissue‐specific substrate repertoires, O‐GlcNAc cycling divergent roles in the endothelium versus smooth muscle, and confounding by comorbid metabolic states in observational cohorts. Closing this gap will require cell‐type‐resolved multi‐omic profiling of O‐GlcNAcylation in human vascular tissue, together with interventional studies that manipulate OGT activity in a tissue‐restricted manner.

#### 6.6.2 Molecular Mechanisms of O‐GlcNAcylation in Vascular Pathology

O‐GlcNAcylation has pleiotropic influences on vascular cell fate by direct modification of osteogenic, inflammatory, and metabolic signaling pathways. Pharmacological elevation of O‐GlcNAc by O‐GlcNAcase inhibition with thiamet‐G accelerates vascular calcification and reduces aortic compliance in diabetic mice, with induction and transactivation of Runx2 and downstream osteogenic markers [138] (E3‡). The mechanism is residue‐resolved: AKT is O‐GlcNAcylated at Thr430 and Thr479, which promotes Ser473 phosphorylation and mTORC2 binding, and substitution of both residues blocks Runx2 transactivity and calcification [138].

Complementing this pathway, recent in vivo work in CKD and high‐phosphate models implicates OGT‐dependent β‐catenin O‐GlcNAcylation in promoting Wnt/β‐catenin signaling‐driven calcification [19]. Smooth muscle‐specific Ogt deletion markedly attenuates phosphate‐induced osteogenic conversion and vascular mineral deposition, effects that are reversible by pharmacologic Wnt activation. Here, O‐GlcNAcylation promotes β‐catenin stability and transcriptional activity, expanding the mechanistic scope beyond Runx2 to encompass canonical Wnt signaling as an essential convergence point of O‐GlcNAc‐evoked vascular pathology [19]. Beyond VSMCs, O‐GlcNAcylation's metabolic derangements converge with endothelial damage programs. Accumulation of N‐acetylneuraminic acid (Neu5Ac) suppresses glutaminolysis through GLS2 induction, leading to mitochondrial damage and pyroptotic cell death of endothelium [214]. The Neu5Ac‐dependent effects were not shown to require O‐GlcNAcylation, and the study is cited here only as evidence that hexosamine and sialic acid metabolism converge on the same endothelial injury programs; a direct interaction with O‐GlcNAc cycling was not tested [214]. Together, these mechanistic analyses delineate a multi‐node signal network in which O‐GlcNAcylation regulates critical transcriptional and signal modules AKT–Runx2, Wnt/β‐catenin, and metabolic–pyroptotic axes to regulate vasculature calcific and inflammatory remodeling.

### 6.7 Palmitoylation and Atherosclerosis

#### 6.7.1 Palmitoylation in Endothelial Function in Atherosclerosis

S‐palmitoylation, the reversible covalent attachment of palmitate to cysteine thiols, is emerging as a regulator of endothelial homeostasis and atherogenesis. This lipid modification has recently been placed at the interface of structural integrity, metabolic signaling, and inflammatory activation within the vascular wall. A mechanistic link between dietary saturated fat and endothelial damage was established by the identification of pyruvate kinase M2 (PKM2) as an in vivo palmitoylation substrate in vascular endothelial cells [215]. Circulating palmitic acid concentrations were higher in patients with coronary heart disease and were associated with major adverse cardiovascular events and mortality [215] (E2). Administration of palmitic acid to *ApoE*^−/−^ mice raised blood pressure, impaired myocardial contractile function, and promoted plaque development (E3). Mechanistically, ZDHHC13‐mediated S‐palmitoylation of PKM2 at Cys31 impaired tetramer formation, reduced glycolytic capacity, and disturbed endothelial energy homeostasis [215]. These effects were attenuated by the nonselective palmitoylation inhibitor 2‐bromopalmitate, whereas blocking depalmitoylation aggravated endothelial dysfunction. Endothelium‐restricted expression of the palmitoylation‐deficient PKM2 Cys31Ser mutant provided the residue‐level causal tes [215] E3‡). Conversely, the structural function of PA‐dependent palmitoylation in maintaining endothelial function has been highlighted by other research studies. Intracellular PA is sequestered by lipid droplets (LDs), and this abrogates palmitoylation of ciliary proteins such as ARL13B, causing loss of primary cilia in ECs [122]. Cilia are mechanosensing centers that transmit anti‐atherogenic signals in response to laminar shear stress. In male mice, lipid‐droplet‐mediated ciliary loss disrupted protective mechanosensitive signaling and increased vascular inflammation and plaque burden; whether the same applies to female animals was not determined [122]. Increasing available palmitate, either by inhibiting stearoyl‐CoA desaturase 1 or by dietary supplementation, restored ciliary protein palmitoylation and ciliary architecture and reduced atherosclerosis [122] (E3). Excess modification by PA of metabolic enzymes such as PKM2 leads to an energy imbalance and endothelial dysfunction, while an adequate supply of PA is required for palmitoylation of structural ciliary proteins to ensure vascular homeostasis.

#### 6.7.2 Palmitoylation in Immune and Multicellular Regulation of Atherogenesis

Current multiomics studies have expanded the conceptual boundaries of palmitoylation from endothelial dysfunction to uncover its significant roles in immune regulation and multicellular communication within atherosclerotic plaques. Integrated bulk and single‐cell transcriptomic analysis of coronary artery disease data sets identified 534 differentially expressed palmitoylation‐related genes (DE‐PRGs), among which CXCL12, KRTAP4‐7, and PPP2R2B were nominated as hub nodes [216] (E5). They were strongly correlated with immune infiltration signatures, such as macrophage, fibroblast, and EC signatures, as indicated by single‐cell sequencing. Functional enrichment analyses associated these candidates with chemokine signaling, cytoskeletal reorganization, and inflammatory regulation, suggesting that they may represent potential disease‐associated biomarkers and candidate diagnostic indicators; however, their mechanistic roles and diagnostic utility require experimental and clinical validation [216]. An HDAC2–miR‐23a‐3p regulatory relationship was predicted computationally, suggesting a possible epigenetic input to palmitoylation‐related processes in coronary artery disease; the prediction has not been validated experimentally [216] (E5). At the same time, mechanism‐based analysis of Zdhhc1, a palmitoyl acyltransferase–defined its pro‐atherogenic function in foam cell biology [121]. Macrophage‐specific deletion of Zdhhc1 effectively inhibited oxidized LDL uptake, inhibited foam cell formation, and minimized plaque burden in ApoE^‐/‐ mice. Mechanistically, loss of Zdhhc1 reduced palmitoylation of the PI3K catalytic subunit p110α, permitting its nuclear translocation and suppressing downstream AKT–mTOR signaling [121] (E3). The overall outcome was decreased macrophage invasion into lesions and decreased inflammatory progression [121]. Taken together, these findings underscore a dual paradigm of immune‐related palmitoylation in atherosclerosis: a systems‐level perspective in which broad palmitoylation gene networks integrate immune and stromal responses and a reductionist, enzyme‐specific lens showing how targeted suppression of pathogenic palmitoylation (e.g., Zdhhc1–p110α axis) can disrupt foam cell–driven lesion growth.

### 6.8 ISGylation and Atherosclerosis

#### 6.8.1 ISGylation as a Dual Modulator of Atherogenesis: Linking Macrophage Cholesterol Efflux and Endothelial Inflammation

ISG15 and ISGylation are multifunctional regulators of vascular homeostasis that act in several cell types implicated in atherosclerosis. Evidence now indicates that ISG15‐dependent processes are protective in macrophages and endothelial cells but maladaptive in vascular smooth muscle; therefore, the net effect is contingent on cell type and substrate (Section 6.8.2). Overexpression of ISG15 in macrophage‐derived foam cells enhances cholesterol efflux by a non‐canonical autophagy‐dependent pathway [123]. In THP‐1‐derived foam cells, ISG15 suppresses miR‐17‐5p and thereby de‐represses the autophagy initiator Beclin‐1; the resulting increase in Beclin‐1 enhances cholesteryl ester hydrolysis and ABCA1‐dependent cholesterol efflux, reducing cellular lipid content [123] (E4). Pharmacologic inhibition of autophagy with 3‐methyladenine eliminates this effect, characterizing autophagy as the mechanistic pathway [123]. Such observations place ISG15 as a safeguard lipid‐handling regulator in macrophages to promote plaque regression. In endothelial cells, ISGylation acts as an anti‐inflammatory brake on NF‐κB p65 [124]. ISGylation of p65, catalyzed by the SCF^FBXL19^ E3 ligase complex, stabilizes the interaction between p65 and the phosphatase WIP1 and suppresses pro‐inflammatory serine phosphorylation. With pro‐inflammatory stimulation such as TNF‐α or endotoxin, p65 ISGylation diminishes, permissive for p65 access to phosphorylation and transcriptional activation of inflammatory genes [124]. FBXL19 knockdown increases endothelial inflammation and endothelium‐specific FBXL19 overexpression reduces it in vivo [124] (E3/E4). This work was performed in models of acute lung injury rather than atherosclerosis, so the relevance of ISGylation‐dependent restraint of NF‐κB to atherosclerotic vascular inflammation is an extrapolation.

Taken together, these studies extend the functional repertoire of ISG15 from a macrophage‐autophagy regulator to an endothelial inflammation suppressor. In these two compartments the common feature is a protective phenotype, comprising enhanced lipid clearance and dampened inflammatory signaling. Only the endothelial effect is attributable to ISGylation of a defined substrate; the macrophage effect depends on ISG15 acting through an autophagy pathway rather than on conjugation to an identified acceptor. The smooth muscle literature points in the opposite direction, and the two should not be summarized together. Targeted augmentation of ISG15 or its ligases in a cell‐type–restricted manner could be an attractive therapeutic avenue for atherosclerosis. However, careful modulation will be required to avoid off‐target immunological effects.

#### 6.8.2 ISGylation‐Associated Pathways in Vascular Smooth Muscle Cell Remodeling and Atherosclerosis Progression

Emerging evidence suggests that interferon‐stimulated genes (ISGs) and related RNA‐editing mechanisms are central regulators of vascular smooth muscle cell (VSMC) phenotype, inflammatory activation, and structural remodeling in atherosclerosis. These pathways appear to converge on a shared axis of chronic low‐grade inflammation (“metaflammation”) that links metabolic stress to vascular instability. In transcriptomic profiling of network‐driven integrative global and local protein–protein interactions in a porcine model of dual hyperglycemia and hyperlipidemia, UBE2L6 and MX1 were identified as centrality nodes with extensive atherosclerotic lesions [206] (E5). Functional enrichment implicated type I interferon signaling, micronutrient metabolism, and nucleotide biosynthesis. Whether ISG up‐regulation increases oxidative and metabolic stress in this setting was not tested; the analysis is associative [206]. Complementary mechanistic data, currently available only as a preprint (E3†), implicate the RNA‐editing enzyme ADAR1 as a brake on maladaptive ISG activation in VSMCs [217]. By converting adenosine to inosine within endogenous double‐stranded RNAs, ADAR1 prevents the aberrant activation of the cytosolic RNA sensor MDA5 and downstream interferon programs. Smooth muscle–specific deletion of Adar1 in atherosclerosis‐prone mice provoked marked phenotypic switching, extracellular matrix remodeling, and calcification pathological changes that were abolished in double knockouts lacking MDA5 [217]. Single‐cell RNA sequencing suggested that loss of RNA‐editing restraint may contribute to the emergence of an inflammatory VSMC state associated with reduced plaque stability [217]. In addition to upstream repression of regulation, ISG15 itself has direct regulatory influence on VSMC phenotype. Ablation of ISG15 by experimentation increased contractile and synthetic program gene expression at the expense of osteogenic markers and calcification [125]. Systemic in vivo injection of recombinant ISG15 produced hypotrophic remodeling and inhibited contractile responsiveness, whereas ISG15 deficiency increased vasoconstrictive tone [125]. Specifically, analysis of human abdominal aortic aneurysm tissue samples illustrated a correlation between ISG15 expression and canonical markers of contractility, indicating that ISG15 may be involved in human vascular disease beyond the atherosclerotic setting [125].

Collectively, these findings delineate a mechanistic continuum in which ISG pathway overactivation via transcriptional induction (MX1, UBE2L6), loss of RNA‐editing restraint (ADAR1 deficiency), or direct effector function (ISG15) drives maladaptive VSMC remodeling, chronic vascular inflammation, and lesion destabilization. Notably, the biological effect of ISG15 action is setting‐dependent: facilitating inflammatory remodeling under conditions of chronic metabolic stress but in acute contexts suppressing contractile tone and causing structural hypotrophy.

### 6.9 ADP‐Ribosylation in Atherosclerosis: A Multifaceted PTM in Plaque Pathogenesis

#### 6.9.1 PARP1‐Driven Vascular Remodeling

PARP1 has emerged as a pivotal coordinator of vascular smooth muscle cell (VSMC) fate, coordinating DNA damage response, chromatin remodeling, and transcriptional reprogramming to regulate atherosclerosis, calcification, and vascular aging. In diverse pathological conditions, diabetic arteriosclerosis, typical atherosclerosis, and progeroid vascular disease PARP1 activation all point towards the same maladaptive cell programs: repression of the contractile phenotype, induction of synthetic and osteogenic transitions, and amplification of cell loss through NAD^+^ depletion.

In diabetic atherosclerotic calcification, PARP1 mediates Stat1‐induced transcription of Runx2, a master osteogenic regulator, in VSMCs and macrophages, thus promoting plaque calcification and vessel stiffening. PARP1 inhibition blocks these actions, inhibiting osteogenic differentiation and vascular compliance [126]. Mechanistically, this is paralleled in oxidative stress–driven phenotypic switching, where PARP1‐dependent activation of the DNA hydroxymethylase TET1 increases 5hmC levels at loci governing the synthetic phenotype, and its inhibition rescues contractile gene expression [127]. In both models, PARP1 acts upstream of transcriptional effectors (Runx2, TET1) that irreversibly commit VSMCs to pathological states.

Recent mechanistic mapping has revealed that PARP1 directly poly(ADP‐ribosyl)ates myocardin and serum response factor, dismantling the CArG‐box transcriptional complex and enabling c‐Jun–mediated transcription of proliferation and migration genes [128]. This dual action, suppression of contractile identity and activation of synthetic, proliferative programs, provides a unifying explanation for its role in lesion expansion and restenosis [128]. In contrast, in Hutchinson‐Gilford progeria syndrome (HGPS), hyperactivation of PARP1 connects progerin accumulation with disastrous VSMC loss through NAD^+^ depletion. Trifluridine‐based pharmacologic inhibition of PARP1 not only replenishes NAD^+^ but also maintains VSMC viability, aortic degeneration being avoided [47]. Additional in vivo evidence reinforces this bidirectionality. NRP2‐driven PARP1 upregulation under low shear stress promotes endothelial apoptosis and lesion development in *ApoE* ^−^/^−^ mice, and NRP2 knockdown reduces PARylation and apoptosis‐related gene expression [189]; conversely, PARP1 inhibition with 3‐aminobenzamide lowers hepatic lipid loading through FXR/BSEP‐dependent bile acid metabolism [190], and geniposide restores lipophagy by suppressing PARP1/PI3K/AKT signaling, an effect abolished by PARP1 overexpression [191]. The paradoxical increase in plaque burden following NAD^+^ supplementation [48] was examined as cofactor‐coupled cross‐talk in Section 7.1 and is not repeated here.

Together, these investigations define a mechanistic continuum: PARP1 facilitates vascular disease by destabilizing safe‐keeping transcriptional complexes, causing maladaptive phenotypic switching, and draining the metabolic reserves needed for survival by VSMCs. Therapeutically, this identifies two complementary avenues: (i) enzymatic inhibition of PARP1 to restore contractile identity and arrest synthetic/osteogenic transitions and (ii) metabolic preservation by NAD^+^ salvage support.

#### 6.9.2 ADP‐Ribosylation: A Pivotal Regulator of Inflammation and Lipid Dysregulation in Atherosclerosis

ADP‐ribosylation, the transfer of ADP‐ribose from NAD^+^ to acceptor residues by poly(ADP‐ribose) polymerases (PARPs), modulates important pathogenic events in atherosclerosis, including endothelial apoptosis, lipid metabolism, and inflammation. In ApoE^–/–^ mice, neuropilin 2 (NRP2) initiates PARP1 upregulation under low shear stress conditions, causing endothelial apoptosis and plaque development, whereas NRP2 knockdown represses atherosclerosis by decreasing PARP1‐initiated PARylation and the gene expression of apoptosis‐related genes [189]. In contrast, inhibition of PARP1 by 3‐aminobenzamide (3‐AB) in ApoE^–/–^ mice and Sprague Dawley rats lowers lipid loading in the liver by improving bile acid metabolism by upregulation of farnesoid X receptor (FXR) and bile salt export pump (BSEP), with reduced gut microbiota with bile salt hydrolase, indicating a gut‐liver axis in plaque reduction [190]. Similarly, geniposide improves atherosclerosis in ApoE^–/–^ mice by reversing lipophagy impairment via inhibition of PARP1/PI3K/AKT signaling, inhibiting foam cell formation and lipid accumulation, whose impact is negated by PARP1 overexpression [128]. In contrast, NAD^+^ supplementation in ApoE^–/–^ mice paradoxically raises plaque area and inflammation, mediated by CD38 up‐regulation and redirection of NAD^+^ usage towards PARP1 away from anti‐inflammatory sirtuins, with elevated pro‐inflammatory metabolite 4PY levels [48]. These results demonstrate a two‐sided activity for ADP‐ribosylation: pro‐atherogenic through PARP1‐induced apoptosis and inflammation, but susceptible to therapeutic inhibition by inhibiting PARP1 or promoting lipophagy. These findings suggest that and NAD^+^‐sparing interventions could shift the balance, but the unexpected pro‐atherogenic action of NAD^+^ supplementation shows the importance of careful metabolic modulation. Future research would be aimed at human plaque proteomics to confirm these mechanisms and investigate selective PARP1 inhibitors to limit off‐target effects. The diverse pro‐ and anti‐atherogenic roles of ADP‐ribosylation across vascular, immune, and metabolic compartments, including PARP1‐driven remodeling, PARP14‐dependent ferroptosis, and context‐dependent modulation of lipid metabolism are consolidated in Table 6.

## 7 Mechanistic Cross‐Talk Between Posttranslational Modifications

Sections 6.1–6.9 treat modification classes separately because the primary literature is organized that way, not because vascular cells regulate them separately. This section reverses the organization. Cross‐talk is defined here not as co‐occurrence within a pathway, which is merely convergence, but as a demonstrable dependency between two modifications. Four such dependencies account for the documented cases, each with a distinct experimental signature. In competitive cross‐talk, two modifications contest one acceptor residue and are mutually exclusive on a given protein molecule; the signature is that the substitution of the acceptor abolishes both marks [24, 27, 39, 40, 41, 42]. In hierarchical cross‐talk, one modification creates or destroys the recognition determinant for the enzyme that installs another; the signature is that a nonmodifiable mutant of the first mark abolishes the second [44]. In enzyme‐directed cross‐talk, a modification acts on the writer, eraser, or reader of a different system rather than on a shared substrate, as when HDAC9 deacetylates IκB kinases to sustain phosphorylation‐dependent NF‐κB signaling [21]. In cofactor‐coupled cross‐talk, two chemically unrelated modifications draw on one limiting metabolite and are therefore nonindependent, despite sharing neither substrate nor enzyme [48]. Table 7 assigns every relationship discussed below to one of these categories, records the substrate and, where known, the acceptor residue, and grades the evidence using the scheme described in Section 5. Two features of that table govern what follows: the vascular literature is rich in enzyme‐directed examples and almost devoid of residue‐resolved competitive ones, and most residue‐resolved cross‐talk invoked in vascular reviews, including this one, has been established outside the vasculature.

### 7.1 Cofactor‐Coupled Cross‐Talk: The NAD^+^‐Dependent Deacylation–PARylation Axis

Sirtuins are NAD^+^‐dependent deacylases [45, 46], and PARP1 consumes NAD^+^ to synthesize poly(ADP‐ribose) [44, 47]; because the pool is shared and finite, the activity of one system constrains the other without any shared substrate. This relationship is invisible to the substrate‐mapping methods used for every other cross‐talk category, and it is the only category for which the vascular literature contains a direct in vivo demonstration. NAD^+^ supplementation in Apoe‐deficient mice increased plaque area and inflammation rather than reducing them, with CD38 upregulation, redirection of NAD^+^ toward PARP1 and away from sirtuins, and elevated levels of the pro‐inflammatory metabolite 4PY [48] (E3). This result inverts the prediction that follows from the sirtuin literature considered alone, in which SIRT1 activation restores PGC‐1α deacetylation and mitochondrial function [45], SIRT1 loss drives M1 polarization through TIMP3/ADAM17 imbalance [46] and worsens foam cell formation through Atg5 hyperacetylation [163], and SIRT2 restrains M1 polarization and lesion growth [51]. Read together with the PARP1 literature, in which PARP1 inhibition restores contractile gene expression in VSMCs [127, 128] and trifluridine‐based PARP1 inhibition replenishes NAD^+^ and preserves VSMC viability in a progeroid model [47], the actionable unit at this node is the NAD^+^ pool rather than either enzyme. The interpretation is falsifiable: if the deleterious effect of NAD^+^ boosting reflects cofactor redirection rather than an independent PARP1 effect, then NAD^+^ supplementation combined with PARP1 inhibition should reduce, not increase, lesion burden. That experiment has not been reported, and we regard it as the highest‐yield single experiment identified in this review.

### 7.2 Competitive Cross‐Talk on Serine and Threonine: Phosphorylation and O‐GlcNAcylation

Phosphate and N‐acetylglucosamine are transferred to the same serine and threonine hydroxyls; therefore, the two marks are mutually exclusive at any one residue [39, 40, 41]. The endothelium is the only vascular instance in which this has been demonstrated at the residue resolution. In aortic endothelial cells and diabetic aorta, hyperglycemia increased O‐GlcNAc modification of eNOS and reciprocally decreased phosphorylation at Ser1177, the Akt site; both changes were reversed by antisense inhibition of glutamine:fructose‐6‐phosphate amidotransferase or by suppressing mitochondrial superoxide with UCP‐1 or MnSOD; glycosylated eNOS had lower catalytic activity than the phosphorylated enzyme; and eNOS mutated at the Akt site showed no hyperglycaemia‐induced change in O‐GlcNAc, establishing that the two modifications contest the same residue [196] (E4‡). This provides a defined biochemical mechanism for the loss of nitric oxide bioavailability that much of the vascular literature, including Sections 6.1.3 and 6.4.2 of this review, treats as an endpoint [119, 132, 133].

In vascular smooth muscle, the equivalent relationship is documented at the pathway rather than the residue level. OGT‐dependent O‐GlcNAcylation of β‐catenin increases its stability and transcriptional activity, and smooth‐muscle‐restricted Ogt deletion attenuates phosphate‐induced osteogenic conversion and mineral deposition, an effect reversed by pharmacological Wnt activation [19] (E3); smooth‐muscle Ogt deletion likewise reduces lesion burden and restores contractile markers in hyperglycemic Apoe‐deficient mice, with reduced YY1 and SRF expression [120] (E3). Because O‐GlcNAc and phosphate compete for the same hydroxyls [39, 40, 41], occlusion of phosphorylation sites is a plausible mechanism for β‐catenin stabilization, but the acceptor residues have not been mapped in VSMCs and no phosphorylation‐site mutant has been tested; the cross‐talk is therefore inferred, not demonstrated. More direct mechanistic evidence supports the O‐GlcNAc–AKT–Runx2 axis. Heath et al. demonstrated that O‐GlcNAcylation of AKT at Thr430 and Thr479 promotes AKT Ser473 phosphorylation and mTORC2 binding. Combined site‐directed mutation of Thr430 and Thr479 reduced AKT O‐GlcNAcylation, inhibited Ser473 phosphorylation and mTORC2 binding, and subsequently blocked Runx2 transactivity and vascular smooth muscle cell calcification (E3‡) [138]. However, because the two residues were mutated together, their individual contributions were not resolved.A human observation constrains extrapolation in the opposite direction. In the China PEACE‐MPP cohort, low serum OGT activity was associated with a higher incidence of major adverse cardiovascular events, and higher OGT activity with a 26% reduction after adjustment [207] (E2). Systemic OGT activity and smooth‐muscle O‐GlcNAc cycling therefore cannot be treated as the same variable, and neither the mouse nor the cohort data can be used to predict the effect of a systemic OGT‐directed agent (Figure 6).

**Figure 6:**
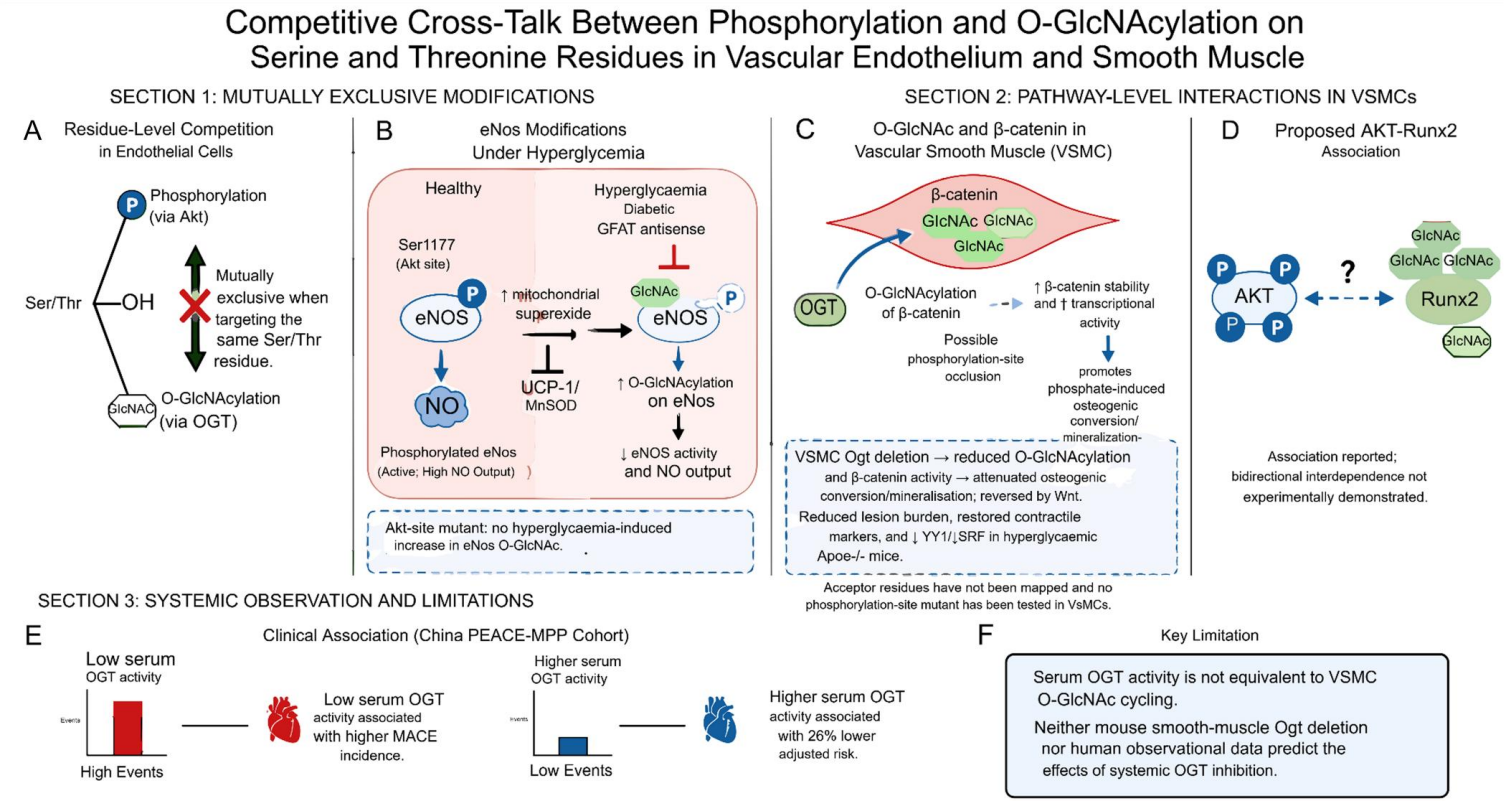
Competitive cross‐talk between phosphorylation and O‐GlcNAcylation in vascular cells**.** Phosphorylation and O‐GlcNAcylation are mutually exclusive when they target the same serine/threonine residue (A). In endothelial cells, hyperglycaemia increases eNOS O‐GlcNAcylation and decreases Ser1177 phosphorylation, reducing eNOS activity and NO output; these changes are reversed by GFAT inhibition or suppression of mitochondrial superoxide (B). In VSMCs, OGT‐dependent β‐catenin O‐GlcNAcylation increases β‐catenin stability and transcriptional activity, whereas smooth‐muscle Ogt deletion attenuates osteogenic conversion and vascular lesion‐associated changes (C). O‐GlcNAcylation of AKT at Thr430/Thr479 promotes Ser473 phosphorylation and Runx2 transactivation, and combined substitution of both residues blocks Runx2 transactivation and calcification; the individual contribution of each residue is unresolved (D). Serum OGT activity is observationally associated with cardiovascular outcomes, but cannot be equated with VSMC O‐GlcNAc cycling or used to predict the effects of systemic OGT inhibition (E–F).

### 7.3 Competitive Cross‐Talk on Lysine: Ubiquitination, SUMOylation, and Acetylation

The lysine ε‐amino group is the acceptor for acetyl, ubiquitin, SUMO, and ISG15; therefore, these marks are mutually exclusive at a given residue [24, 27, 42, 113]. The definitive demonstration concerns a substrate central to this review. SUMO‐1 is conjugated to IκBα by Ubc9 primarily at Lys21, the residue also used for ubiquitin; SUMO‐modified IκBα cannot be ubiquitinated and resists proteasomal degradation, and SUMO‐1 overexpression suppresses signal‐induced NF‐κB‐dependent transcription. Conversely, IκBα SUMOylation is inhibited by Ser32/Ser36 phosphorylation, which is required for its ubiquitination [197] (E4‡). Therefore, one substrate carries both competitive cross‐talk, SUMO against ubiquitin at Lys21, and hierarchical cross‐talk, phosphorylation licensing ubiquitination while excluding SUMOylation. This places the IKK complex, and therefore HDAC9, immediately upstream of a SUMO–ubiquitin switch: HDAC9‐mediated deacetylation of IKKα/β activates IKK and promotes plaque vulnerability [21], so an acetylation event two steps upstream sets the ubiquitination‐to‐SUMOylation ratio on IκBα. This is the mechanistic content of the statement, made repeatedly in the vascular literature, that acetylation and phosphorylation “cooperate” at NF‐κB.

Against this standard, the vascular SUMOylation literature is uniformly inferential. CBX4‐dependent SUMOylation of HIF‐1α increases its transcriptional activity and lesion burden in macrophages [43]; SENP3‐dependent removal of SUMO2/3 from NLRP3 restrains inflammasome activation and foam cell formation [38]; SUMOylation of TFEB suppresses lysosomal biogenesis and promotes foam cell maturation [200]; SUMOylation of SRF promotes VSMC phenotypic switching and matrix deposition [139]; and SUMO‐1 conjugation of SERCA2a preserves Ca^2+^ handling under diabetic stress [201]. In none of these studies, the acceptor lysine was examined for ubiquitin occupancy. The widely repeated opposition between SUMO and ubiquitin at these substrates is an inference from chemistry rather than a measurement.

One study in this field demonstrated what residue resolution yields, although the mechanism is hepatic and systemic rather than vascular cell‐autonomous. Mutation of the LRH‐1 SUMO acceptor Lys289 (K289R) abolished the LRH‐1–PROX1 corepressor interaction, increased reverse cholesterol transport, and reduced lesion burden in hypercholesterolemic mice [20] (E3‡). This is the standard that vascular SUMO literature should meet, and the fact that it was achieved in a hepatic compartment shows that the limitation is one of design rather than of technique.

Acetylation and ubiquitination are subject to the same constraint, and the vascular literature has not yet addressed this question. PPARγ is stabilized by USP22‐dependent deubiquitination, supporting efferocytosis and plaque stability [185]; is destabilized by NEDD4L‐dependent ubiquitination, driving M1 polarization and foam cell formation [202]; and NEDD4L knockdown suppresses PPARγ ubiquitination while raising ABCA1 and ABCG1 [203]. Whether the ubiquitin‐accepting lysines on PPARγ overlap with acetylation sites has not been tested in vascular cells. The sharpest available case is NLRP3, which is deacetylated by HDAC9 at its NACHT and LRR domains to permit inflammasome activation and pyroptosis in human carotid plaque [115], and deSUMOylated by SENP3 to restrain inflammasome activation in macrophages [38]. NLRP3 is thus a documented substrate of two lysine‐directed erasers with opposite phenotypic consequences. If the marks occupy overlapping lysines, the two enzymes are in direct competition; otherwise, they are parallel inputs. The experiment required, site‐resolved mass spectrometry of NLRP3 from macrophages after HDAC9 or SENP3 manipulation, is entirely feasible and has not been done.

### 7.4 Hierarchical Cross‐Talk at NF‐κB p65: Phosphorylation Licenses Acetylation, ISGylation Reverses Both

The NF‐κB–IKK axis was designated Tier 1 in Section 4 on the basis of PTM‐class convergence. This convergence is not a set of parallel inputs but an ordered circuit, and the ordering has therapeutic consequences. Macrophage WEE1 binds and phosphorylates NF‐κB p65 at Ser536, driving inflammatory transcription and lesion progression [32] (E3). Phosphorylation at Ser536 and Ser276 increases the assembly of p65 with p300 and thereby enhances acetylation at Lys310; reconstitution of RelA‐deficient cells with an S536A mutant reduces TNF‐induced Lys310 acetylation and expression of the NF‐κB target E‐selectin [198] (E4‡). Acetylation at this node is therefore downstream of phosphorylation rather than parallel to it. In the vascular literature, the acetyltransferase step is instantiated by CBP: CBP‐dependent acetylation of NF‐κB accompanies thrombin‐induced endothelial activation, and CBP knockdown reduces NF‐κB acetylation, VCAM‐1, ICAM‐1, and E‐selectin expression, and leukocyte–endothelial adhesion [52] (E4). The endogenous brake operates on the licensing step rather than on acetylation itself. SCF^FBXL19^‐mediated ISGylation of p65 stabilizes its interaction with the phosphatase WIP1 and suppresses inflammatory serine phosphorylation and nuclear translocation; FBXL19 knockdown increases and endothelial FBXL19 overexpression decreases inflammation in vivo [124] (E3/E4). WIP1 is a direct phosphatase of p65 Ser536, and WIP1‐mediated dephosphorylation reduces p65–p300 binding and chromatin recruitment [199] (E4). Combining these, ISGylation of p65 should suppress Lys310 acetylation indirectly, by removing the phosphorylation that recruits p300, a three‐modification circuit, ISGylation ⊣ phosphorylation → acetylation, in which no two marks share a residue and each step is separately druggable. Whether FBXL19‐dependent ISGylation reduces p65 Lys310 acetylation in endothelial cells is answerable with existing reagents and, to our knowledge, has not been asked. A fourth layer acts on the enzymes rather than the transcription factor: HDAC9 deacetylates IKKα/β to sustain IKK activity and promote plaque vulnerability [21], and also deacetylates NLRP3 to permit inflammasome activation and pyroptosis [115]. A single class IIa deacetylase therefore sets both the kinase that initiates the circuit and a downstream effector, which explains why class IIa HDAC inhibition produces broader phenotypic effects than inhibition of any single kinase in the same pathway [21, 115, 116].

Two limitations qualify the circuit. The licensing relationship between Ser536 phosphorylation and Lys310 acetylation was established in fibroblasts [198] and the WIP1–p65 relationship in epithelial and immune cells [199]; neither has been reproduced in endothelial cells, macrophages, or VSMCs. Moreover, the directionality of Ser536 phosphorylation is context‐dependent outside the vasculature, so the pro‐inflammatory assignment used here rests on the macrophage data [32] rather than on the modification per se (Figure 7).

**Figure 7:**
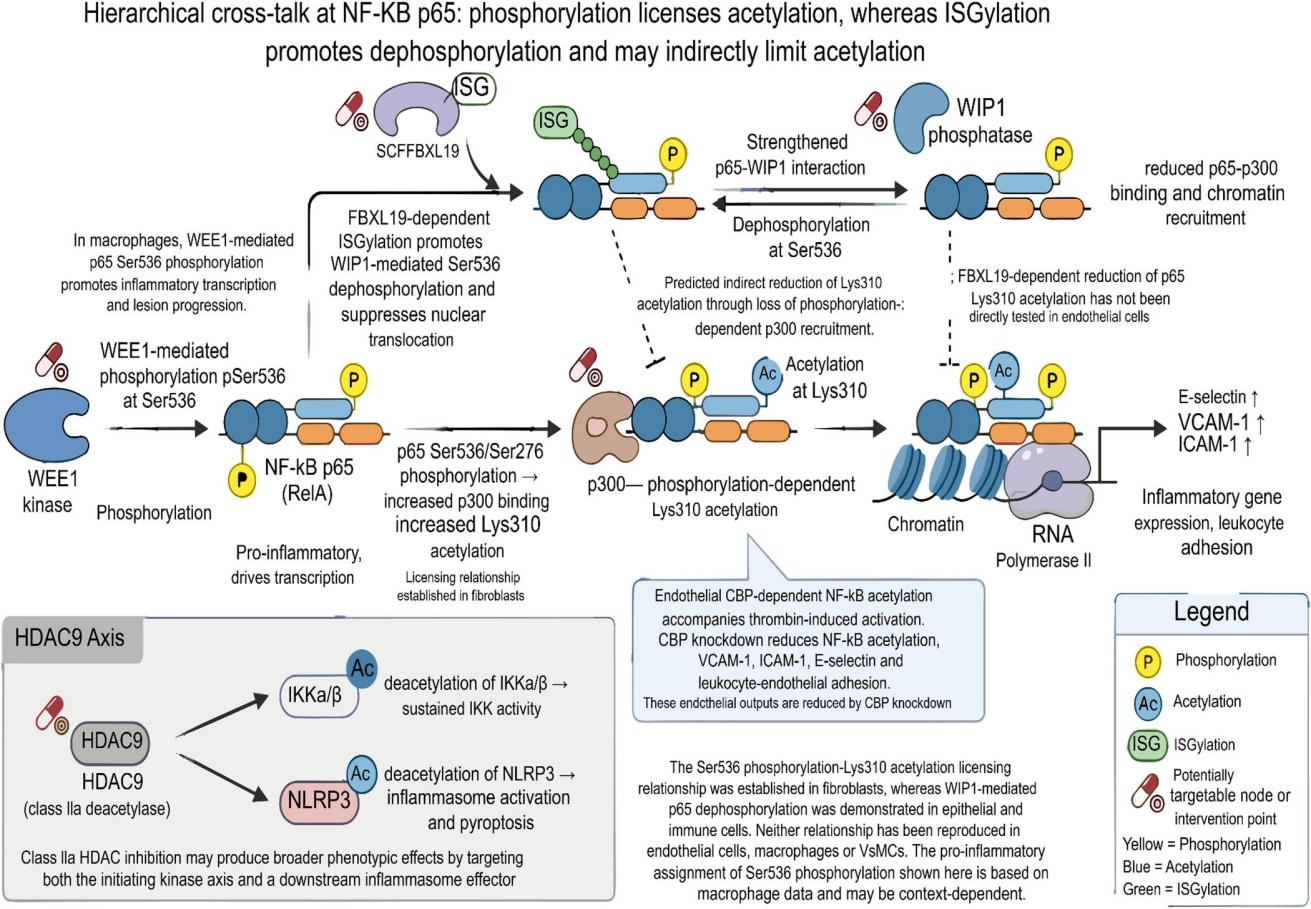
Hierarchical PTM cross‐talk at NF‐κB p65. WEE1‐mediated Ser536 phosphorylation promotes inflammatory transcription, while Ser536/Ser276 phosphorylation enhances p300 binding and Lys310 acetylation. FBXL19‐dependent ISGylation strengthens p65–WIP1 interaction, promoting Ser536 dephosphorylation and reducing p65–p300 binding and chromatin recruitment; its effect on Lys310 acetylation remains inferred. HDAC9 additionally deacetylates IKKα/β and NLRP3, linking sustained kinase activity with inflammasome activation; the indicated relationships are cell‐type and context dependent.

### 7.5 Enzyme‐Directed Cross‐Talk: Modification of the Writers and Erasers

Crosstalk does not require a shared substrate. When a modification acts on the enzymatic machinery of another system, the two are coupled at the level of capacity rather than occupancy, and the vascular literature contains several well‐controlled examples. AMPKα2 restrains GPR120 SUMOylation by suppressing UBC9, the SUMO E2, and is required for the anti‐atherosclerotic effect of ω−3 fatty acids. AMPKα2 loss produces GPR120 hyper‐SUMOylation, disrupts β‐arrestin coupling, and abolishes the benefit, with concordant inverse AMPKα2–UBC9 relationships in human arteries [204] (E3 with E2 support). This is the cleanest instance in the vascular literature of a kinase controlling the SUMO conjugation machinery itself. In the reciprocal direction, chemerin acting through β_2_‐integrin triggers global SUMO2/3 conjugation in monocytes, SUMOylates HSP90AB1, and drives ERK1/2 phosphorylation and pro‐adhesive activation, all reversed by the SUMO E1 inhibitor ML‐792 [205] (E4).

Kinase cascades also set deacetylase activity. Nε‐carboxyethyl‐lysine dysregulates RAGE/LKB1/AMPK/SIRT1 signaling and thereby alters ZKSCAN3 acetylation and macrophage autophagy, destabilizing plaque in diabetic atherosclerosis [50] (E3), a phosphorylation cascade determining the acetylation state of a transcription factor two steps downstream. Lipidation performs the same function for the AKT axis: Zdhhc1‐dependent palmitoylation of PI3K p110α determines its subcellular distribution and thereby the amplitude of AKT–mTOR phosphorylation, and Zdhhc1 deficiency reduces oxLDL uptake, foam cell formation, and lesion burden [121] (E3). Palmitoylation is rarely discussed as cross‐talk, but functionally it is enzyme‐directed cross‐talk onto the phosphorylation system.

Finally, ubiquitination controls ADP‐ribosylation in macrophages: TRIM25‐mediated ubiquitination of XRCC1 releases PARP1 activity and drives M1 polarization and programmed cell death, and TRIM25 loss reduces lesion burden and fibrosis [118] (E3). This is the only documented ubiquitination–ADP‐ribosylation link in the vascular literature. The converse direction, poly(ADP‐ribose) chains serving as the recognition determinant for PAR‐directed ubiquitin ligases, is well characterized biochemically [44] but has not been examined in vascular cells, despite PARP1 being a Tier 1‐adjacent node in VSMCs [47, 126, 127, 128].

### 7.6 Shared Enzymes and Shared Substrates: Two Structural Sources of Nonindependence

Two situations generate crosstalk without either a shared residue or a regulatory modification, and both are present and unacknowledged in the studies reviewed above. Shared enzymes. TRIM25 is an E3 ligase for both ubiquitin and ISG15 [142]; therefore, the atheroprotective phenotype of TRIM25 loss [118] cannot be attributed to ubiquitination without excluding an ISGylation contribution. UBE2L6/UbcH8, identified alongside MX1 as a network hub in a porcine model of combined hyperglycemia and hyperlipidemia [206], is the E2 shared by the ubiquitin and ISG15 conjugation cascades [142], and its centrality in that network is uninterpretable at the level of a single modification. Most consequentially, USP18 has been reported to promote cholesterol efflux and mitigate atherosclerosis by deubiquitinating ABCG1 [137]. However, USP18 is characterized enzymologically as an ISG15‐specific isopeptidase that removes ISG15 from conjugates and has essentially undetectable activity toward ubiquitin conjugates [142, 186]. The stabilization of ABCG1 may be indirect, ISG15‐dependent, or USP18 may possess a vascular activity not observed in other systems. The distinction is material rather than pedantic because ISG15 independently promotes ABCA1‐dependent cholesterol efflux in macrophage foam cells through miR‐17‐5p/Beclin‐1‐dependent autophagy [123]. The two ISG15‐linked efflux findings may describe one mechanism rather than two, and this review has treated them as separate.

Shared substrates. SRF is poly(ADP‐ribosyl)ated by PARP1, which dismantles the myocardin–SRF CArG‐box complex and licenses c‐Jun‐driven proliferative and migratory transcription [128]; SRF is SUMOylated, which promotes the contractile‐to‐synthetic transition and matrix deposition [139]; and smooth‐muscle Ogt deletion reduces SRF expression together with YY1 [120]. Three modification systems converge on a single transcription factor that specifies the contractile phenotype, and no study has manipulated more than one of them at a time. According to the criteria used in this review, SRF is the highest‐value substrate for combinatorial PTM mapping in vascular smooth muscle. The cholesterol efflux machinery is the corresponding node in macrophages: ABCA1 is stabilized by Listerin‐dependent Lys63‐linked ubiquitination [182], ABCG1 by USP18 [137], both are increased by NEDD4L knockdown [203], and ABCA1‐dependent efflux is promoted by ISG15‐driven autophagy [123]. However, it remains unclear whether these constitute one regulatory module or four independent modules.

### 7.7 What Is Demonstrated, What Is Inferred, and What Would Settle It

Applying the grading scheme of Section 5 to Table 7 yields three conclusions that should govern how the preceding sections are read. First, of all the cross‐talk relationships invoked in this review, only five are supported at residue resolution by a substitution experiment: the eNOS Akt site [196], IκBα Lys21 with its Ser32/Ser36 phosphodegron [197], p65 Ser536 licensing Lys310 acetylation [198], AKT Thr430/Thr479 licensing Ser473 phosphorylation and Runx2 transactivation [138], and the LRH‐1 SUMO acceptor Lys289 [20]. Of these, only two were obtained in vascular cells, the eNOS Akt site in endothelium and the AKT Thr430/Thr479 site in calcifying smooth muscle; the IκBα and p65 relationships were established in nonvascular cell systems and their vascular relevance is an extrapolation, and the LRH‐1 result is hepatic and systemic. Two further qualifications apply: the AKT threonines were mutated together, so their individual contributions are unresolved, and at the LRH‐1 site competition with ubiquitin was never tested, so the residue mapping establishes SUMO dependence rather than SUMO–ubiquitin competition.

Second, the field is not technically incapable of residue resolution; it simply has not been applied to *pairs* of modifications. Single‐modification residue mapping with functional rescue exists, including LRH‐1 K289R with a lesion‐burden readout [20], SR‐BI Lys500/Lys508 with hepatocyte‐specific genetic controls [213], Listerin‐dependent ubiquitination of ABCA1 at Lys1884/Lys1957 [182], endothelial expression of palmitoylation‐deficient PKM2 C31S [215], and the mapped NLRP3 acceptor domains [115]. Extending these designs to combinatorial mutants is an incremental step rather than a conceptual one. Third, cofactor‐coupled cross‐talk is invisible to residue mapping and requires metabolic rather than proteomic experiments; it is the only category in which a vascular study has shown that the interaction, rather than either enzyme alone, determines the phenotype [48].

We propose four criteria that a claim of PTM cross‐talk in vascular disease should satisfy: (i) both modifications detected on the same substrate in the same cell type, preferably by site‐resolved mass spectrometry; (ii) the acceptor residue or residues mapped; (iii) substitution of the acceptor abolishing the second modification or its phenotype; and (iv) for competitive claims specifically, evidence that the two marks are mutually exclusive on individual molecules rather than merely anticorrelated in bulk lysate. Criterion (iv) is almost never attempted, and is the point at which most competitive claims in this literature would fail if tested [26, 31]. This grading also disciplines the therapeutic proposals developed in Section 8. Co‐targeting is justified where the relationship is enzyme‐directed or cofactor‐coupled, because these do not require the two marks to meet on one molecule: combined PARP1 inhibition with NAD^+^ repletion in calcifying, VSMC‐dominant lesions [47, 48, 127, 128], or Zdhhc1 inhibition together with AKT/mTOR modulation in macrophage‐dominant plaques [121, 147, 149], are hypotheses of this kind. Co‐targeting is premature where the relationship is competitive but unmapped, because the direction of the interaction is then unknown and the intervention may redistribute rather than remove the pathogenic mark; this applies to the β‐catenin [19] and NLRP3 [38, 115] nodes as currently characterized. Distinguishing these two situations, rather than cataloguing further modifications, is in our view the principal task facing the field.

## 8 Therapeutic Perspectives and Translational Challenges

PTM discoveries from mechanistic understanding to clinical application in atherosclerosis have to pass through a rational, step‐by‐step, and iterative process. It starts with mechanistic discovery, where high‐resolution mapping of PTMs on ECs, macrophages, and vascular SMCs revealed key disease nodes, such as HDAC9‐catalyzed deacetylation [21, 115, 116, 144, 145], UBA1‐catalyzed ubiquitination [146], and Zdhhc1‐driven palmitoylation [121]. Genetic and functional analyses have repeatedly associated HDAC9 variants with synthetic and inflammatory phenotypes in macrophages and VSMCs [21, 144], whereas UBA1 function controls ubiquitin conjugation modes that regulate foam cell formation and the production of inflammatory cytokines [146].

Second, biomarker validation to establish the prognostic and predictive function of candidate PTMs or their resulting downstream signatures in human cohorts is required. Multimodal strategies, such as plaque proteomics combined with imaging–histology correlation and circulating PTM‐specific peptide assays, can measure target activity in vivo. For instance, increased HDAC9 expression in carotid endarterectomy tissue is associated with thin‐cap fibroatheroma morphology [115]. In contrast, no circulating marker of ubiquitin–proteasome pathway activity has been validated in vascular disease; the ubiquitin‐directed mechanisms reviewed here [118, 182] were established in tissue and would require independent assay development before they could support noninvasive measurement. A schematic overview of candidate PTM biomarkers and emerging therapeutic strategies is depicted in Figure 8.

**Figure 8:**
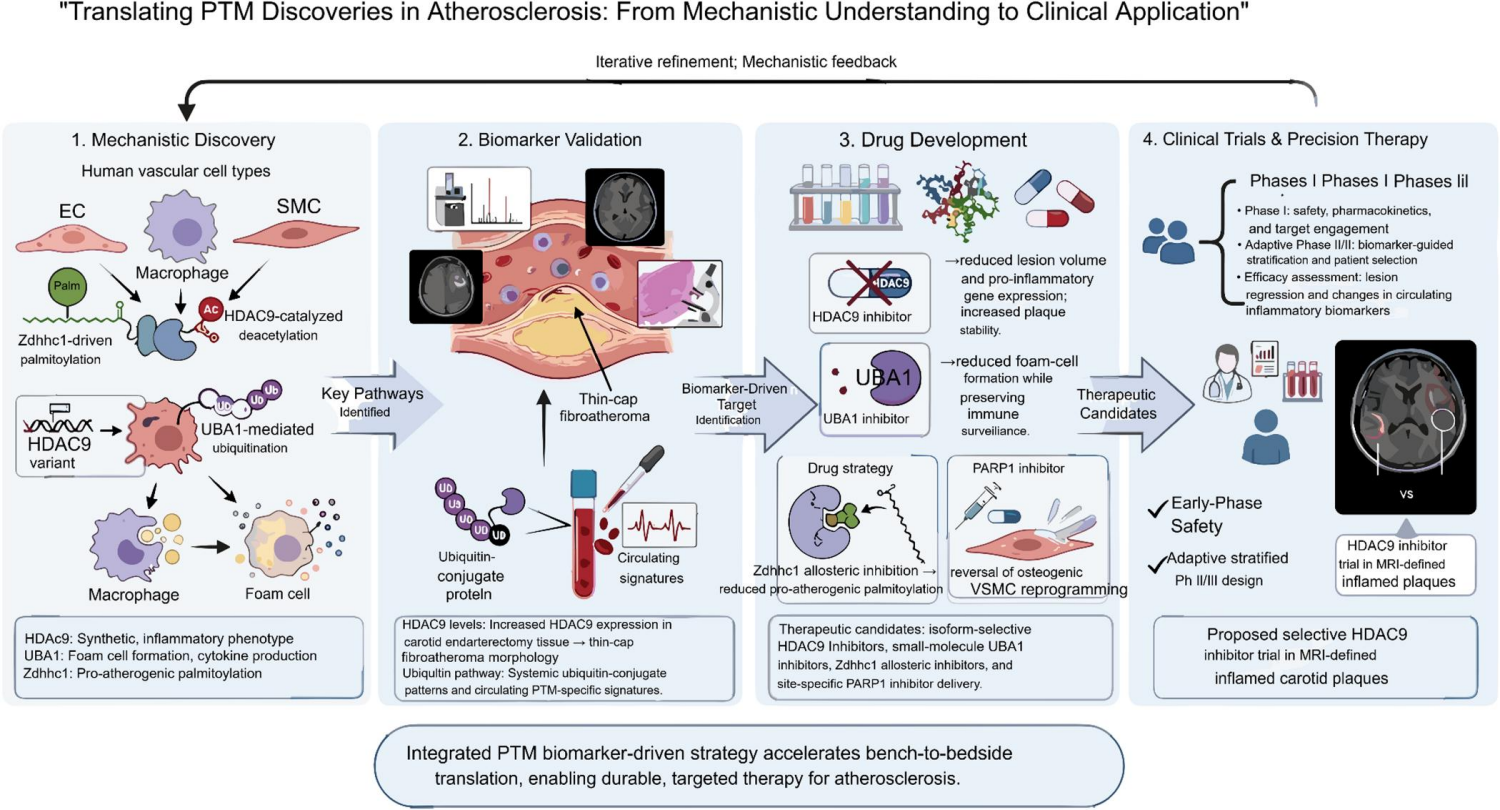
Translational framework for PTM‐targeted therapies in atherosclerosis. Mechanistic discovery in endothelial cells, macrophages, and VSMCs identifies disease‐relevant PTM nodes, including HDAC9, UBA1, and ZDHHC1, which are subsequently evaluated through plaque‐based and circulating biomarkers. Validated targets guide development of class IIa HDAC and UBA1 inhibitors, proposed ZDHHC1 allosteric inhibition, and site‐specific PARP1 inhibitor delivery; none of the agents shown is isoform‐selective for the enzyme named, and the ZDHHC1 and site‐directed strategies remain proposals.

Upon identification, they then move on to drug development. Upon identification, candidate nodes move to drug development, where the principal constraint of this field becomes visible. Class IIa HDAC inhibition with TMP195, including in nanoparticle formulation, reduces lesion growth and increases fibrous cap thickness in preclinical models [115, 116]. However, TMP195 inhibits class IIa HDACs as a group, and no HDAC9 isoform‐selective inhibitor has been reported; the atheroprotective effect cannot presently be attributed to HDAC9 alone. Small‐molecule inhibition of UBA1 with PYR‐41 reduces plaque development and macrophage cytokine production without altering systemic lipids or immune cell composition [146], although UBA1 inhibition suppresses ubiquitin activation globally and the long‐term consequences for protective immune surveillance have not been tested. Other strategies for translation involve Zdhhc1 allosteric inhibition to prevent pro‐atherogenic palmitoylation [121] and site‐specific delivery of PARP1 inhibitors to reverse osteogenic reprogramming in calcifying VSMC‐enriched lesions [47, 126, 127, 128].

The last phase, clinical trials, requires early‐phase trials to determine safety, pharmacokinetics, and target interaction with PTM‐specific biomarkers. Adaptive Phase II/III designs may stratify patients by plaque PTM profiles to support precision therapeutic deployment. An example would be a trial of a class IIa HDAC inhibitor in MRI‐defined inflamed carotid plaques, with both lesion regression and modulation of circulating inflammatory biomarkers as read‐outs [115], contingent on the prior development of an isoform‐selective agent, which does not yet exist. Integration of the four phases, mechanistic discovery, biomarker validation, drug development, and clinical trials, offers a logical sequence for translating PTM‐targeted interventions from bench to bedside. Integration of PTM biomarkers into trial design and more refined selection of patients will drive mechanistic refinement more quickly and ultimately enhance the ability to achieve durable and targeted atheroprotection with fewer systemic side effects (Table 8).

## 9 Conclusion

PTMs in atherosclerosis are not a catalog of parallel modifications but rather a small number of co‐regulated nodes. Three carry most of the mechanistic weight: the NF‐κB–IKK axis, which satisfies all four prioritization criteria and is the only node with human genetic anchoring supported by functional characterization; the NLRP3 inflammasome, admitted by stated exception because its vascular evidence is predominantly myeloid; and the NAD +‐dependent deacylation–PARylation axis, admitted despite the absence of human anchoring because it supplies the only in vivo demonstration in this field that the interaction between two modification systems, rather than either enzyme alone, determines the phenotype. What distinguishes these nodes is not the number of modifications reported on them but whether a dependency between those modifications is shown. Based on the evidence assembled here, only five such dependencies have been established by substituting a mapped acceptor residue, and only two of the five in vascular cells; the remainder rest on enzyme‐level regulation, shared chemistry, or pathway convergence. Therefore, the principal deficit of the field is evidentiary rather than descriptive. Progress will come from determining whether co‐occurring modifications are competitive, hierarchical, enzyme‐directed, or merely coincidental, and for competitive claims, whether two marks are mutually exclusive on individual molecules, not from adding further modifications to the inventory.

## Author Contributions

**Weiwei Jiang:** conceptualization, formal analysis, data curation, investigation, writing – original draft preparation. **Haiping Du:** conceptualization, methodology, investigation, resources, data curation, writing – review and editing, supervision, project administration.

## Funding

The authors have nothing to report.

## Conflicts of Interest

The authors declare no conflicts of interest.

## Data Availability

No data sets were generated or analyzed during the current study.
